# Comunicaciones LX Reunión Anual SENFC Barcelona, 2 a 4 de octubre de 2024

**DOI:** 10.31083/RN38514

**Published:** 2025-06-26

**Crisis de Ausencia con Componente Mioclónico Prominente: A Propósito de un Caso**

Pedro José Melgarejo Otálora^1^, Luis Gabriel Burgos Bustamante^1^, Laura Verna Fierro^1^, María del Carmen Martín de Miguel^1^, Julio Ignacio Prieto Montalvo^1^, Ana Paloma Polo Arrondo^1^

^1^Hospital General Universitario Gregorio Marañón, Madrid, España

**Introducción y objetivos**: Las crisis de ausencia típicas pueden asociar movimientos involuntarios sutiles. Sin embargo, cuando los movimientos son prominentes, estos plantean un diagnóstico diferencial más amplio y complejo. Se describe el caso de un niño con crisis de ausencia con componente mioclónico llamativo. **Material y métodos**: Varón de 6 años de edad remitido por episodios sugestivos de ausencias desde hace 2–3 semanas, asociando “ladeo” de la cabeza y automatismos peribucales. Se desencadenan con hiperventilación. Se realiza video-electroencefalograma (EEG) de vigilia (sistema 10/20) de 33 minutos de duración. **Resultados**: El EEG muestra actividad de fondo normal y descargas generalizadas de punta-onda aisladas y en trenes (frecuencias 2,5–3 Hz, de 4–10 segundos, espontáneos y provocados por hiperventilación), algunos se acompañan de cambios clínicos evidentes: cambio de expresión de la cara, movimientos repetitivos/rítmicos de “negación” con la cabeza, en alguna ocasión movimientos clónicos en miembro superior izquierdo, no responde a las preguntas que le hacemos, detiene la hiperventilación cuando tienen lugar durante la misma y en uno de ellos parece perder algo de tono en el tronco y al final presenta algún movimiento peribucal y/o traga. Inició tratamiento con etosuximida. Actualmente presenta menor número de crisis y no hay retraso o regresión en el desarrollo psicomotor. **Discusión/Conclusiones**: Las crisis epilépticas generalizadas de ausencia típicas pueden acompañarse de componentes mioclónicos “sutiles”. Componentes mioclónicos más llamativos plantean diagnóstico diferencial con las ausencias mioclónicas. En series de casos previas se han registrado pacientes con mioclonías faciales (83.3%) o cervicales (16.6%). La bibliografía al respecto apunta a que, sin otros datos de alarma, la presencia de mioclonías prominentes en áreas faciales y musculatura de cuello durante las crisis de ausencia no tienen por qué ensombrecer el pronóstico de estos pacientes

**Utilidad de la Redes Convolucionales Para Predecir Patrones Electroencefalográficos de Epilepsia Generalizada Idiopática**

Juan Manuel Escobar-Montalvo^1^, Fredy Escobar-Ipúz^2^, Ana María Torres^3^, Yoel Arroyo^3^, Jorge Mateo Sotos^3^

^1^Servicio de Neurología y Neurofisiología Clínica - Hospital Universitario del Henares, Madrid, España

^2^Hospital General Universitario de Ciudad Real, Ciudad Real, España

^3^Grupo Experto en Análisis Médico (Instituto de Tecnología), Universidad de Castilla-La Mancha, Cuenca, España

**Introducción y objetivos**: La epilepsia generalizada idiopática (EGI) tiene un impacto en la salud y calidad de vida de las personas, por lo que su diagnóstico y tratamiento precoz es fundamental. El electroencefalograma (EEG) es una prueba esencial en el diagnóstico de la EGI, por lo que su análisis empleando algoritmos de aprendizaje automatizado podría ayudar de forma precisa en mejorar los tiempos de diagnóstico y tratamiento de la EGI. El objetivo fue estimar la utilidad de las redes neuronales convolucionales (CNN) para la detección patrones EEG de sujetos con EGI. **Material y métodos**: Estudio observacional predictivo, realizado en un conjunto datos de prueba y entrenamiento que incluyó registros EEG de sujetos con EGI y sanos. Se usaron técnicas estandarizadas para pre-procesar y procesar la señal EEG y se construyeron modelos de CNN para clasificar patrones EEG de EGI. Posteriormente se evaluó el rendimiento y la capacidad del modelo de CNN para clasificar los partrones EEG de EGI usando métricas estandarizadas (sensibilidad, precisión, F1score y valor predictivo positivo (VPP), área bajo la curva y radar plots). Asimismo, se comparó el modelo de CNN propuesto con los resultados obtenidos por otros modelos de machine learning. El análisis de datos se realizó empleando los Softwares Matlab (versión: 9.13.0) y Phyton (versión 3.9.7). **Resultados**: Se observó que el modelo de CNN seleccionado tuvo resultados óptimos para clasificar patrones EEG de EGI (sensibilidad = 98,15%, precision = 98,04%, F1score = 98,05% y valor predictivo positive = 98,04%). Asimismo, este modelo presentó un rendimiento adecuado (índice de Youden = 98%, área bajo la curva = 98%, índice kappa = 87% y coeficiente de correlación de Matheus = 87,14%) y mostró mejores resultados globales comparativamente con los demás modelos de machine learning. **Discusión/Conclusiones**: En nuestra muestra, observamos que el algoritmo de CNN seleccionado presentó una alta validez y precisión, así como un rendimiento óptimo, para la predicción de patrones EEG de EGI.

**Patrón SREDA Atípico en Paciente de Mediana Edad y con Eventos Paroxísticos no Epilépticos Coexistentes**

Alexandra Hurubean Kapás^1^, Miriam Teresa Sánchez Horvath^1^, Giselle Arelis Yange Zambrano^1^

^1^Hospital Universitario de Cruces, 48903 Barakaldo, España

**Introducción y objetivos**: Las descargas EEG rit́micas subclińicas en adultos (SREDA) son un patroń electroencefalograma (EEG) benigno raro (prevalencia 0,04%–0,07%), descrito principalmente en vigilia y en adultos mayores (60 an os de media). Recientemente se han reportado casos en nin os y adultos jov́enes. Las caracteriśticas tiṕicas incluyen un patroń de ondas theta sinusoidales y monorrit́micas, de mayor amplitud en regioń temporo- parietal bilateral. El objetivo es monitorizar un paciente con anomaliás en el EEG ante duda de origen epileṕtico de eventos paroxiśticos que eśte presenta. **Material y métodos**: Estudio de monitorizacioń V-EEG de 8 diás duracioń en Unidad de Epilepsia de un paciente de 51 an os con episodios de desorientacioń, irrealidad y confusioń de segundos de duracioń desde hace 16 an os. En EEG de 2008 observaron crisis elećtricas y en 2021 se registraron brotes de ondas agudas con frecuencia ascendente (2–5 Hz) en ambas regiones temporales, sin correlato clińico, informados como actividad epileptiforme bitemporal. Tratamiento con lamotrigina 50/12 h desde 2008. **Resultados**: Durante el ingreso en la Unidad, se registran hasta 12 brotes/hora de actividad theta 6–7 Hz de mediano voltaje, regular, arciforme y simet́rica en regiones temporo-parietales (I > D) de 60–90 segundos, maś frecuentes en vigilia y de mayor duracioń en suen o, sin clińica subjetiva asociada. Presenta varios episodios paroxiśticos de expresioń de desagrado y sensacioń de angustia, emisioń de sonidos tipo “quejido”, afasia, y desorientacioń. Ademaś, varios episodios de hormigueo en extremidades superiores y cabeza, asociando en una ocasioń epigastralgia. Todos ellos sin anomaliás EEG ni cambios en la actividad de fondo. **Discusión/Conclusiones**: Las variantes atiṕicas de SREDA tienen un amplio espectro, pudiendo llevar a un diagnośtico errońeo de epilepsia en situaciones que coexistan eventos paroxiśticos no epileṕticos; por lo que es importante reconocerlo para evitar el impacto a nivel social, terapéutico y personal que el diagnóstico conlleva.

**El Rol Del Electroencefalograma en la Neurotoxicidad Asociada al CAR-T. Serie de Casos de un Hospital de Tercer Nivel**

Irene Carratalá Nebot^1^, Octavio Jiménez Vega^2^, Leticia Del Carmen Santana Cáceres^1^, Inmaculada Rodríguez Ulecia^2^, Eva María Amador Gil^1^, Ayoze Nauzet González Hernández^2^, Luisa María Guerra Domínguez^2^

^1^Complejo Hospitalario Universitario Insular Materno-Infantil de Gran Canaria, Las Palmas de Gran Canaria, España

^2^Hospital Universitario de Gran Canaria Doctor Negrín, Las Palmas de Gran Canaria, España

**Introducción y objetivos**: La inmunoterapia con células T con receptor quimérico de antígenos (CAR-T) es un tratamiento prometedor para enfermedades onco-hematológicas refractarias. Sin embargo, puede provocar complicaciones como el síndrome de toxicidad neurológica asociado a la terapia con células inmunoefectoras (ICANS). La literatura reciente revela que los electroencefalogramas (EEG) en pacientes con ICANS suelen ser anormales, con una pérdida del ritmo dominante posterior y lentificación de la actividad basal, además pueden aparecer anomalías como descargas epileptiforemes y patrones rítmicos o periódicos, lo más frecuente la actividad delta rítmica (RDA). El objetivo es documentar los hallazgos del EEG y su correlación con la gravedad del ICANS, para en un futuro determinar si el EEG puede predecir el riesgo de deterioro neurológico, identificando pacientes que requieran monitorización continua o seriada. **Material y métodos**: Se realizó un estudio retrospectivo y observacional de pacientes tratados con CAR-T, centrado en aquellos que desarrollaron neurotoxicidad. Se recogieron datos clínicos y su gravedad (escala ICE) y el tiempo desde el inicio del tratamiento CAR-T hasta la aparición de los síntomas y la realización del EEG. Los registros EEG fueron reanalizados y se recopilaron datos de la actividad basal y las anomalías críticas e intercríticas. **Resultados**: De los 35 pacientes tratados con CAR-T, el 48% desarrolló Neurotoxicidad. El EEG sólo se realizó en 9 casos, en 5 en menos de 24 horas y en solo 1 hubo un seguimiento con EEG. En 8 casos se registró una lentificación de la actividad basal, en 2 se objetivó una RDA y hubo 1 caso de crisis electro-clínica. **Discusión/Conclusiones**: El EEG puede ser una herramienta útil para evaluar la gravedad la neurotoxicidad y detectar pacientes candidatos a monitorización EEG. Sin embargo, actualmente no se incluye en el protocolo del CAR-T de nuestro hospital. Por lo que, es preciso integrarlo para poder documentar su correlación con la clínica y su valor en el seguimiento de esta complicación.

**Cuantificación de la Necesidad de Monitorización Mediante Electroencefalografía Continua en un Hospital de Tercer Nivel**

Octavio Jiménez Vega^1^, Inmaculada Rodríguez Ulecia^1^, Amanda Labrador Rodríguez^1^, David Cañizo García^1^, Jose Juan Rodríguez Betancor^1^, Dolores Mendoza Grimón^1^, Ayoze Nauzet González Hernández^1^

^1^Hospital Universitario de Gran Canaria Doctor Negrín, Las Palmas de Gran Canaria, España

**Introducción y objetivos**: El uso del electroencefalograma continuo (cEEG) es una herramienta crucial para la monitorización de pacientes con distintas afecciones neurológicas. La importancia del cEEG lo convierte en una arma eficaz en la detección temprana del Estatus Epiléptico No Convulsivo (NCSE), evaluar el estado sedación, detectar la isquemia cerebral retardada y valorar la encefalopatía post-resucitación. La monitorización continua permite una mayor seguridad, eficacia, tratamiento y management del paciente neurocrítico respecto al EEG basal seriado. Sin embargo, su uso aún no está muy extendido en el territorio español. **Material y métodos**: Se analizó la viabilidad de un programa de cEEG en nuestro hospital (que cuenta solo con EEG en horario asistencial de mañana) mediante un estudio observacional y retrospectivo de todos los EEGs intrahospitalarios del año 2023 para cuantificar el número de pacientes y horas de cEEG Please provide the full name.que hubieran sido necesarias. Para ello, se realizó un análisis cuantitativo aplicando el score 2HELPS2B y un análisis cualitativo por parte de un Neurofisiólogo Clínico y un Neurólogo de todos los registros. **Resultados**: Se analizaron un total de 927 registros pertenecientes a 801 pacientes, el cEEG hubiera sido necesario en 121 casos y el déficit de horas de monitorización continua fue de 9422. El 48% de estos pacientes quedó descubierto de monitorización por fiestas o fin de semana aun cuando habría sido necesario. El 90% ingresaron en Cuidados Intensivos, incluyendo 33 casos de NCSE, 11 con elevada refractariedad. En 31 de estos 121 casos hubo discrepancia entre el criterio del Neurólogo, del Neurofisiólogo y el 2HELPS2B, siendo una discrepancia positiva a favor de una mayor monitorización a pesar de un Score negativo por indicación clínica. **Discusión/Conclusiones**: El cEEG es una herramienta esencial en el paciente neurocrítico, la monitorización continua de la actividad cerebral es fundamental para el manejo eficaz de afecciones neurológicas críticas. Su ausencia en hospitales de tercer nivel constituye una oportunidad perdida.

**Patrón de Punta-Onda Continua en Sueño Lento (POCS). Estudio Descriptivo de 22 Pacientes**

Matilde Velasco Mérida^1^, Patricia Navas Sánchez^2^, Lucía Rodríguez Santos^2^, José Miguel Ramos Fernández^2^, Victoria E. Fernández Sánchez^1^

^1^Hospital Regional Universitario de Málaga, Málaga, España

^2^Hospital Materno-Infantil de Málaga, Málaga, España

**Introducción**: El patrón POCS es un trazado electroencefalográfico característico de inicio en la infancia que puede aparecer en algunos síndromes epilépticos y en la evolución de determinadas epilepsias benignas y sintomáticas. Conlleva a la aparición de crisis epilépticas, deterioro cognitivo y comportamental, produciendo en ocasiones regresión en el neurodesarrollo de quienes lo sufren. **Objetivos**: Describir las características electroclínicas, etiológicas y evolutivas de 22 pacientes pediátricos con patrón POCS. **Material y métodos**: Pacientes y métodos. Estudio descriptivo y retrospectivo en el que se han revisado historias clínicas, datos electroencefalográficos (EEG) y polisomnográficos (PSG) de 22 pacientes pediátricos con POCS seguidos en un hospital terciario. **Resultados**: Se obtuvieron 22 pacientes, 15 varones (68,2%) y 7 mujeres (31,8%) con una edad media al diagnóstico de 6,6 años (2,66–11,01 años). El 50% presentó al inicio un índice de POCS >85%, siendo de localización focal en 6 pacientes (27,3%). El principal motivo de consulta fue la aparición de crisis epilépticas, siendo el tipo más frecuente las focales con afectación de consciencia. 8/22 pacientes presentaban alteraciones genéticas y 7/22 alteraciones estructurales. Los EEG al inicio mostraron una actividad bioeléctrica cerebral de base normal en el 40,9% y en el 90,9% anomalías epileptiformes en vigilia. **Discusión/Conclusiones**: El patrón de POCS, aunque infrecuente, representa una entidad que constituye un reto terapéutico, donde el seguimiento mediante EEG y PSG es esencial para su manejo terapéutico y seguimiento evolutivo.

**La Eficiencia del Registro Electroencefalográfico en la Encefalitis antiNMDA**

Alicia Silva Cátedra^1^, Rocío Vázquez Rodríguez.^1^, Catalina Liñán Maho^1^, Antonio Cristóbal Luque Ambrosiani^1^, Ian Mingote Madrid^1^, Francisco José Palomar Simón^1^

^1^Hospital Universitario Virgen del Rocío, Sevilla, España

**Introducción y objetivos**: La encefalitis antiNMDA cursa con una clínica inespecífica con graves secuelas. La detección precoz de la enfermedad favorece la instauración del tratamiento y mejora del pronóstico. Presentamos dos casos de encefalitis antiNMDA en los cuales el electroencefalograma (EEG) fue una prueba clave para el diagnóstico de la enfermedad. **Objetivos**: Informar sobre la importancia del EEG para el diagnóstico precoz de la encefalitis anti NMDA. Reconocer los principales patrones e interpretar la evolución electroencefalográfica. **Material y métodos**: Descripción de dos casos clínicos de pacientes ingresados por sospecha de encefalitis antiNMDA. **Resultados**: CASO 1: en el EEG se registraron 5 crisis focales de actividad punta-onda a 2 Hz en región parietotemporal izquierda de 50 segundos, con enlentecimiento posterior en rango Theta Delta. Estas crisis consistieron en alteración del lenguaje con preservación de conciencia, por lo que el EEG fue clave para descartar origen psiquiátrico e iniciar el tratamiento antiepiléptico precoz. CASO 2: se realizaron varios EEG, en los que se apreció la progresión de la enfermedad. En uno de ellos se objetivó el patrón típico en Extreme Delta Brush consistente en actividad Delta rítmica con Beta superimpuesta. En otro posterior, mientras el paciente presentaba movimientos rítmicos de miembros, se observó actividad en rango Delta sin anomalías epileptiformes, descartándose que se tratase de una crisis epiléptica y retirándose medicación innecesaria, así como confirmando que se trataba de la evolución natural de la enfermedad en la cual el paciente presenta movimientos anormales por probable afectación subcortical y de ganglios basales. **Discusión/Conclusiones**: La encefalitis antiNMDA cursa con clínica inespecífica, por lo que las pruebas aportan un alto valor diagnóstico. En estos casos la realización de un EEG permite observar alteraciones desde el inicio de los síntomas permitiendo el reconocimiento prematuro de la enfermedad así como su evolución, evitando medidas innecesarias e iniciando el tratamiento de forma precoz.

**Hallazgos Electroencefalográficos en el Síndrome Poirier-Bienvenu: A Propósito de un Caso**

David Cañizo García^1^, Inmaculada Rodríguez Ulecia^1^, Octavio Jiménez Vega^1^, Amanda Labrador Rodríguez^1^, Génesis Daniela Arteaga Requena^1^

^1^Hospital Universitario de Gran Canaria Doctor Negrín, Las Palmas de Gran Canaria, España

**Introducción y objetivos**: El síndrome Poirier-Bienvenu es una enfermedad rara y novedosa con herencia autosómica dominante que está causada por una mutación en el gen* CSNK2B*. Los pacientes presentan diversos grados de discapacidad intelectual, retraso psicomotor y epilepsia de aparición temprana. La epilepsia que presentan estos pacientes incluye todo tipo crisis, siendo la más frecuente las crisis tónico-clónicas generalizadas. Los hallazgos en el electroencefalograma tampoco son específicos, siendo la actividad epileptiforme generalizada o multifocal la más prevalente sobre una actividad de fondo que puede encontrarse enlentecida. **Material y métodos**: Presentamos el caso de un paciente con un estudio genético compatible con este síndrome, que presenta las manifestaciones fenotípicas antes mencionadas de la enfermedad, con epilepsia de aparición en el primer año de vida, con varias crisis diarias consistentes en elevación de la mirada, ligera flexión del tronco y detención de la actividad de segundos de duración. **Resultados**: En el electroencefalograma (EEG) de nuestro paciente se registró, hasta en cuatro ocasiones, actividad ictal hipervoltada en forma de punta-onda irregular de distribución generalizada, que no requirió de medicación para su yugulación. Tras el inicio de tratamiento con valproato, presentó control de las crisis y ausencia de actividad epileptiforme interictal en el EEG de control. **Discusión/Conclusiones**: Los avances en el campo de la genética han permitido identificar la etiología de cuadros neurológicos hasta ahora catalogados como idiopáticos, como es el síndrome Poirier-Bienvenu. La realización de EEG seriados en el control de crisis de estos pacientes es crucial para su manejo. La identificación del cuadro clínico es imprescindible para un diagnóstico precoz, con el fin de que estos pacientes puedan beneficiarse en un futuro de terapias génicas.

**Espectro EEG de Canalopatías de Sodio. A Propósito de un Caso**

Guillermo Ceide García^1^, Gianna Doménika Bozano Subía^1^, Paloma Balugo Bengoechea^1^, Adela Fraile Pereda^1^, Lorena Iglesias Alonso^1^, Alejandro Melcón Villalibre^1^, Octavio Ramírez Hernández^1^

^1^Hospital Clínico San Carlos, Madrid, España

**Introducción y objetivos**: La ILAE define los síndromes epilépticos como “un conjunto de características clínicas y electroencefalograma (EEG), respaldadas por hallazgos etiológicos específicos asociando un pronóstico y tratamiento a cada individuo”. Las mutaciones en los genes *SCNA* se han relacionado con encefalopatías epilépticas del desarrollo. En ocasiones, la relación entre genética y síndrome electroclínico es clara, pero en otros casos, supone un reto para el diagnóstico final del tipo de síndromes epilépticos. **Material y métodos**: Caso clínico: Se describe el caso de una paciente con un cuadro polimalformativo y retraso en el neurodesarrollo, que experimentó un inicio de crisis durante un episodio febril a los 6 meses de edad. A pesar de un EEG inicial normal, el paciente evolucionó hacia crisis focales afebriles, con un incremento progresivo en la frecuencia de las mismas y mala respuesta a múltiples fármacos anticrisis en politerapia. Los registros EEG mostraron un empeoramiento progresivo tanto intercrítico como crítico, con la aparición inicial de anomalías epileptiformes unifocales, seguidas por anomalías multifocales y crisis migratorias. Característicamente la paciente presentaba clúster de espasmos epilépticos e hipsarritmia autolimitados a episodios febriles. Este caso presentó criterios para 3 síndromes diferentes a lo largo del primer año de vida. El análisis genético reveló una deleción patogénica en las citobandas 2q24.2-q31.1, involucrando un grupo de genes relacionados con canales de sodio asociados encefalopatía epiléptica del desarrollo. La paciente mostró un control aceptable de las crisis con una combinación de FACs y dieta cetogénica. **Discusión/Conclusiones**: Ciertas canalopatías de sodio están asociadas a síndromes epilépticos específicos, aunque la variedad de las mismas podría definirse como un espectro o continuum que podría desdibujar los límites definitorios de estos síndromes.

**Hipofosfatasia Congénita y Epilepsia. A Propósito de un Caso**

Gabriela Alejandra Naranjo Heredia^1^, Raquel López-Carvajal Hijosa^1^, Madeleyn Rodríguez Jiménez^1^, Mercedes Picornell Darder^1^

^1^Hospital Universitario de Móstoles, Madrid, España

**Introducción**: La hipofosfatemia congénita es una enfermedad rara. El defecto se localiza en el gen *ALPL* en la posición 1p36.1-34 asociada a una variante heterocigota, homocigota o compuesta; que se caracteriza por acumulación de piridoxal – 5’ fosfato (PLP) que se asocia a convulsiones en relación a una deficiencia de piridoxina. mineralización anormal del hueso y del tejido dental. Las formas graves son de herencia autosómica recesiva y se presentan durante los primeros 6 meses de vida con convulsiones, desmineralización esquelética generalizada, hipotonía, creaneocionostosis, extremidades cortas e infecciones respiratorias graves. **Objetivos**: Describir las anomalías epileptiformes en los estudios video-electroencefalograma (EEG) poligráficos evolutivos. **Material y métodos**: Niño de 8 años con antecedentes de padre portador del gen de la hipofosfatasia y hermano con hipofosfatasia nacido en 2019. Que durante el desarrollo ha presentado dislalias con mejoría a los 3 años, tics motores simples, TDAH, dislexia y epilepsia frontal debut a los 9 meses, en tratamiento con diferentes antiepilépticos. **Resultados**: Hallazgos de video-EEG: Durante la vigilia la electrogénesis cerebral está desprovista de anomalías específicas. Durante el sueño REM muestra anomalías multifocales epileptiformes intercríticas (brotes paroxísticos de ondas agudas y ondas lentas/punta-onda) en áreas Fronto-Centrales/Fronto-Centro-Temporales bilaterales o de predominio en uno u otro hemisferio. No se registran crisis epilépticas. **Discusión/Conclusiones**: La importancia de realizar estudios video-EEG poligráficos de vigilia y sueño para control evolutivo en estos pacientes.

**Nistagmo Ictal: Importancia de la Exploración Clínica Ante la Presencia de Anomalías Electroencefalográficas**

Laura Menendez Rúa^1^, Raúl Armas Zurita^1^, Cielo González Mendoza^1^, Fadi Hallal Peche^1^, Mariano Aguilera Vergara^1^, David Gil Ruiz^1^, Goran Josic^1^

^1^Hospital Central de la Defensa Gómez Ulla, Madrid, España

**Introducción y objetivos**: La exploración clínica durante el electroencefalograma (EEG) es fundamental para poder discernir la semiología asociada a las anomalías en el registro. El nistagmo ictal es poco frecuente y puede pasar desapercibido. Son movimientos sacádicos, generalmente horizontales y la descripción de la dirección se basa en la fase rápida. En la mayoría de las ocasiones se asocia a un daño estructural subyacente. **Material y métodos**: Paciente de 76 años que ingresa por alteración fluctuante del nivel de consciencia. Entre sus antecedentes destaca el diagnóstico de un nódulo pulmonar sospechoso de malignidad diagnosticado en 2018, tratado con radioterapia y con datos de progresión posterior. El paciente rechazó, más pruebas diagnósticas y terapéuticas. **Resultados**: A las 24 horas del ingreso, se realiza un EEG donde se observan signos de afectación cortical difusa de grado moderado, se registraron 5 crisis epilépticas de hasta 72 s en 22 min. Electroencefalográficamente se observa una actividad epileptiforme que inicia en la región parieto-temporal izquierda, con posterior propagación contralateral. Semiológicamente, se inician con versión ocular y cefálica derecha, sonidos guturales y en alguna ocasión de manera sutil postura tónica de extremidades derechas. Durante las crisis no responde a estímulos, mientras que durante el intercrítico si responde, aunque permanece desorientado. Tras el diagnóstico de estatus epiléptico, se inicia tratamiento con antiepilépticos. Se realiza un control a las 24 h donde presenta 5 crisis de hasta 72 s en 30 min, eléctricamente similares a las previas, semiológicamente desaparece la postura tónica, permanece la desviación ocular hacia la derecha y aparece un nistagmo con fase rápida hacia la derecha. El TAC mostró una lesión metastásica occipital izquierda. **Discusión/Conclusiones**: El nistagmo es un signo que suele pasar desapercibido, pero con valor lateralizador. Aunque este ha sido ampliamente debatido, en la mayoría de los casos, la fase rápida del mismo es contralateral a la descarga epileptiforme, como en el caso descrito.

**Nuestra Experiencia con Hallazgos Incidentales de Actividad Epileptiforme en Registros Polisomnográficos Infantiles**

Carla Andrea Artacho Pérez^1^, Andrea Victoria Arciniegas Villanueva^1^, Emilio González García^1^, Maria José Ortiz Muñoz^1^, Jeimmy Mabel Pinzón Martínez^1^, Araceli Isabel Soucase García^1^, Ángela María Suero Cubilete^1^

^1^Hospital de Manises, Valencia, España

**Introducción y objetivos**: Los trastornos del sueño y la actividad epileptiforme (AE) durante el sueño presentan una alta asociación en la epilepsia infantil, con consecuencias clínicas y fisiopatológicas en esta población. El diagnostico diferencial es extenso, se ha demostrado que la Polisomnografía (PSG) estándar es una prueba diagnóstica sensible y específica que ayuda a identificar características clínicas clave ayudando a esclarecer las diferencias entre AE nocturna y trastornos del sueño. En algunos pacientes epilépticos la AE se incrementa en las fases del sueño NREM, además de alteraciones del desarrollo cognitivo y del aprendizaje. Pacientes asintomáticos pueden cursar con AE durante el sueño, en algunos casos como hallazgos incidentales en PSG solicitadas por otros motivos. Hasta un 45% de epilepsias pueden llegar a ser puramente nocturnas y su identificación es clave para el adecuado desarrollo. Presentamos nuestra casuística en estudios PSG pediátricos. **Material y métodos**: Revisión retrospectiva de los estudios de PSG pediátricos (0–15 años) realizados del 2022 al 2024. De 214 registros, 211 se remitieron por otorrinolaringología (ORL) y 3 por neuropediatría. En ellos identificamos los registros anormales (con AE). Determinamos sensibilidad (S), especificidad (E), valor predictivo positivo (VPP) y valor predictivo negativo (VPN) para evaluar la eficacia diagnóstica y la confianza de la PSG pediátrica en nuestro hospital. **Resultados**: De los 211 registros de PSG pediátrica derivados desde ORL se encontraron 7 pacientes con PSG con AE intercrítica, el 3,3%. En el 43% se halló punta onda continua durante el sueño (POCS), en el 28,6% puntas centro temporales y en el otro 28,6% AE indeterminada. La S fue de un 67%, la E de un 97% (VPP del 22%, VPN del 99%). **Discusión/Conclusiones**: La PSG en nuestra población tiene una especificidad de hallazgos de AE elevada, corroborada con electroencefalograma (EEG) posteriores, favoreciendo un diagnóstico precoz. Confirmamos que la PSG pediátrica es una prueba sensible con un elevado VPN.

**Evolución Clínica y Electroencefalográfica del Síndrome de Rett: Descripción de un Caso**

Ángela María Suero Cubilete^1^, Andrea Victoria Arciniegas Villanueva^1^, Emilio González García^1^, Ortiz Muñoz María José^1^, Jeimmy Mabel Pinzón Martínez^1^, Araceli Isabel Soucase García^1^, Carla Andrea Artacho Pérez^1^

^1^Hospital de Manises, Valencia, España

**Introducción y objetivos**: El síndrome de Rett es un trastorno del neurodesarrollo que afecta al sexo femenino, causado por variantes patogénicas del gen *MECP2*. En el 95% de los casos es variante típica, que presenta: aparición de retraso mental, cambios conductuales, pérdida del lenguaje, estereotipias de las manos, apraxia de la marcha, trastornos del sueño, alteraciones de la respiración y, de forma frecuente, crisis epilépticas. Hangberg y Wit-Engerstrism sugieren la evolución del electroencefalograma (EEG) en 4 estadíos. Presentamos el caso de una paciente adulta diagnosticada de síndrome de Rett de inicio temprano con hallazgos en el EEG de estadío 2.** Material y métodos**: Presentamos una paciente de 40 años, con discapacidad del 78%, en seguimiento desde el 2013 por el servicio de neurología del Hospital de Manises. Clínicamente ha tenido episodios de apnea, escoliosis (corsé hasta los 28 años), ausencia de control de esfínteres, estereotipias de lavado de manos, disfagia, retropiés en valgo de predominio izquierdo, ausencia de lenguaje y sin problemas de conducta. Actualmente, además, de rasgos autistas, movimientos estereotipados de las manos de forma más general, camina de forma cautelosa, acatisia y sin alteración del sueño, clínicamente en fase pseudoestacionaria 3. Controlada con FAES. EEG se objetiva Lentificación difusa de la actividad bioeléctrica cerebral y ritmos rápidos/ondas agudas en regiones centrales, mientras la PSG es normal lo que corresponde a un estadio EEG 2. **Resultados**: En el EEG la evolución ha sido mucho más lenta, preservando la arquitectura del sueño y correspondiéndose a un estadío 2 a pesar de la edad y que habitualmente lo que conocemos es que las anormalidades del EEG van acompañadas del deterioro clínico de forma paralela y progresiva. **Discusión/Conclusiones**: El Síndrome de Rett es una enfermedad degenerativa, progresiva y casi exclusivamente de mujeres. En nuestra paciente si bien la clínica es pseudoestacionaria fase 3 (consenso del 2001 Percy y cols.) en relación al EEG que está en Fase 2 siendo más lenta y conservada.

**Indicaciones de EEG Urgente Tras una Primera Crisis**

Pedro García Frutos^1^, Luis Santoveña González^1^, Alejandro Soriano García^1^, Maruvic Boruska Ramírez Arvelo^1^, Gianclaudio Dal Boni Gómez^1^, Sebastián Balay D’Agosto^1^, Jesús González Rato^1^

^1^Hospital Universitario Central de Asturias, Oviedo, España

**Introducción y objetivos**: Introducción: la realización de un video electroencefalograma (vEEG) tras una primera crisis, ya sea sintomática aguda o no provocada, sigue presentando ciertas dudas en cuanto al momento de su realización, así como a la conducta terapéutica a seguir en función de los hallazgos. **Objetivos**: conocer las principales etiologías de las primeras crisis epilépticas en población neonatal, pediátrica y adulta; y, por otro lado: esclarecer qué es lo que recomienda la evidencia en cuanto al momento óptimo para realizar un vEEG tras una primera crisis, actitud terapéutica en cuanto a los hallazgos del vEEG y rentabilidad de la monitorización vEEG para modificar la actitud terapéutica a medio-largo plazo. **Material y métodos**: Metodología: búsqueda en Pubmed, Embase y Dynamed (revisión sistemática de los últimos 10 años, sin excluir estudios previos que aporten información relevante), con revisión bibliográfica de 19 artículos de población neonatal y 22 artículos de pediátrica y adulta, sobre la realización de estudios vEEG de forma urgente. **Resultados**: Las crisis neonatales son una de las principales emergencias neurológicas en esta edad, presentando alta mortalidad y morbilidad; la realización de un vEEG es el gold estándar para su diagnóstico y manejo terapéutico precoz, además, en muchos casos se expresan sólo como crisis sutiles o electrográficas, por lo que la monitorización vEEG se vuelve imprescindible. Las crisis epilépticas en edad pediátrica y adulta pueden ser sintomáticas agudas, consecuencia de una lesión cerebral reciente, que puede desembocar en una epilepsia de causa estructural. Provocadas, debidas a alteraciones metabólicas, consumo de drogas o intoxicaciones agudas, que disminuyen el umbral convulsivo. Y no provocadas, en las que no hallamos ninguna causa que justifique su precipitación. **Discusión/Conclusiones**: Es importante conocer las condiciones en las que la realización de un vEEG urgente (primeras 24 horas) puede ser decisiva para la orientación diagnóstica y terapéutica del paciente.

**Encefalitis Autoinmune Asociada a Anticuerpos anti-LGI1: Caracterización Electroclínica en una Serie de Casos**

Elena Escario Méndez^1^, Luigi Pietro Carlo Unda^1^, Rolando Agudo Herrera^1^, Erika Herráez Sánchez^1^

^1^Hospital Universitario Rey Juan Carlos, Madrid, España

**Introducción y objetivos**: La encefalitis por anticuerpos antiLGI1 es una entidad de inicio subagudo caracterizada clínicamente por confusión, alteraciones de la memoria y aparición de crisis epilépticas, siendo típicas las crisis distónicas faciobraquiales. El electroencefalograma (EEG) se caracteriza por la aparición de actividad intercrítica lenta o epileptiforme, típicamente en región temporal, y en los escasos registros ictales descritos se ha observado la presencia de una onda muy lenta frontal contralateral o actividad electrodecremental previa a las crisis, si bien existen pocos reportes y con importante variabilidad en los hallazgos. El objetivo de este trabajo es describir los hallazgos electroclínicos en una serie de 3 casos. **Material y métodos**: Se realiza un estudio retrospectivo descriptivo de 3 casos clínicos y revisión de la literatura. **Resultados**: Se estudian 3 pacientes, dos varones y una mujer, de entre 52 y 77 años, con clínica inicial de síndrome confusional, y crisis focales motoras en dos casos. 1 caso mostró gliosis temporal medial derecha en neuroimagen. Se realizaron al menos 3 EEG por paciente, observándose actividad lenta temporal en los 3 casos, anomalías epileptiformes temporales en 1 caso, y registrándose 4 episodios compatibles con crisis faciobraquiales en uno de ellos, observándose una atenuación previa en uno de los episodios. Todos los pacientes mostraron anticuerpos antiLGI1 positivos en LCR y se inició tratamiento precoz. **Discusión/Conclusiones**: La encefalitis con anticuerpos antiLGI 1 es una entidad grave y potencialmente reversible, por lo que es necesario su correcta identificación para aplicar un tratamiento dirigido. Dado el reducido número de pacientes descrito en la literatura y la heterogeneidad de los hallazgos electroclínicos, el reporte de esta serie puede contribuir a ampliar el conocimiento de esta patología y facilitar así su reconocimiento precoz para evitar demora en el diagnóstico y permitir una adecuada orientación terapéutica.

**Epilepsia de la Ínsula: Un Reto Diagnóstico**

Rosa Altagracia Beriguete Alcántara^1^, Erika Herráez Sánchez^1^, Javier Martínez Poles^1^, Rolando Agudo Herrera^1^

^1^Hospital Universitario Rey Juan Carlos (HURJC), Madrid, España

**Introducción y objetivos**: La epilepsia de la ínsula es uno de los tipos más complejos de diagnosticar debido a la heterogeneidad de sus manifestaciones clínicas, siendo catalogada como la gran simuladora. Debido a su amplio abanico de funciones y extensa red de conexiones, las crisis pueden presentar manifestaciones viscerales, sensoriales, conductas motoras complejas y síntomas autonómicos, siendo erróneamente diagnosticada. El objetivo de este caso es ilustrar la semiología insular y la dificultad del diagnóstico de este tipo de crisis. **Material y métodos**: Se describe un caso clínico estudiado en nuestro laboratorio de monitorización de electroencefalograma (EEG). **Resultados**: Mujer de 29 años con epilepsia desde los 14 años, con crisis focales con o sin alteración del nivel de conciencia, en el pasado con evolución tónico-clónica bilateral. Actualmente con episodios de náuseas y malestar gástrico, con sospecha de eventos no epilépticos; se realiza EEG prolongado de 5 días para diagnóstico diferencial. Al inicio, presenta estos episodios sin cambios EEG, observándose cambio en la expresión facial, incapacidad para la articulación del habla e hipersalivación, con comprensión conservada; al reducir la medicación anticrisis, se observan cambios EEG en región temporal basal derecha durante los episodios, uno de ellos evoluciona a tónico-clónico bilateral. El estudio semiológico y EEG de los eventos, sugiere un origen insular derecho. Resonancia magnética nuclear (RMNc) no lesional. La tomografía por emisión de positrones (PET-TC) sugiere hipometabolismo neocortical temporal derecho. **Discusión/Conclusiones**: Las crisis epilépticas de origen insular presenta manifestaciones clínicas muy variadas, confundibles con eventos no epilépticos, su expresión eléctrica puede observarse en diversas topografías, dificultando su diagnóstico. Un minucioso análisis semiológico y del EEG es necesario para realizar el diagnóstico y una correcta aproximación a la zona epileptógena, que deberá confirmarse con estudios más complejos como estereo-encefalograma (S-EEG).

**Estado Epiléptico no Convulsivo Secundario a Tratamiento Quimioterápico con Buena Respuesta a Benzodiacepinas**

Joseba Soto Ibáñez^1^, Elisabet Madrigal Lkhou^1^, Alexandra Reinoso Aguirre^1^, Jennifer Paola Cantarero Durón^1^, Diego Francisco Peña Olaya^1^, Alina Havrylenko Vynogradnyk^1^, Fernando Vázquez Sánchez^1^, Fernando Vázquez Sánchez^1^

^1^Hospital Universitario de Burgos, Burgos, España

**Introducción y objetivos**: A propósito de estudiar el caso de una mujer de 44 años con estatus epiléptico no convulsivo (EENC) con buena respuesta a la administración de benzodiacepinas (BDZ) en contexto de tratamiento quimioterápico (QT) por un sarcoma de alto grado de localización peritiroidea, nos proponemos revisar dicho cuadro clínico, las posibles relaciones existentes entre un EENC y la administración de un tratamiento QT y los hallazgos electroencefalográficos presentes durante su ingreso en planta de hospitalización. Se trata de una mujer con antecedentes de Neurofibromatosis tipo I, mioma uterino, nódulo mamario en seguimiento e hipotiroidsimo tras tiroidectomía por tumoración maligna de la vaina del nervio periférico. Tras la administración de QT, la paciente presentó una crisis tónico-clónica generalizada sin datos de lesión aguda en tomografía axial computarizada (TAC) craneal sin contraste y con actividad epiléptica generalizada registrada en video-electroencefalograma (vEEG), se pautó Keppra 500 mg/12 horas y Lamictal 100 mg/12 h. Tras la segunda sesión de QT, la paciente presentó 3 nuevas crisis tónico clónicas seguidas con buena evolución tras la toma de Keppra 100 mg. Al mes fue hospitalizada por nueva crisis no presenciada. El vEEG objetivó un trazado compatible con un estado epiléptico generalizado con clara mejoría tras la introducción de BDZ. **Material y métodos**: TAC craneal, resonancia magnética cerebral y los análisis del líquido cefalorraquídeo no resultaron significativos. **Resultados**: El vEEG aportó datos respecto al EENC, la resolución tras la administración de diazepam y la mejoría en controles evolutivos hasta una mínima expresión de alteraciones epilépticas. **Discusión/Conclusiones**: El estatus epiléptico es un cuadro originado por el fallo de los mecanismos responsables de la terminación de las convulsiones o el inicio de los mecanismos que conducen a convulsiones anormalmente prolongadas. El cáncer y el tratamiento QT son dos situaciones que pueden influir en la aparición de eventos críticos.

**EstereoEEG y la Estimulación Cortical con Frecuencias Intermedias Para Mapeo del Lenguaje: Exposición de un Caso**

Inmaculada Concepció Lozano Cuadra^1^, Aileen McGonigal^2^

^1^Hospital Universitario Virgen del Rocío, Sevilla, Sevilla, España

^2^Mater Hospital, Australia

**Introducción y objetivos**: La técnica de SEEG desempeña un papel fundamental en el estudio prequirúrgico de pacientes con epilepsia farmacorresistente. Su principal función radica en identificar la zona epileptogénica y la localización de áreas elocuentes, garantizando así la planificacion de un tratamiento quirúrgico individualizado y preciso, que valore la relación riesgo-beneficio para cada paciente. Una de las áreas más desafiantes de localizar es la del lenguaje, debido a su variabilidad individual y la falta de consenso sobre su localización precisa. En un estudio reciente, se exploró la estimulación a frecuencias intermedias (IFS) para el mapeo del lenguaje en pacientes con SEEG. Se encontró que estas frecuencias eran igualmente efectivas que la estimulación a 50 Hz (HFS), pero con menos descargas post-estimulación y posibilidad de mayor duración de estímulo, lo que permitía una exploración más detallada y segura. **Material y métodos**: Caso: Mujer 36 años afecta de Epilepsia temporal antero-mesial izquierda criptogénica farmacorresistente. Episodios de sensación de calor, sudoración, desconexión del medio, automatismos orales y postura distónica de la mano derecha, con progresión a TC. Tecnica: SEEG (16 electrodos intracorticales), estimulación cortical a 1, 6, 9 (15 s, 4 mA) y 50 Hz (5 s, 4 mA). **Resultados**: Con este estudio pudimos definir la zona epileptogénica (hipocampo i anterior > hipocampo i posterior > amígdala i > polo temporal i) y la zona irritativa (amígdala i > hipocampo i > polo temporal i). Además, concluimos que las áreas del lenguaje no estaban implicadas en la zona epileptogénica primaria, sino que forman parte de un circuito de propagación posterior. **Discusión/Conclusiones**: Gracias a la tecnica SEEG pudimos determinar que las areas del lenguaje no formaban parte de la zona epileptogenica primaria y pudimos ofrecerle tratamiento a la paciente sin causar deficit secundarios. En nuestro caso utilizamos IFS (6–12 Hz) durante la corticoestimulacion para el mapeo del lenguaje y obtuvimos resultados similares a los descritos en la literatura.

**Seguimiento Vídeo-EEG en Paciente con Status Epiléptico Súperrefractario**

Raquel López-Carvajal Hijosa^1^, M. Rodríguez Jiménez^1^, M. Picornell Darder^1^, G. Naranjo Heredia^1^

^1^Hospital Universitario de Móstoles, Madrid, España

**Introducción y objetivos**: El estatus epiléptico es una urgencia neurológica que consiste en la recurrencia de crisis epilépticas sin recuperación de la consciencia entre ellas o la aparición de una crisis epiléptica continua durante más de 30 minutos. Hay dos tipos; estatus epiléptico convulsivo (EEGC) y estatus epiléptico no convulsivo (EENC). Ambos se diferencian en la clínica, electroencefalograma (EEG), mortalidad y complicaciones. Describir las anomalías epileptiformes en los estudios video-EEG según la clasificación de status epiléptico. **Material y métodos**: Varón de 50 años con antecedentes de encefalopatía anóxica perinatal y crisis epilépticas en tratamiento con lamotrigina y oxcarbamacepina, acude a Urgencias por crisis tónico-clónicas, en contexto de infección respiratoria y que evoluciona a un status epiléptico súperrefractario. **Resultados**: Se realizan varios video-EEG poligráficos evolutivos, en el primero se registran anomalías multifocales epileptiformes intercríticas (paroxismos P-O) en áreas F-Ts/T-P-Os de predominio en hemisferio izquierdo. Posteriormente, a partir del segundo video-EEG y los consecutivos, se observa un status epiléptico convulsivo. **Discusión/Conclusiones**: Importancia de la realización de video-EEG poligráfico en el seguimiento evolutivo de pacientes con status epiléptico refractario.

**Entre Realidades Distorsionadas: El Síndrome de Alicia en el País de las Maravillas y su Correlato Neurofisiológico**

Luigi Carlo Unda^1^, Erika Herráez Sánchez^1^, Elena Escario Méndez^1^, Rosa Altagracia Beriguete Alcántara^1^

^1^Hospital Universitario Rey Juan Carlos, Madrid, España

**Introducción y objetivos**: El síndrome de Alicia en el País de las Maravillas es una entidad clínica caracterizada por breves episodios de ilusiones visuales con distorsión del tamaño, forma o distancia de los objetos, que a pesar de su curso generalmente benigno suele causar gran alarma a quienes lo padecen. Puede estar asociado a diversas condiciones patológicas o presentarse como un cuadro idiopático y autolimitado, sobre todo en la edad pediátrica, y obliga a realizar un diagnóstico diferencial con otras patologías que precisen un manejo terapéutico distinto, como la epilepsia, lesiones del sistema nervioso central o trastornos psiquiátricos. El objetivo de este estudio es describir un caso clínico ilustrativo. **Material y métodos**: Se presenta el caso de un niño de 9 años sin antecedentes relevantes, que experimenta episodios autolimitados de minutos de duración consistentes en distorsión visual tipo micropsia, y que es remitido a nuestro laboratorio para realización de video-electroencefalograma (v-EEG). **Resultados**: El v-EEG muestra una actividad de fondo normal en vigilia, con ritmo posterior dominante en rango alfa a 10 Hz. Durante la prueba, se registran dos episodios de duración variable (20 segundos–7 minutos), clínicamente similares a los habituales del paciente, y caracterizados eléctricamente por la aparición con ojos abiertos de una actividad theta a 6–7 Hz en la región posterior bilateral, sin patrón evolutivo, y la reaparición del ritmo alfa al cerrar los ojos o al remitir la clínica. **Discusión/Conclusiones**: Existen pocos casos publicados de esta entidad en la literatura, y menos aún con correlato electroencefalográfico. La presencia de actividad lenta en la región occipital bilateral durante los episodios sugiere disfunción cortical en dicho territorio, siendo necesarios estudios dirigidos para una mejor categorización fisiopatológica. La identificación de este cuadro clínico y el conocimiento de sus características electrofisiológicas es relevante para establecer un diagnóstico adecuado y un manejo posterior apropiado.

**Pronóstico de los Pacientes con Patrón Alternante Cíclico de la Encefalopatía**

Patricia Santamaria Brea^1^

^1^Hospital Universitario de Bellvitge, Barcelona, España

**Introducción y objetivos**: El patrón alternante cíclico de la encefalopatía (CAPE) introducido en la terminología estandarizada del paciente critico en el año 2021, se define como la alternancia de dos patrones con una duración mínima de 10s cada uno. Actualmente existen pocas correlaciones clínico-pronosticas con este patrón, nos proponemos describir variables demográficas, etiológicas y pronósticas en pacientes con CAPE. **Material y métodos**: Se recogen de forma retrospectiva 34 pacientes codificados como CAPE en la base de datos de un hospital terciario de adultos (abril 2022–abril 2024). Se registran las variables demográficas, clínicas y pronósticas utilizando la escala Cerebral Performance Categories (CPC). **Resultados**: Se identificaron 34 pacientes (20 hombres, 14 mujeres) edad media de 65,5 años (SD 15,9; rango 27–91). Motivos de ingreso: crisis epilépticas 21%, hemorragia de SNC (HSA 21%, hematomas intraparenquimatosos 12%), TCE 12%, encefalopatía anóxica 9%, ictus isquémicos 6%, infecciones del SNC 6%, shock séptico 6%, hiponatrèmia 6% y otros 3%. Situación clínica al alta: sin déficit neurológico (CPC 1) 26%, incapacidad leve (CPC 2) 41%, incapacidad moderado/grave (CPC 3) 12%, coma vegetativo (CPC 4) 3%, muerte (CPC 5) 18%. Independientemente del motivo de ingreso el 32% de pacientes realizaron crisis epilépticas previas al registro electroencefalograma (EEG) y de estos un 18% repitieron crisis epilépticas posteriormente al registro. De los 34 pacientes un 74% estaban en tratamiento con fármacos anticrisis (FACs) antes del registro EEG con patrón CAPE, de los cuales a un 52% se les disminuyeron o retiraron los FACs antes del alta hospitalaria. **Discusión/Conclusiones**: El patrón alternante cíclico de la encefalopatía está presente en multitud de etiologías, y conlleva un pronóstico favorable en un 68% de los pacientes (considerando buen pronóstico puntaciones de 1–2 en la escala CPC). Tras los resultados encontrados se evidencia la importancia de identificar este patrón para un buen ajuste terapéutico posterior y evitar así una escalada de FACs.

**Revisión de los Patrones de EEG en el Síndrome de Angelman y la Importancia de su Hallazgo Para el Diagnóstico Precoz**

Sebastián Sagula^1^, Neus González Arnau^1^, Lara Martín Muñoz^1^, Marta Luna Mateos^1^, Dolors Casellas Vidal^1^

^1^Hospital Universitario de Gerona Doctor Josep Trueta, Girona, España

**Introducción y objetivos**: El síndrome de Angelman (SA) es un trastorno neuroconductual raro causado por anomalías del gen *UBE3A* materno o del cromosoma que lo contiene 15q11-q13. El fenotipo completo se observa entre los 3 y 7 años de vida: retraso mental severo, ausencia del desarrollo del lenguaje, apariencia feliz, paroxismos de risa inmotivada, fenotipo peculiar, trastornos del movimiento y epilepsia. El electroencefalograma (EEG) puede mostrar alteraciones características a partir de los 4 meses de vida. Las pruebas genéticas confirman el diagnóstico. Presentamos cuatro casos de niños con retraso psicomotor y EEG alterado que posteriormente tuvieron confirmación genética de SA. **Material y métodos**: Realizamos EEG a cuatro pacientes de 5, 16, 17 y 24 meses de vida, con retraso psicomotor y que presentaron primer episodio sugestivo de crisis comicial. Los resultados se compararon con los tres patrones característicos de SA descritos por Boyd* et al*. (1988): I, actividad theta rítmica prolongada a 4–6 Hz que no se elimina con el cierre palpebral. II, actividad rítmica delta generalizada a 2–3 Hz, en regiones anteriores. III, actividad delta rítmica de morfología contorneada >200 µV con o sin puntas/ondas agudas, en regiones posteriores y facilitadas con el cierre palpebral.** Resultados**: Los 4 pacientes presentaron trazado característico del patrón I y II descrito por *Boyd*. Fueron informados como sugestivos de SA y se les solicitó a todos una prueba genéticaconfirmando la deleción del cromosoma materno 15q. **Discusión/Conclusiones**: Los tres patrones de los EEG deberían utilizarse como red flags para aumentar significativamente el índice de sospecha del diagnóstico de SA, especialmente en etapas tempranas de la vida antes de que las características fenotípicas del síndrome sean evidentes. Recomendar al pediatra solicitar prueba de biología molecular siempre que el EEG muestre cualquiera de los tres patrones, ya sea aislado o combinado. Consejo genético oportuno ante la presencia de mutaciones con alto riesgo de recurrencia.

**Estadíos Clínicos y Cambios Electroencefalográficos en 5 Pacientes con Enfermedad de Creutzfeld-Jacob**

Alejandro Melcon Villalibre^1^, Octavio Ramírez Hernández^1^, Paloma Balugo Bengoechea^1^, Adela Fraile Pereda^1^, Lorena Iglesias Alonso^1^, Guillermo Ceide García^1^, Gianna Doménika Bozano Subía^1^

^1^Hospital Clínico San Carlos, Madrid, España

**Introducción y objetivos**: La enfermedad de Creutzfeld-Jacob (ECJ) una enfermedad por priones que causa una demencia rápidamente progresiva con síntomas psiquiátricos/conductuales, cerebelosos, piramidales, extrapiramidales y alteraciones visuales. El diagnóstico se basa en la clínica, la resonancia magnética y biomarcadores en el líquido cefalorraquideo (proteína 14-3-3). Presentamos una serie de casos dónde además se analiza el correlato electroencefalográfico (EEG) según las diferentes etapas de la enfermedad y estudio de la asociación entre los patrones EEG y los estadios clínicos de la ECJ. **Material y métodos**: Se han analizado los datos clínicos de 5 pacientes con ECJ entre los 49–85 años, estableciendo las fases de la enfermedad en base a signos y síntomas clínicos: el estadio I definido por signos y síntomas neurológicos menores; el estadio II por el marcado deterioro de las funciones superiores, afectación sensitivo-motora, cerebelosa, tronco-encefálica, piramidal/extrapiramidal y demencia leve; y el estadio III por presencia de mioclonías y demencia moderada-grave, mutismo acinético y posterior muerte. **Resultados**: Se obtuvieron: 1 paciente en estadio I, 1 paciente en estadio II y 3 en estadio III. El enlentecimiento de fondo en el rango theta/delta fue una característica independiente al estadio clínico, pero en el en estadio I/II se objetivó un marcado enlentecimiento asimétrico delta rítmico intermitente frontal y temporal respectivamente. Los pacientes en estadio III tuvieron anomalías adicionales en el EEG como elementos periódicos, ondas trifásicas y agudas. **Discusión/Conclusiones**: El enlentecimiento de fondo es la característica principal siendo inicialmente unilateral y posteriormente generalizado y, además, en el estadio III se observan complejos periódicos. Es importante tener presente la variabilidad del EEG en este tipo de pacientes y ser capaces de reconocer los patrones según el estadio de la enfermedad (sobre todo en fases precoces) dada la baja prevalencia de esta enfermedad y la repercusión pronóstica en estos pacientes.

**¿Es una Crisis? La Utilidad de la Monitorización vídeo-EEG Prolongada**

Gonzalo Ferrer Ugidos^1^, Christian Hernández Aranda^1^, Margely Abete Rivas^1^, África Bueno García^1^, María Martín Carretero^1^, José Miguel Leon Alonso^1^, Sara Muniesa Lacasa^1^

^1^Hospital Clínico Universitario de Valladolid, Valladolid, España

**Introducción y objetivos**: Establecer la utilidad de la monitorización vídeo-electroencefalograma (EEG) prolongada en el diagnóstico diferencial entre una crisis epiléptica y un evento paroxístico no epiléptico. **Material y métodos**: Se realiza una revisión de 3 pacientes, dos mujeres y un hombre, que presentaron eventos paroxísticos cuya descripción clínica era de dudosa clasificación etiológica. Se les realizó una monitorización vídeo-EEG prolongada continua, durante 5 días consecutivos en cada uno de los casos, colocando electrodos externos según el sistema 10–20. **Resultados**: La primera paciente presentó varios episodios de 3–5 minutos, consistentes en versión ocular, hiperventilación y movimientos de extremidad superior derecha de características variables, sin reproducción similar en el tiempo, sin correlato patológico en el trazado EEG. El segundo paciente presentó dos episodios de características similares entre sí, de 90–75 segundos, comenzando con un despertar súbito, seguido de una rápida incorporación e intento de deambulación. Posteriormente, el paciente presentó una extensión distónica a nivel cervical con retrocolis, supra versión de la mirada y signo del puchero ictal. Esta extensión distónica del tronco condicionó una caída hacía atrás, quedando el paciente en una postura de hiperextensión tónica. En el EEG se registró patrón crítico durante los episodios. La tercera paciente refirió eventos de tipo despersonalización, preservando la consciencia, sin registro de correlato eléctrico patológico, si bien, se identificó una actividad epileptiforme interictal ya conocida en registros previos. **Discusión/Conclusiones**: El diagnóstico diferencial de los eventos paroxísticos es amplio y variado. Para realizar un correcto diagnóstico, es fundamental una detallada anamnesis y frecuentemente es necesaria la utilización de monitorización video-EEG prolongada, para asociar las manifestaciones clínicas con los hallazgos electroencefalográficos, y establecer la etiología y tratamiento oportuno si lo necesita.

**Hallazgos Electroencefalográficos en la Lipofuscinosis Ceroide Neuronal (CLN). A Propósito de 2 Casos en Edad Infantil**

Patricia Palta Vázquez^1^, Monica Vicente Rasoamalala^1^, Miquel Raspall Chaure^1^

^1^Hospital Universitari de la Vall d’Hebron, Barcelona, España

**Introducción y objetivos**: La lipofuscinosis neuronal ceroide (CLN) es una enfermedad neurodegenerativa autosómica recesiva. Aparece en edad infantil y se caracteriza por la presencia de epilepsia y la regresión motriz, del lenguaje y déficit visual. El diagnóstico final es genético, si bien existen hallazgos característicos en la neuroimagen y en las pruebas neurofisiológicas. El objetivo del estudio es presentar el fenotipo clínico y los hallazgos electroencefalográficos (EEG) a lo largo de 4 años de seguimiento. Los EEG fueron realizados por el equipo EEG pediátrico de Neurofisiología Clínica del HUVH. **Material y métodos**: Se presenta un estudio descriptivo de 2 casos clínicos con diagnóstico de CLN, en seguimiento por Neuropediatría del Hospital Universitari Vall d’Hebron (HUVH). **Resultados**: 2 pacientes de sexo femenino, de 8 y 9 años, diagnosticadas de CLN. Caso 1: Niña de 8 años que a los 5 inicia alteración de lenguaje, atención y habilidades motrices. Posteriormente, episodios de desconexión. Presenta atrofia cerebelar en RM. El EEG muestra actividad epileptiforme en regiones posteriores bilaterales sobre una desesestructuración progresiva de la actividad cerebral y aparición de fotosensibillidad. Genética positiva para 2 variantes en trans para CLN5. Caso 2: Niña de 9 años con trastorno del aprendizaje y episodios de amaurosis, desviación óculo-cefálica y caída cefálica desde los 5 años. EEG con abundante actividad epileptiforme multifocal posterior. Genética positiva para una variante en heterocigosis para CLN8. **Discusión/Conclusiones**: La CLN debe sospecharse ante pacientes pediátricos con epilepsia, alteraciones visuales y un trastorno cognitivo-conductual progresivo. La realización de EEGs seriados pueden sustentar la sospecha diagnóstica cuando se documenten anomalías epileptiformes, fotosensibilidad y enlentecimiento y desestructuración progresivos de la actividad cerebral de base, permitiendo una mejor caracterización electroclínica de los diferentes subtipos de CLN.

**Patrón “Delta-brush”: Un Indicador Electroencefalográfico en el Video EEG de Superficie de Displasias Corticales Focales**

Luis Gabriel Burgos Bustamante^1^, Julio Ignacio Prieto Montalvo^1^, Laura Verna Fierro^1^, Pedro José Melgarejo Otárola^1^

^1^Hospital General Universitario Gregorio Marañón, Madrid, España

**Introducción y objetivos**: El patrón electroencefalográfico conocido como “delta brush” ha sido tradicionalmente asociado a encefalitis autoinmune, especialmente la anti receptor NMDA; no obstante, ha sido descrito más infrecuentemente en otras patologías como la epilepsia temporal mesial o en pacientes críticos con encefalopatías metabólicas. En registros con electrodos intracraneales puede ser habitualmente encontrado en pacientes con displasia cortical focal. **Material y métodos**: Se presenta el caso de una mujer de 21 años con múltiples antecedentes congénitos, entre los más relevantes: ductus arterioso y comunicación inter-auriculo-ventricular, corregidos quirúrgicamente a los 3 meses de edad; además de escoliosis, espina bífida y vejiga neurogénica con hidronefrosis izquierda grado IV. No historia previa de epilepsia. El motivo del actual ingreso hospitalario fue para una reintervención programada de valvuloplastia tricúspidea. Durante el postoperatorio, al despertar y ser extubada, la paciente cursó con tres eventos críticos de características similares: inicialmente con postura tónica del miembro superior izquierdo y generalización posterior. Los dos primeros cesaron espontáneamente, mientras que el tercero fue controlado con Clonazepam IV. Se realizó un videoEEG (vEEG) con electrodos de superficie al día siguiente. **Resultados**: El vEEG observó una actividad de fondo conservada, sobre el que se registraron salvas de actividad delta bilateral con predominio hemisférico derecho y ocasionales anomalías epileptiformes fronto-centrales del mismo lado. Se pudieron identificar grafoelementos tipo “delta brush” en esta misma localización, sugiriéndose la posibilidad de una displasia cortical focal. La resonancia magnética de 3 teslas posterior confirmó la existencia de dicha malformación. **Discusión/Conclusiones**: La presencia de grafoelementos tipo delta brush en el EEG de superficie en pacientes con crisis focales, aunque menos frecuentemente descrito que en los registros intracraneales, puede ser un hallazgo sugerente de displasias corticales focales.

**Estatus Epiléptico Descripción Clínico Electroencefalográfica, Etiológica y Pronostica de una Serie de Casos**

Christian Javier Hernández Aranda^1^, Margely Abete Rivas^1^, María Martín Carretero^1^, José Miguel León Alonso Cortes^1^, África Bueno García^1^, Gonzalo Ferrer Ugidos^1^, Sara Muniesa Lacasa^1^

^1^Hospital Clínico Universitario de Valladolid, Valladolid, España

**Introducción y objetivos**: Definir los aspectos generales, consideraciones clínicas y electroencefalográficas, así como el pronóstico en un grupo de pacientes con estatus epilépticos de diferentes etiologías. **Material y métodos**: Se llevo a cabo un análisis retrospectivo de 5 pacientes con sospecha clínica de estatus epiléptico, 40% mujeres y 60% hombres, con posterior confirmación diagnostica mediante video-electroencefalograma (V-EEG), a los cuales se les realizo un adecuado seguimiento clínico y electroencefalográfico, valorando la evolución y pronóstico de los pacientes. **Resultados**: Dos pacientes fueron ingresados por parada cardio respiratoria; el primer caso presento un despertar patológico, el segundo sintomatología discreta sospechosa de estatus no convulsivo, tercer paciente fue diagnosticado de encefalitis herpética, el cuarto paciente ingresó por un hematoma subdural, estos 2 últimos casos presentaron mioclonías faciales; un quinto paciente con diagnóstico de epilepsia generalizada en relación PCI y mala adherencia al tratamiento FAC, presentando varios episodios de crisis tónico clónicas generalizadas. Por la alta sospecha clínica que presentaron los pacientes se realizó un V-EEG como prueba complementaria y diagnostica, concluyendo que cumplían criterios electroencefalográficos de estatus epiléptico (convulsivo/no convulsivo según el caso), colaborando a la implementación de un manejo terapeutico adecuado y posteriormente un seguimiento periódico mediante V-EEG según la evolución clínica del paciente, facilitando una impresión pronóstica oportuna; Los dos primeros pacientes tuvieron un mal pronóstico mientras que en 3 los últimos contaron con buena evolución. **Discusión/Conclusiones**: El estatus epiléptico es una emergencia médica neurológica que amerita una actuación rapida y sistematizada, en ocasiones la sospecha diagnostica puede ser de difícil deducción, por la variabilidad de su etiología y manifestaciones clínicas, es importante enfatizar el uso de electroencefalograma como herramienta fundamental de ayuda diagnóstica y seguimiento en estatus epileptico.

**Patología del Neurodesarrollo Relacionada con Mutaciones en el Gen SLC6A1 y Anomalías EEG, A Propósito De Dos Casos**

Julián Vázquez Lorenzo^1^, Sofía Ortigosa Gómez^1^, Carmen María Garnés Sánchez^1^, Sara Giménez Roca^1^, Pilar Martínez Martínez^1^, Víctor Hugo Rubio Suárez^1^, Reetika Baharani Baharani^1^

^1^Hospital Universitario Virgen De La Arrixaca, Murcia, España

**Introducción y objetivos**: La enfermedad ligada a mutaciones en SCL6A1 se caracteriza por retraso del neurodesarrollo y del lenguaje, trastornos del movimiento, manifestaciones psiquiátricas (TDAH, TEA) y epilepsia/alteraciones EEG. **Material y métodos**: Se describen los casos de dos pacientes de HUVA. **Resultados**: Dos niños de 4 y 7 años, con debut de la sintomatología a los 10 y 11 meses de edad. Ambos con mutación *de novo* en heterocigosis en el gen *SCL6A1*. El primer paciente debutó con crisis de ausencia y crisis mioclónicas/atónicas, cumpliendo criterios de epilepsia con crisis mioclónico-atónicas (EMA). Actualmente en tratamiento con VPA y LMT, libre de crisis. Presenta ataxia truncal y alteraciones del comportamiento. El segundo paciente presenta un TEA tipo 1 con afectación de la esfera comunicativa y discapacidad intelectual leve. Nunca ha presentado crisis clínicas. Ambos con pruebas de imagen normales. Electroencefalograma (EEG): En ambos se observan anomalías epileptiformes de distribución multifocal y ondas lentas, apareciendo de forma síncrona y asíncrona interáreas e interhemisferio, en en sueño NREM y en situaciones con efecto similar a hiperventilación, se sincronizan y aparecen como descargas generalizadas extensas (hasta 28 segundos de duración). **Discusión/Conclusiones**: La enfermedad relacionada con SCL61A origina epilepsia en el 85% de los casos. La alteración EEG es prácticamente universal. Como en nuestros casos, suelen observarse descargas generalizadas, compuestas por P-O a 2.5–3.5 Hz y ondas lentas, con o sin tener correlato clínico (ausencias atípicas o crisis mioclónicas/atónicas). No obstante, esta enfermedad puede únicamente ocasionar un trastorno del neurodesarrollo tipo TEA, como en el segundo paciente. En los pacientes con un patrón clínico consistente en TEA, retraso del lenguaje, trastornos del movimiento, epilepsia tipo ausencia y/o EMA debe pensarse en las mutaciones en heterocigosis SCL6A1 si no se ha llegado a un diagnóstico de certeza. Los hallazgos EEG no son específicos, pero en un contexto clínico adecuado pueden guiar el diagnóstico.

**Importancia de las Áreas de Lüders en la Evaluación Prequirúrgica en un Paciente con Epilepsia Refractaria**

Alejandro José Soriano García^1^, Luís Santoveña González^1^, Jesús Rodríguez Rato^1^, Noelia Rodríguez Rodríguez^1^, Maruvic Boruska Ramírez Arvelo^1^, Gianclaudio Dal Boni Gómez^1^, Consuelo Vallés Antuna^1^

^1^Hospital Universitario Central de Asturias, Oviedo, España

**Introducción y objetivos**: Las áreas de Lüders son un conjunto de zonas descritas por el neurólogo Hans Lüders para localizar los focos de origen en determinadas epilepsias. Su delimitación se basa en el uso de la clínica en relación a los hallazgos de varias pruebas diagnósticas y permite determinar el abordaje quirúrgico con mayor precisión dada la mínima zona de tejido epileptógeno a extirpar. Presentamos la utilidad de estás zonas en el abordaje quirúrgico de un paciente con epilepsia refractaria para conseguir un estado libre de crisis. **Material y métodos**: Varón de 21 años diagnosticado de epilepsia refractaria derivado a la unidad de referencia nacional por empeoramiento clínico de sus crisis habituales, las cuales inician con desviación cefálica y de la mirada hacia la izquierda con posterior hipertonía generalizada. Se realiza monitorización electroencefalograma (EEG) durante días para localizar la zona epileptógena a través de la delimitación de las áreas de Lüders, aplicando EEG cuantificado, identificando un probable foco epileptógeno profundo en región parieto-temporal derecha, se amplía el estudio mediante la colocación de siete electrodos profundos en la confluencia de dicha región. **Resultados**: La información obtenida permitió calcular con mayor exactitud las áreas de Lüders y un consecuente foco epileptógeno en la región temporal mesial derecha, estableciendo como diagnostico una epilepsia reticular temporal plus. Debido al difícil acceso se considera electrocoagulación a través de los electrodos profundos. En la monitorización posterior se constata una mejoría del trazado, con disminución significativa de la actividad epileptiforme interictal. **Discusión/Conclusiones**: Se pone de manifiesto la importancia del conocimiento de áreas de Lüders, tanto para el diagnóstico de la zona epileptógena, como para una mejor compresión de la fisiopatológica epiléptica. Además de poner en valor la necesidad de las unidades de video-EEG y la Neurofisiología en el manejo de las epilepsias refractarias.

**Tiempos de Atención a las Crisis Urgentes: Datos de un Registro Multicéntrico**

Jacint Sala Padró^1^, Elena Fonseca Herández^1^, Manuel Quintana^1^, Estevo Santamarina Pérez^1^, Misericorida Veciana de las Heras^1^, Mercè Falip Centellas^1^, Manuel Toledo Argany^1^

^1^Hospital Universitario de Bellvitge, Barcelona, España

**Introducción y objetivos**: En el tratamiento del estado epiléptico (EE) son vitales los tiempos de acceso a la atención, al tratamiento y al diagnóstico adecuados. Nuestro objetivo es revisar esta atención, comparando los tiempos de activación del Sistema de Emergencias Médicas (SEM), el tratamiento prehospitalario y el acceso al electroencefalograma (EEG). **Material y métodos**: Desde 2023, se ha completado un registro prospectivo en dos centros terciarios introduciendo todos los pacientes que consultan por crisis urgentes. Se han registrado el momento de las crisis, el momento de activación del SEM, el tratamiento prehospitalario, el momento del EEG y el diagnóstico final. **Resultados**: Se han recogido 1887 episodios, el 50,9% (960) atendidos por el SEM; en 502 se registró el tiempo de activación. De estos pacientes, 90 tenían un EE. La atención prehospitalaria llegó más tarde en los pacientes con EE (25 vs 55 minutos, *p* = 0,006). La mayoría de los pacientes (78,2%) no recibieron ningún tratamiento, si bien los pacientes con EE recibieron más veces tratamiento (38,9% vs 14,6%, *p* < 0,0001). Se realizó EEG en 285 pacientes, 87 con EE. Sobre los tiempos de acceso al EEG, no hubo diferencias significativas (15,5 vs 17 horas, *p* = 0,518). **Discusión/Conclusiones**: Los pacientes con EE reciben atención prehospitalaria tardía y a menudo no reciben tratamiento adecuado. El tiempo a EEG urgente es prolongado y similar para todos los pacientes. Es necesario optimizar los protocolos para garantizar un diagnóstico y tratamiento más rápido y efectivo.

**Caso Clínico: Epilepsia Musicógena de Semiología Temporal Mesial con Anticuerpos anti-GAD Positivos**

Yamaris Vera Castillo^1^, Javier Francisco Salas Jordán^1^, Tatiana Enrique Sagué^1^, Mar Mascarell Polache^1^, Ruth Victorio Muñoz^1^, Mika Aiko Gesler^1^, Paula Cases Bergón^1^

^1^Hospital Clínico Universitario de Valencia, Valencia, España

**Introducción y objetivos**: La epilepsia musicógena es una forma rara de epilepsia refleja, con prevalencia aproximada de 1 cada 10 millones de habitantes. Se caracteriza por crisis desencadenadas por estímulos musicales. Se han propuesto múltiples etiologías estructurales y disinmunes, sin embargo, la mayoría de los casos son idiopáticos. El tratamiento implica evitar estímulos desencadenantes junto a tratamiento anticrisis, hasta cirugía. En los últimos tiempos se ha asociado a anticuerpos anti-GAD generando una nueva ventana terapéutica. **Material y métodos**: Exponemos el caso de una paciente con crisis epilépticas inducidas por estímulo músical. Se revisaron la historia clínica y electroencefalogramas (EEGs) para obtener los datos. **Resultados**: Mujer de 52 años, diestra, con antecedentes de anoftalmía derecha, meningitis meningocócica en la infancia. Traumatismo cráneo encefálico por accidente de moto en 2014. Primera crisis en 2015, iniciando seguimiento en neurología por crisis epilépticas recurrentes. Se describen dos tipos de crisis (focales y generalizadas). Las focales consisten en movimiento anómalo de mano derecha con discurso incoherente. Las generalizadas, de aparición nocturna, son crisis tónico-clónicas, de duración variable y breve periodo post-crítico. Examen físico y neuropsicológico normal. Se realizan EEG seriados dentro de la normalidad hasta que en 2021 relaciona sus crisis focales con música de alto contenido emocional. En ese momento se realiza Video-EEG con música balada registrándose una crisis electroclínica focal en regiones fronto-temporales izquierdas (F7-T3) de 70 segundos. Curso de la enfermedad con difícil control farmacológico.** Discusión/Conclusiones**: La epilepsia refleja musicógena es una forma poco prevalente de epilepsia con mal control farmacológico, donde la música es el principal desencadenante, destacando la reciente asociación de este tipo de epilepsia con la encefalitis autoinmune.

**EEG y Encefalitis en Pediatría. A Propósito de Varios Casos**

Africa Bueno García^1^, Christian Hernández Aranda^1^, María Martín Carretero^1^, Gonzalo Ferrer Ugidos^1^, Sara Muniesa Lacasa^1^, Jose Miguel León Alonso Cortés^1^, Margely Abete Rivas^1^

^1^Hospital Clínico Universitario de Valladolid, Valladolid, España

**Introducción y objetivos**: Las encefalitis de origen desconocido en edad pediátrica presentan un desafío clínico por la falta de claridad etiológica. Las condiciones inflamatorias cerebrales presentan clínica diversa y su causa permanece sin identificar. El análisis de los patrones electroencefalograma (EEG) proporcionan una información importante para su diagnóstico, seguimiento y tratamiento, contribuyendo así a una mejor comprensión fisiopatológica y optimización de la atención clínica. Nuestro objetivo es analizar las características comunes en EEG, y su utilización. **Material y métodos**: Estudio descriptivo y retrospectivo. Se incluyeron pacientes con diagnóstico de encefalitis de causa desconocida, durante un año. Se realizaron serologías, pruebas de imagen, así como EEG a las 24 horas. Se registraron antecedentes personales, presentación clínica, evolución, así como su situación clínica al alta. **Resultados**: Incluimos 6 pacientes, media de 9 años de edad, 66% varones; sin antecedentes personales de interés. Síntomas clínicos más relevantes fiebre, disminución del nivel de conciencia, cefalea, crisis focales, vómitos, alteración conductual y de la marcha. En el 50% se detectaron infecciones por virus; todos los cultivos de LCR negativos. Los primeros hallazgos EEG mostraron actividad basal adecuadamente organizada; actividad basal globalmente lentificada con asimetría interhemisférica izquierda, y actividad lentificada sobre regiones frontales anteriores. Los últimos EEG mostraron normalidad, sobrecarga de ondas lentas sobre regiones anteriores otros sobre cuadrantes posteriores. **Discusión/Conclusiones**: El uso del EEG es una herramienta importante para la evaluación y seguimiento de la actividad cerebral. Los patrones observados en los pacientes estudiados presentaron diversas características, observándose en la gran mayoría un enlentecimiento de su actividad basal. Estos hallazgos resaltan la complejidad de estas condiciones y la necesidad de seguir investigando para mejorar la comprensión fisiopatológica y optimizar la atención clínica de los pacientes.

**Epilepsia del Área Motora Suplementaria en Contexto de Síndrome de Microdeleción 16p13.11: Presentación de un Caso**

Edward Emerson Susanibar Mesias^1^, Alba González García^1^, Diana Estephania Blanco Gómez^1^, Luisa Panades de Oliveira^1^, Ion Álvarez Guerrico^1^

^1^Hospital del Mar, Barcelona, España

**Introducción y objetivos**: El síndrome de microdeleción 16p13.11 tiene una penetrancia incompleta y expresividad variable. Puede cursar con retraso del desarrollo y lenguaje, trastornos psiquiátricos y crisis epilépticas de semiología variable, entre otros. Su implicación en casos de epilepsia del área motora suplementaria (AMS) no está claramente definida. El electroencefalograma (EEG) en epilepsia de lóbulo frontal puede mostrar hallazgos inespecíficos, principalmente en focos ubicados en áreas más profundas. **Material y métodos**: Descripción de un caso, monitorización vídeo-EEG prolongado y discusión. **Resultados**: Varón de 30 años con debut de epilepsia a los 3, farmacorresistente, además de dismorfia facial, microcefalia, y discapacidad intelectual leve. El análisis cromosómico por microarray detectó una deleción heterocigota 16p13.11. Su epilepsia cursa con crisis focales que pueden empezar con una sensación eléctrica en mano derecha, a veces asociada a postura distónica, y que a menudo evolucionan a elevación tónica asimétrica de miembros superiores. La monitorización vídeo-EEG registró múltiples crisis con correlato EEG en forma de respuesta electrodecremental inicial focal frontal izquierda con difusión progresiva, seguida de actividad theta-delta irregular con elementos agudos sobreañadidos, leve atenuación de la actividad subsiguiente y recuperación de la actividad basal. Se decidió estereoelectroencefalografía (SEEG) y electrotermocoagulación local, con persistencia de crisis, y resección quirúrgica de AMS izquierda dos años después, con mejoría clínica parcial. **Discusión/Conclusiones**: Los pacientes con síndrome de microdeleción 16p13.11 pueden presentar crisis del AMS. Por la ubicación del foco epiléptico, medial y profundo en el lóbulo frontal, el EEG puede suponer un reto diagnóstico, siendo importante reconocer el patrón electrodecremental difuso como un posible patrón ictal de esta localización.

**Implementación de Escala 2HELPS2B en Trazados de Pacientes con Sospecha de Crisis Epilépticas no Convulsivas**

Valeria Gallo Rivero^1^, Paula Ortega^1^, Antonio Pedrera Mazarro^1^, Guillermo Martin Palomeque^1^, Ignacio Regidor Bailly^1^

^1^Hospital Universitario Ramón y Cajal, Madrid, España

**Introducción y objetivos**: La monitorización continua con electroencefalografía (cEEG) es un recurso limitado que debe usarse con prudencia para obtener resultados óptimos. En la última década, su uso para detectar crisis epilépticas no convulsivas (CENC) ha aumentado significativamente. Las directrices actuales recomiendan al menos 24 horas de monitorización para detectar CENC. La escala 2HELPS2B, basada en parámetros de EEG y factores clínicos, puede ayudar a estratificar el riesgo de CENC y mejorar la toma de decisiones. **Material y métodos**: Se realizó un estudio observacional y retrospectivo, considerando 75 trazados de electroencefalograma (EEG) de pacientes pediátricos y adultos con sospecha de CENC, desde enero hasta mayo de 2024. Aplicamos la escala 2HELPS2B para valorar la necesidad de cEEG. Excluimos pacientes con antecedentes de parada cardiaca, sospecha de encefalopatía y estatus epiléptico convulsivo. Revisamos los registros EEG para determinar si cumplían con algún parámetro de la escala, como: frecuencia >2 Hz, descargas epilépticas esporádicas, LPD, BIPD, GRDA, crisis epilépticas previas (1 punto cada uno) o BIRDS (2 puntos), para determinar la necesidad de cEEG. **Resultados**: Tras la implementación de la escala 2HELPS2B, observamos que 52% (39) de los trazados superaban los 2 puntos (alto riesgo de CENC), 28% (21) tenían riesgo intermedio (1 punto) y 20% (15) tenían bajo riesgo (0 puntos). Dada la limitada disponibilidad de recursos, el cEEG se realizó tan solo a 15 pacientes con alto riesgo de CENC. **Discusión/Conclusiones**: El cEEG es una herramienta limitada a nivel mundial. Este estudio plantea la necesidad de adaptar la escala 2HELPS2B en centros con menor disponibilidad de recursos, manteniendo su validez para detectar CENC en pacientes de alto riesgo. Además, sería útil aumentar el tamaño de la muestra para mejorar el poder estadístico del estudio.

**Implementando el Código Crisis: el Valor de la Enfermería de Neurofisiología Clínica**

María del Pilar Cabrerizo^1^, Elena Cortés García^1^, Rosa María Díaz Sánchez^1^, Sandra Rodrigo Heredia^1^, Marina Sanjurjo Morote^1^, Paloma Canalejas Alcantara^1^

^1^Hospital Clínico San Carlos, Madrid, España

**Introducción y objetivos**: En 2024 se ha instaurado en la Comunidad de Madrid el Código Crisis, enfocado a la atención temprana en los pacientes que sufren crisis epilépticas con criterios de gravedad ya sean en el ámbito extrahospitalario como en el intrahospitalario, en los que el riesgo y/o intauración del estatus epiléptico requiere de una pronta actuación. Al ser un programa de atención de nueva instauración no se dispone de protocolos de Enfermería específicos de actuación en este ámbito. Creación de un protocolo de Enfermería para la actuación del personal de neurofisisología en este tipo de códigos enfocado en una atención temprana y eficiente con una monitorización precoz del paciente. **Material y métodos**: Se crea un protocolo con los paso específicos para la actuación de Enfermería. Se crea un registro de pacientes que son atendidos tanto en el turno de mañana como en el turno de guardia del código crisis. **Resultados**: Creación de un protocolo de Actuación de Enfermería específico para Código Crisis. Se instaura un registro de pacientes atendidos por Neurofisiología en el turno de mañana (L-V 08:00–15:00) y otro para los pacientes atendidos en turno de guardia de Código Crisis (L-V de 15:00–22:00 S-D de 08:00–22:00). Número de pacientes total atendidos desde el 8 de marzo de 2024 hasta el 6 de junio de 2024: 21 pacientes. Turno de guardia: 9 pacientes. Adultos (6 varones y 2 mujeres) y 1 niño. Turno de mañana: 12 pacientes. Adultos (3 hombre y 1 mujer) y 8 niños. **Discusión/Conclusiones**: La instauración del protocolo específico de Enfermería en la actuación del Código Crisis mejora la atención al paciente disminuyendo el tiempo de actuación médica ayudando así a preservar la función cerebral de los pacientes.

**Electroencefalograma Como Herramienta Diagnóstica en Unidades Críticas**

Javier Francisco Salas Jordan^1^, Yamaris Vera Castillo^1^, Tatiana Enriquez Sagué^1^, Mar Mascarell Polache^1^, Rut Victorio Muñoz^1^, Mika Aiko Gesler^1^, Paula Cases Bergón^1^

^1^Hospital Clínico Universitario de Valencia, Valencia, España

**Introducción y objetivos**: La monitorización del estado neurológico es esencial en el paciente crítico con sospecha de lesión cerebral aguda. El electroencefalograma (EEG) proporciona información de la actividad neuronal ante cambios estructurales y/o funcionales, de manera sencilla y no invasiva. Su utilización ha aumentado como herramienta diagnóstica fundamental de estados epilépticos convulsivos y no convulsivos, detección de isquemia cerebral, encefalopatías, profundidad de sedación, pronóstico del paciente comatoso y muerte encefálica. El EEG rutinario continúa siendo una alternativa coste-efectiva para los centros donde no hay monitorización continua. **Objetivo**: Evaluar la indicación y utilidad diagnóstica del EEG en unidades críticas. **Material y métodos**: Se realizó un estudio transversal en 174 sujetos mayores de 16 años, ingresados en las unidades de cuidados intensivos (UCI) y reanimación (REA) del Hospital Clínico Universitario de Valencia, entre enero y diciembre de 2023, con indicación de electroencefalograma. Los datos se extrajeron de las historias clínicas. Se consideraron variables como edad, sexo, origen de la solicitud, motivo de indicación, diagnóstico de EEG. **Resultados**: El 60% de los pacientes eran hombres, con edades comprendidas entre 17 y 91 años y una media de 62,5%. El 54,6% era de UCI. Las indicaciones más frecuentes de EEG fueron: sospecha de crisis y/o estatus epiléptico (19,6%), deterioro del nivel de consciencia (15%), enfermedad cerebrovascular hemorrágica (23,6%), no adecuada progresión neurológica (14,4%) y encefalopatía hipóxica isquémica (12%). Los diagnósticos de EEG principales fueron encefalopatía (55%) y presencia de actividad epileptiforme (21%), dentro de esta última el estatus epiléptico no convulsivo representó el 4%. **Discusión/Conclusiones**: El EEG sigue siendo una herramienta fundamental para el diagnóstico, pronóstico y orientación terapéutica del paciente crítico.

**EEG de Urgencia Solicitados al Servicio de Neurofisiología Clínica del Hospital de Santiago de Compostela**

Alexandra Margarita Navarrete Loza^1^, José Luis Relova Quinteiro^1^, Elva Pardellas Santiago^1^, Walkiria Soto Cruz^1^

^1^Hospital Clínico Universitario de Santiago de Compostela, A Coruña, España

**Introducción y objetivos**: El electroencefalograma (EEG) urgente (EEG-U) se realiza en situaciones de riesgo para el sistema nervioso central, entre otras para prevenir daños o secuelas. El objetivo de este estudio ha sido categorizar los EEG-U durante un periodo de 4 meses, para establecer el contexto de la solicitud, los hallazgos del EEG y su impacto en la toma de decisiones. **Material y métodos**: Diseño observacional con muestra prospectiva secuencial. Los EEG se realizaron e informaron por el servicio de Neurofisiología Clínica. Se aplicaron escalas de utilidad del EEG; la estadística se realizó con SPSSv29. **Resultados**: Se realizaron 453 EEG-U a 297 pacientes, 88% de ellos ingresados, con edad media 53 años. La mayoría fueron solicitados por UCI-A (16%), neurología (15%) y urgencias (15%). Un 24% requirió EEG sucesivos. Los motivos de consulta fueron sospecha de estatus no convulsivo (46%), 1º crisis epiléptica (28%), crisis sucesivas o completar estudio clínico (11%) y para diagnóstico de muerte cerebral (1,1%). Con alteración de la consciencia (48%) o sospecha de movimientos sutiles (18%) como presentación clínica. Los patrones mas frecuentes fueron 211 EEG-U normales (47%), 94 con actividad epileptiforme bilateral (21%) y 55 con lentificación difusa (12%). Estos hallazgos determinaron el mantenimiento (72%), modificación del tratamiento (24%), o inicio de FAEs (4%), siendo el EEG-U útil en el diagnóstico (58%, n = 264) o en el cambio del tratamiento (26%, n = 117) de estos pacientes. **Discusión/Conclusiones**: Se destaca la elevada demanda de EEG-U, un 53% de todos los EEG hechos en el servicio; 386 en jornada ordinaria (67% en cabecera del paciente) y 67 en jornada de guardia (15%, superior a datos previos). Gracias al EEG-U se optimizó el diagnóstico y tratamiento de los motivos de consulta frecuentes descritos, además de guiar el manejo de otras patologías, en paciente pediátrico y adulto, sobre todo neurocríticos. La posibilidad de realización de EEG-U las 24 horas por Neurofisiología Clínica es fundamental, siendo necesario protocolos de EEG-U en los hospitales.

**Estado de Ausencias Fantasmas**

Gabriel Jesús Planchart Gómez^1^, Luis Santoveña González^2^, Ernesto Vargas Díaz^1^, María Esperanza Pérez Álvarez^1^, Jessica Soto García^1^, Ledy Marisol García Bu^1^, Isabel Aparicio Fernández^1^

^1^Complejo Asistencial Universitario de León, León, España

^2^Hospital Universitario Central de Asturias, Oviedo, España

**Introducción y objetivos**: Las “ausencias fantasmas” son breves crisis epilépticas de 2 a 4 segundos descritas por Panayiotopoulos *et al*. en 1997. Pasan desapercibidas y no afectan significativamente la vida diaria, aunque pueden causar problemas de concentración o memoria. La mitad de los pacientes presentan un estado epiléptico de ausencia con deterioro cognitivo leve a moderado y problemas de comunicación. El electroencefalograma (EEG) muestra actividad anormal, mientras que otras pruebas suelen ser normales. La ILAE las considera un posible resultado de la maduración cerebral, no un síndrome. **Material y métodos**: Se presenta el caso clínico de un niño de 12 años que tuvo un desarrollo prenatal y postnatal normal. En octubre de 2020, comienza a mostrar apatía, respuestas incoherentes, llanto inexplicable y somnolencia, normalizándose tras dormir. Episodios posteriores incluyen cefalea, cambio de carácter, habla incoherente, mirada fija y supraversión ocular. Se realiza V-EEG compatible con ausencias. Inicialmente, se diagnosticaron migraña confusional, migraña basilar y epilepsia tipo ausencias, iniciando tratamiento con ácido valproico. **Resultados**: Como resultado y tras dos años sin crisis, se retiró lentamente el VPA, pero las crisis reaparecieron. Un V-EEG mostró actividad discontinua con brotes de punta- onda generalizadas de no más de 4 segundos de duración, sin llegar a normalizar actividad basal entre brotes acompañado clínicamente por alteración de la consciencia. Dicha descripción es compatible con estado epiléptico, justificando la reintroducción del VPA, con mejoría clínica evidente. **Discusión/Conclusiones**: Este caso destaca la dificultad de diagnosticar las ausencias fantasmas, que pueden confundirse con otras patologías como migraña. Las ausencias son breves, con síntomas como aleteo palpebral y supraversión ocular, y las pruebas EEG son cruciales para su identificación. Un diagnóstico adecuado y temprano permite un tratamiento oportuno y efectivo, siendo el V-EEG una herramienta esencial para diferenciar esta entidad.

**Hallazgos Electroclínicos en el Síndrome de Pitt-Hopkins: A Propósito de 4 Casos**

Pilar Rosario Martínez Martínez^1^, Sofía Ortigosa Gómez^1^, Francisca Valera Párraga^1^, Carmen María Garnés Sánchez^1^, Reetika Mukesh Baharani Baharani^1^, Víctor Hugo Rubio Suárez^1^, Julián Vázquez Lorenzo^1^

^1^Hospital Clínico Universitario Virgen de la Arrixaca, Murcia, España

**Introducción y objetivos**: El síndrome de Pitt-Hopkins (PTHS) es un trastorno en el neurodesarrollo poco común producido por mutaciones, por lo general *de novo*, en el gen *TCF4* (18q21). Cursa con discapacidad intelectual, rasgos faciales característicos y un patrón de respiración anómalo. Alrededor del 40% llegan a desarrollar epilepsia de evolución variable. **Material y métodos**: Estudio retrospectivo sobre el seguimiento clínico y neurofisiológico de 4 pacientes diagnosticados de PTHS en el HCUVA (Murcia). **Resultados**: Muestra de 4 pacientes con edad media de 5,5 años (rango de edad entre 16 meses y 12 años): Uno presenta una variante potencialmente patogénica del gen *TCF4* y tres presentan delección patogénica en el cromosoma 18. Todos cursan con fenotipo de PTHS. Dos nunca presentaron crisis epilépticas, uno presentó episodios sugestivos de síncopes convulsivos secundarios a apneas vs. crisis convulsivas y otro presentó espasmos epilépticos. A todos se les realizaron vídeo-electroencefalograma (EEG) seriados, resultando uno sin anomalías epileptiformes, dos presentaron esporádica actividad epileptiforme intercrítica focal, y otro presentó numerosas crisis electroclínicas compatibles con espasmos epilépticos agrupadas en salvas. La actividad de fondo fue normal en tres de los pacientes. **Discusión/Conclusiones**: Hay 3 tipos de episodios paroxísticos a diferenciar en el PTHS: eventos respiratorios anormales, estereotipias motoras y crisis epilépticas, estas últimas pueden aparecer a lo largo de la vida de los pacientes. Así, se precisa un seguimiento médico estrecho, siendo el vídeo-EEG una herramienta útil para filiar dichos episodios. Según estudios publicados no hay un patrón EEG característico e incluso puede resultar normal. Además, hay una gran variedad de mutaciones que afectan al gen *TCF4*, planteándose correlaciones genotipo-fenotipo.

**Optimización de la Cirugía de Epilepsia Mediante Corregistro EEG-SPECT Ictal: Nuestra Experiencia y Hallazgos**

Laura Lorente Remón^1^, Miguel Cobo Moreno^1^, Antonio Lucas McHugh^1^, Teresa Ortega León^1^, Alberto Galdón Castillo^1^

^1^Hospital Universitario Virgen de las Nieves de Granada, Granada, España

**Introducción y objetivos**: En la Unidad Multidisciplinar de Cirugía de Epilepsia del Hospital Universitario Virgen de las Nieves (HUVN) de Granada, se llevó a cabo un estudio con el objetivo de analizar la concordancia entre los resultados obtenidos mediante electroencefalograma (EEG) y tomografía computarizada por emisión de fotón único (SPECT) en pacientes con epilepsia. Este estudio busca optimizar el diagnóstico y tratamiento de la epilepsia, mejorando así la precisión en la localización de focos epileptógenos mediante el uso combinado de estas dos técnicas neurofisiológica y de imagen. **Materia**l** y métodos**: Se realizó un estudio descriptivo y retrospectivo con una muestra de 12 pacientes (3 mujeres y 9 hombres) con edades comprendidas entre 4 y 30 años, a quienes se les practicó un SPECT ictal entre 2021 y 2024 en la Unidad de Epilepsia del HUVN. **Resultados**: Los pacientes presentaban diversos tipos de epilepsia: 5 tenían epilepsia frontal, 3 epilepsia temporal, 2 afectación hemisférica izquierda, 1 focalidad inespecífica y 1 epilepsia en línea media. La inyección del trazador se realizó en menos de 10 segundos en 10 de los 12 pacientes, y en 12 segundos en los 2 restantes. Los resultados mostraron que en 6 pacientes la zona epileptiforme identificada por el SPECT coincidió con la zona epileptogénica determinada por el video-electroencefalograma (VEEG); en 3 casos, el VEEG no fue concluyente; en 2 casos, el SPECT fue no concluyente; y en 1 paciente no hubo coincidencia entre el SPECT y el VEEG. Pese a las limitaciones debidas al bajo tamaño muestral, nuestra experiencia indica que la correspondencia entre el EEG ictal y la localización del SPECT ictal mostró un buen rendimiento diagnóstico. Los hallazgos sugieren que el SPECT ictal puede ser una herramienta útil para la localización de la zona epileptogénica. **Discusión/Conclusiones**: Se requiere más investigación para optimizar su uso y mejorar la concordancia diagnóstica con el VEEG, especialmente en casos donde los estudios no son concluyentes.

**Relevancia del vídeo-EEG Prolongado en la Epilepsia con Crisis Hipermotoras: Una Serie de Casos**

Alberto Ulloa Meijide^1^, Carina Diéguez Varela^1^, Beatriz Soria Soriano^1^, Iria Lagoa Labrador^1^, Iván Seijo Raposo^1^

^1^Hospital Álvaro Cunqueiro de Vigo, Vigo, España

**Introducción y objetivos**: La epilepsia con crisis hipermotoras asociadas al sueño es un síndrome raro (1,8–1,9 por 100.000 habitantes), caracterizado por crisis focales motoras con componente hipercinético o tónico/distónico asimétrico, acompañado habitualmente de signos autonómicos, vocalizaciones, ocasionalmente desviación ocular y/o cefálica y evolución a crisis tónico-clónicas generalizadas. Describimos una serie de pacientes de nuestro centro con este tipo de epilepsia. **Material y métodos**: Se realizó una revisión retrospectiva de la historia clínica y registros vídeo-electroencefalograma (EEG) disponibles de los pacientes en los que hemos registrado crisis hipermotoras en sueño en los últimos 12 meses. **Resultados**: Identificamos 5 pacientes (10–42 años), todos ellos con registro vídeo-EEG prolongado (2,5–118 h). Todos los pacientes presentaron EEG previos sin hallazgos significativos. En el vídeo-EEG prolongado 2 presentaron actividad epileptiforme intercrítica en sueño y ninguno en vigilia. El EEG ictal de 4 pacientes consistió en anomalías epileptiformes de predominio frontal (uni o bilateral), y uno actividad generalizada. Hubo una gran variabilidad interindividual en la semiología de las crisis. **Discusión/Conclusiones**: El EEG de rutina es normal en la mayoría de pacientes con epilepsia con crisis hipermotoras asociada al sueño. El vídeo-EEG prolongado es de gran importancia para identificar este tipo de crisis, así como anomalías epileptiformes intercríticas en estos pacientes, con mayor rentabilidad al aumentar la duración. En nuestro centro han sido de gran importancia los registros prolongados para el diagnóstico diferencial con pseudocrisis en este tipo de pacientes.

**Efectividad del Electroencefalograma Precoz en una Consulta de Primera Crisis**

José Antonio Pérez Martínez^1^, Laura López Viñas^1^, Marina Gómez-Villaboa Benítez^1^, Estuardo Daniel Castro Ruiz^1^, María José Postigo Pozo^1^, Alejandro Sarmiento Pita^1^, Victoria Eugenia Fernández Sánchez^1^

^1^Hospital Regional Universitario de Málaga (HRUM), Málaga, España

**Introducción y objetivos**: Las crisis epilépticas (CE) son una urgencia neurológica potencialmente grave, siendo fundamental su diagnóstico precoz. Para ello, hemos constituido una Consulta de Primera Crisis (C1ªC), entre Neurología y Neurofisiología Clínica, donde se realiza electroencefalografía (EEG) y reevaluación clínica a las 48–72 horas, tras la sospecha de 1ª CE con asistencia a urgencias. **Material y métodos**: Estudio retrospectivo de los 54 pacientes atendidos en C1ªC, (2023–mayo 2024). **Resultados**: 35 de los 54 pacientes (64.8%), presentaron episodios con alta sospecha de CE y 19 pacientes (35.1%), presentaban sospecha de otros episodios (síncopes, migrañas o trastornos paroxísticos no epilépticos). 18 pacientes (51,4%) eran sospechas de CE focales y 17 (48,5%) de generalizadas. En EEG de la C1ªC, de los 18 pacientes con sospecha de CE focales, 13 presentaron paroxismos focales (72%); de los 17 pacientes con sospecha de CE generalizadas, 7 presentaron paroxismos generalizados (41,1%). En los 19 pacientes con sospecha de otros eventos, el EEG fue normal en 15 (78,9%) y en 3 se registraron paroxismos focales (15,7%) y 1 privación enólica. El EEG orientó el tratamiento con medicación anticrisis (MACs): (1) En 30 pacientes se inició MAC empírica por la alta sospecha de CE y en todos, tras EEG con paroxismos se decidió seguir con MACs, redirigiendo la MAC empleada a otro ppio activo en 15 pacientes. (2) Tras hallazgos de paroxismos en el EEG, 4 pacientes que inicialmente no fueron tratados con MACs, iniciaron tratamiento: 2 CEfocales, y 2 CE generalizadas. (3) 3 pacientes, 2 sin CE y 1 sospecha de CE generalizadas, inicialmente fueron tratados con MACs y se retiraron MACs tras EEG normal. (4) 16 pacientes no tuvieron MAC previa ni posterior al EEG, normal en 14 de éstos. **Discusión/Conclusiones**: La C1 ºC es un elemento de gran valor para filiar posibles crisis, así como proporciona información para la rápida modificación del tratamiento, que conlleva una optimización del mismo y una mejora coste-utilitaria en pacientes con epilepsia de reciente diagnóstico.

**Papel del EEG en la Afasia de Instauración Aguda**

Paula Ortega García^1^, Valeria Gallo Rivero^1^, Antonio Pedrera Mazarro^1^, Guillermo Martin Palomeque^1^

^1^Hospital Universitario Ramon y Cajal, Madrid, España

**Introducción y objetivos**: La afasia de instauración brusca es una urgencia neurológica frecuente, siendo su etiología principal la patología vascular, en el contexto de la hipoperfusión, si bien, puede ser la manifestación de origen epiléptico. Un resultado negativo en la prueba de neuroimagen disponible en el momento agudo como el TC multimodal (TCMM), no permite descartar el origen vascular, y se puede considerar el estudio electroencefalograma (EEG) para descartar la presencia de una posible etiología epiléptica, pues en este caso, un resultado negativo sí permite descartar razonablemente el origen epiléptico. El objetivo de este estudio es describir las alteraciones electroencefalográficas de los pacientes con alteración brusca del lenguaje sin evidencia de anomalías en el TCMM en el momento agudo y correlacionarlas con la causa identificada a posteriori en estudios de neuroimagen más sensibles (RM). **Material y métodos**: Estudio observacional prospectivo de una muestra de 27 pacientes de entre 35 y 91 años, procedentes de peticiones de EEG urgente por afasia brusca y TCMM normal, seguidos desde diciembre de 2023 hasta junio de 2024. Se recogieron datos clínicos (edad, antecedentes de lesiones estructurales encefálicas previas, nivel de conciencia y alteración del lenguaje) así como datos de las pruebas complementarias (TCMM, EEG y RM). **Resultados**: Del total de estudios EEG 6 fueron normales y 21 patológicos (77%), de los cuales 14 (66,6%) presentaron anomalías localizadas en el hemisferio izquierdo, y 3 de ellos cumplieron criterios de status epiléptico no convulsivo (SENC). Del total de pacientes, se realizó RM craneal a 20 de ellos, siendo un 70% patológicos, incluidos 2 pacientes con SENC (meningoencefalitis autoinmune hemisférica izquierda y una fistula dural extensa izquierda). Ningún paciente con EEG normal presentó una lesión en la RM. **Discusión/Conclusiones**: El EEG es una herramienta útil para descartar razonablemente la presencia de actividad epiléptica en contexto de afasia de instauración aguda.

**Descargas Rítmicas Subclínicas en el EEG del Adulto (SREDA) en un Niño: Reporte de un Caso y Revisión de la Literatura**

Gisel Montoya Aguirre^1^, Vicenç Pascual Rubio^1^, Ana Paloma Polo^2^

^1^Hospital Universitario Sant Joan de Reus, Reus, España

^2^Hospital Universitario Gregorio Marañón, Madrid, España

**Introducción y objetivos**: Las descargas rítmicas subclínicas en el electroencefalograma (EEG) del adulto (SREDA) son una variante rara y benigna, típicamente observada en adultos. Se caracterizan por actividad theta rítmica temporo-parietal bilateral, que puede parecer una crisis electrográfica pero termina abruptamente sin enlentecimiento posterior ni alteración de la conciencia. Su significado sigue siendo incierto. La aparición de SREDA en niños es extremadamente rara, con solo cinco casos publicados, la mayoría asociados con epilepsia generalizada, lo que sugiere una posible relación, aunque es un patrón infradiagnosticado. Este trabajo presenta el caso de un niño con un patrón EEG atípico, discute los desafíos diagnósticos y revisa la literatura existente. **Material y métodos**: Se revisó el caso de un niño de 8 años remitido a neurofisiología con sospecha de TDAH y dudosos episodios de desconexión. Se analizaron su historia médica, registros EEG y resonancia magnética (RMN). Se realizó una revisión de la literatura sobre SREDA en niños para contextualizar los hallazgos. **Resultados**: El paciente, remitido para descartar crisis epilépticas, presentaba antecedentes de embarazo con diabetes gestacional, desarrollo psicomotor normal y madre con probable TDAH. Durante el EEG, se observaron salvas/trenes bilaterales de actividad theta rítmica temporal y parieto-occipital, sin manifestaciones clínicas ni enlentecimiento posterior. La RMN fue normal y los EEG subsecuentes confirmaron estos hallazgos. **Discusión/Conclusiones**: Este caso presenta un probable patrón SREDA en un niño, comprobado en EEGs sucesivos. SREDA es un patrón generalmente benigno y observado en adultos. Su rareza extrema en niños y su ritmicidad pueden llevar a interpretaciones erróneas. Algunos autores han sugerido denominarlo “descargas rítmicas subclínicas en el EEG de adultos y niños (SREDAC)”. Este caso discute los desafíos diagnósticos y revisa la literatura para comprender mejor este fenómeno, subrayando además la importancia de reconocer este patrón en niños para evitar confundirlo con actividad ictal.

**Revisión de Casos de Estatus Epiléptico no Convulsivo en la Unidad de Cuidados Intensivos de un Hospital Comarcal**

Ana María Muñoz Mateo^1^, Habib Halim Azzi^1^, Cristina Ipiéns Escuer^1^, Andrés Felipe Salas Redondo^1^, Celio Valentín Martínez Ramírez^1^, Francisco Javier Puertas Cuesta^1^

^1^Hospital Universitario de la Ribera, Alzira, España

**Introducción y objetivos**: El estatus epiléptico no convulsivo (EENC) es una emergencia neurológica, por lo que es importante hacer un diagnóstico precoz. El objetivo de esta revisión es analizar las características de los pacientes con EENC, tanto clínicas como electroencefalográficas, además del manejo y desenlace de los mismos, en el Hospital Universitario de la Ribera (Valencia). **Material y métodos**: Estudio observacional retrospectivo que incluyó los pacientes ingresados en la UCI con diagnóstico de EENC, desde agosto de 2017 hasta abril de 2024. Para el diagnóstico de EENC se cumplieron los criterios de Salzburgo. Se registró la edad, sexo, antecedentes de epilepsia, etiología, hallazgos electroencefalográficos, tratamiento, tiempo de ingreso en UCI y supervivencia. **Resultados**: 53 pacientes, 30 mujeres y 23 hombres. Edad media 64 años. Solo 11 personas con antecedentes personales de epilepsia. 37 de ellos no asociaron clínica motora al ingreso en UCI ni durante el diagnóstico de EENC. La hemorragia intracraneal fue la etiología más frecuente (41,5% de los casos). El 69,8% de los pacientes se encontraba en coma al realizar el EEG. Dentro de las variables electroencefalográficas, destacan como grafoelementos las puntas (en el 90,57% de los electroencefalogramas) y ondas agudas (90,57%). Se describió actividad delta rítmica monomorfa de alto voltaje en el 43,4%. 2 pacientes mostraron descargas epileptiformes periódicas lateralizadas. El 81,1% de los pacientes muestran la actividad de forma difusa o generalizada, de los cuales el 43,4% manifiestan predominio frontal. El 73,6% de los pacientes llevaba tratamiento antiepiléptico en el momento de realizar el EEG, llegando al 94,34% tras ser identificado el EENC. Finalmente, la mortalidad durante su ingreso en UCI es del 43,4%. **Discusión/Conclusiones**: El EENC se ha documentado con frecuencia en la UCI de este hospital comarcal, por lo que es primordial conocer bien los hallazgos del EEG para un mejor manejo de los pacientes.

**Ayuda del EEG en el Diagnóstico y Tratamiento de la Encefalopatía de Hashimoto, en un Caso Pediátrico**

María Esperanza Marín Serrano^1^, María Lorenzo Ruiz^1^, Julián Lara Herguedas^1^, Rosario Cazorla Calleja^1^, Gema Iglesias Escalera^1^, Luis Fernando López Pájaro^1^, Edwin Eduardo Ebrat Mancilla^1^

^1^Hospital Universitario Puerta de Hierro Majadahonda, Madrid, España

**Introducción y objetivos**: La tiroiditis de Hashimoto es la causa más frecuente de disfunción tiroidea en niños. La encefalopatía de Hashimoto se asocia con anticuerpos antitiroideos elevados y es una forma rara pero grave de encefalopatía en pediatría, siendo importante el diagnóstico precoz. Exponemos un caso pediátrico en el que el estudio electroencefalograma (EEG) ayudó a mejorar el enfoque terapéutico y revisamos las características clínicas y del EEG en estos paciente. **Material y métodos**: Niña de 10 años que ingresa porque desde hacía 6 días la notaban inatenta, diferente, con movimientos coreicos, alteraciones de sueño y torpeza motora. Afebril y sin antecedentes infecciosos en el último mes. Con historia de tiroiditis desde feb-2019, pero sin tratamiento al estar asintomática. En nov-2023 empezó a presentar alteraciones de sueño con RMN normal. En marzo 2024 se asocia algo de irritabilidad, cansancio y dificultad para dormir e inician Eutirox, pero aún no se solicitó un EEG. En los artículos publicados, los síntomas que pueden presentar los pacientes pediátricos son variados e incluyen crisis GTC, estado epiléptico, problemas de comportamiento, cefaleas, coma, problemas de sueño etc. Los estudios EEG muestran lentificación de la actividad de fondo, descargas epilépticas, encefalopatía y otras alteraciones inespecíficas. **Resultados**: Aunque la niña había mejorado al iniciar los corticoides, estaba algo somnolienta e irritable y nuestro EEG mostró datos de encefalopatía de grado leve-moderado por lo que decidieron añadir tratamiento con inmunoglobulinas, tras lo que la niña mejoró aún más. **Discusión/Conclusiones**: Los síntomas de la encefalopatía de Hashimoto pediátrica son muy diversos y el EEG resulta muy útil para un diagnóstico precoz y mejor ajuste terapéutico. Planteamos la recomendación de realizar un estudio EEG basal en los niños con diagnóstico de tiroiditis de Hashimoto para poder compararlo en caso de que empiecen a presentar síntomas sugestivos de encefalopatía de Hashimoto, e intentar evitar llegar a complicaciones más graves.

**Valor Predictivo del Electroencefalograma en la Encefalopatía Hipóxico-Isquémica Tras Parada Cardiorrespiratoria**

Dora Sturla Carreto^1^, Karen López Viera^1^, Carmen Montes Gonzalo^1^, Raúl Sastre González^1^, Gemma Vázquez Casares^1^, M Victoria Alejos Herrera^1^

^1^Complejo Asistencial Universitario de Salamanca, Salamanca, España

**Introducción y objetivos**: La encefalopatía hipóxico-isquémica (EHI) tras una parada cardiorrespiratoria (PCR) es la tercera causa de coma en la unidad de cuidados intensivos (UCI). Los pacientes con EHI sobreviven cada vez más, aunque con grandes déficits. Predecir el resultado neurológico es crucial para informar adecuadamente a las familias, evitar tratamientos desproporcionados en casos irreversibles y asegurar una atención adecuada en casos con posibilidad de recuperación. El electroencefalograma (EEG) es una herramienta importante para predecir el desenlace tras daño cerebral. Nuestro objetivo fue relacionar patrones EEG específicos con el pronóstico neurológico. **Material y métodos**: Se revisaron retrospectivamente pacientes >18 años ingresados en la UCI por sospecha de EHI tras PCR, a los cuales se le solicitó un EEG al servicio de neurofisiología del Hospital de Salamanca, entre enero de 2022 y febrero de 2024. Se utilizó la Escala Modificada de Hockaday para establecer pronóstico benigno, maligno e incierto según el grado y los patrones EEG. Se revisitaron los EEG para clasificarlos según la terminología de la Sociedad Americana de Neurofisiología Clínica. Evaluamos el resultado neurológico al alta, utilizando la escala de cerebral performance category (CPC) (CPC 1–2 como resultado favorable y 3-4-5 desfavorable). **Resultados**: La muestra fue de 33 pacientes, 70% de sexo masculino y edad media de 65 años. El 25% (8) sobrevivió, de estos, 2 fallecieron en el primer mes de alta, clasificados con encefalopatía grado II y III y un CPC desfavorable. Todos los pacientes con grado I y II (2) sobrevivieron, pero con CPC desfavorable. De los supervivientes con grado III (4), el 50% logró un CPC favorable. Los supervivientes con grado IV (2) tenían un CPC desfavorable. El grado de encefalopatía más común fue el IV y el patrón EEG más frecuente fue el theta-delta. **Discusión/Conclusiones**: En patrones malignos el EEG es una herramienta útil para predecir el resultado neurológico. El resto de patrones tuvieron una menor rentabilidad pronóstica.

**Narcolepsia, no REMplaces mi Vida. A Propósito de un Caso**

Laís Alexandra Reinoso Aguirre^1^, María Carmen Lloria Gil^1^, Jennifer Paola Cantarero Durón^1^, Diego Francisco Peña Olaya^1^, Alina Havrylenko Vynogradnyk^1^, Boris Orozco Jimenez^1^, Joseba Soto Ibáñez^1^

^1^Hospital Universitario de Burgos, Burgos, España

**Introducción y objetivos**: La narcolepsia es uno de los principales trastornos del ciclo vigilia-sueño, caracterizada por una excesiva somnolencia diurna, necesidad imperiosa de dormir e incapacidad de estar de pie por falta de coordinación muscular. Es un trastorno complejo y multifactorial. Tiene una presentación generalmente esporádica, sin embargo entre el 10–15% de los casos presenta una agregación familiar, relancionandose principalmente con el gen *HLA-DQB1*. Este estudio pretender enfatizar la importancia de tener esta entidad como parte del diagnóstico diferencial de los episodios de pérdida de consciencia, a fin de lograr un diagnóstico temprano de la misma. **Material y métodos**: Paciente varón de 31 años que es derivado a la unidad de sueño por síncopes, con un inicio de los episodios en 2018, caracterizados por pérdida de consciencia de un minuto de duración, en ocasiones precedidos de sensación de malestar general. Añade, que tras la risa intensa nota debilidad en la boca y episodios de “verse desde arriba” que podría corresponder a alucinaciones hipnagógicas. PSG nocturna: con montaje electroencefalograma (EEG) reducido, EOG, EMG en mentón, EKG, banda torácica de esfuerzo respiratorio y pulsioxímetro y a la mañana siguiente, un test de latencias (TLMS), registrando 3 siestas.** Resultados**: PSG: 4 ciclos de sueño adecuadamente estructurados y con buena eficiencia (95.6%), latencia a sueño NREM muy acortada y a sueño REM discretamente acortada (67 min). TLMS: 3 siestas con entrada en REM en cada una (no se realizan más por resultado claramente positivo); Latencias acortadas (2,2 min) y a REM (7,7 min). **Discusión/Conclusiones**: La narcolepsia es un trastorno cerebral, a tener cuenta como parte del diagnóstico diferencial de los episodios de pérdida de consciencia, principalmente cuando éstos tienen características atípicas. Su diagnóstico muchas veces se retrasa varios años y lo que buscamos por medio de la difusión de estos casos clínicos es mejorarlo, a fin de realizar un diagnóstico oportuno con una clara repercusión en el bienestar y seguridad del paciente.

**Mononeuritis Múltiple Como Clave Diagnóstica de una Granulomatosis con Poliangeítis Eosinofílica: el Valor del ENG-EMG**

Moisés León Ruiz^1^, Andrea Gómez Moroney^1^, Manuel Lorenzo Diéguez^2^, María Almudena Martínez Pérez^1^, Susana Santiago Pérez^1^, Laura Lacruz Ballester^2^

^1^Sección de Neurofisiología Clínica, Servicio de Neurología, Hospital Universitario La Paz, Madrid, España

^2^Servicio de Neurología, Hospital Universitario La Paz, Madrid, España

**Introducción y objetivos**: La granulomatosis con poliangeítis eosinofílica (GPAE) es una vasculitis de pequeño vaso asociada anticuerpos anticitoplasma de neutrófilo (ANCA) en el 30–45% de casos, pudiendo producir neuropatía vasculítica. El patrón más frecuente es la mononeuritis múltiple (MM) seguida de la polineuropatía asimétrica (ambas sensitivo-motoras axonales). Presentamos una GPAE donde el electroneurograma-electromiograma (ENG-EMG) fue la clave diagnóstica. **Material y métodos**: Varón de 65 años, con rinitis crónica con pólipos nasales y EPOC reagudizador con 3 ingresos previos (con infiltrados pulmonares no cavitados en TAC pulmonar), traído a Urgencias por un cuadro de 20 días de parestesias y debilidad de miembro superior derecho, después miembro superior izquierdo (MSI), y finalmente miembros inferiores (MMII). La exploración reveló paresia distal asimétrica de MSI y MMII (>derecho) (4/5), con hipoestesia tactoalgésica en MMII. Se solicitaron pruebas complementarias. **Resultados**: ENG-EMG: MM sensitivo-motora axonal aguda sobreañadida a polineuropatía-miopatía difusa leve. Analítica: eosinofilia (3670/µL), ANCA anti-MPO positivos (>100 UI/mL) e insuficiencia renal ([Cr] 3.5 mg/dL). RM: descartó mielopatía. Biopsia renal: glomenuronefritis focal necrosante. Tras diagnóstico de GPEA, se iniciaron metilprednisolona+ciclofosfamida IV, y posteriormente prednisona+azatioprina VO. Con resolución de la eosinofilia, mejoría de la función renal ([1.75] mg/dL) y la debilidad, persistiendo leve paresia del MSI a los 2 meses. **Discusión/Conclusiones**: La GPAE es la única vasculitis sistémica donde la MM es criterio diagnóstico, ocurriendo más frecuentemente en pacientes ANCA positivos. La proteína catiónica de eosinófilos (ECP) facilitaría la degeneración de las fibras nerviosas. También es frecuente la afectación muscular y puede coexistir o no con la afectación nasal o pulmonary. Ante un paciente con MM y antecedentes de EPOC y poliposis nasal debemos sospechar una GPAE siendo el ENG-EMG fundamental para un diagnóstico y tratamiento precoces.

**Hallazgos ENG y Ecográficos en la Neuropatía Cubital en el Codo**

Pablo González Uriel^1^, Saichy Díaz Chang^2^, Vanessa Rodríguez Mugico^1^, Verónica Ángel^3^, Adrián Peláez Laderas^4^

^1^HM La Esperanza, Santiago de Compostela, España

^2^HM La Rosaleda, Santiago de Compostela, España

^3^HM Santiago de Compostela, Santiago de Compostela, España

^4^Fundación HM Hospitals, Madrid, España

**Introducción y objetivos**: La neuropatía cubital en el codo es la segunda en frecuencia en miembros superiores. Su diagnóstico es clínico, apoyado por la eletroneurografía (ENG) (nivel de evidencia A). En los últimos años se ha extendido el uso de la ecografía (ECO) como complemento diagnóstico (nivel B), en la que el área transversal (AT) del nervio es el parámetro más estudiado. Nuestro objetivo es averiguar el grado de correlación entre ambas pruebas en esta patología. **Material y métodos**: Estudio prospectivo casos y controles en el que se comparan los hallazgos ENG y ECO (AT en el codo en su valor más alto). Se ha estudiado: ENG sensitiva antidrómica a 4º y 5º dedo; ENG motora estimulando en muñeca, bajo codo y sobre codo, con flexión de codo en 90º y 10 cm de distancia. Ecografía con sonda de 12 MHz con la que se ha valorado el AT del nervio cubital desde el tercio distal del brazo hasta el tercio proximal del antebrazo. Se han excluido pacientes con traumatismos o cirugías previas de codo y con enfermedades neuromusculares de base. Estudio estadístico en equipo SPSS 29.0. **Resultados**: Se han estudiado 67 nervios, de 61 pacientes (40 mujeres y 21 hombres), con una edad media de 50,15 años. 33 nervios corresponden a los casos y 34 a los controles. La velocidad de conducción (VC) motora media a través del codo fue de 46,2 m/s y 63,5 m/s, respectivamente. El AT media fue 10,7 y 6,3 mm^2^, respectivamente (límite alto de la normalidad 8,8 mm^2^). No se encontraron diferencias significativas entre edades ni sexos, ni con la estatura o el peso. El coeficiente de correlación mayor reside entre la VC motora a través del codo y el AT, siendo de –0,74. El resto de CC no son significativos. **Discusión/Conclusiones**: La correlación más importante, e inversa, reside entre la VC motora a través del codo y el AT en su valor más alto, lo que sugiere que la ECO puedo ser un adecuado complemento a la ENG en el diagnóstico de la neuropatía cubital en el codo.

**Predictores Electromiográficos del Resultado de la Cirugía de Síndrome de Túnel Carpiano Grave**

Edgar Rivera Vigil^1^, Juan Manuel Escobar-Montalvo^2^, Borja Miñón Fernández^1^

^1^Hospital Universitario General de Villalba, 28400 Madrid, España

^2^Hospital Universitario del Henares, Madrid, España

**Introducción y objetivos**: El síndrome de túnel del carpo (STC) es una neuropatía focal muy prevalente que impacta de forma importante en la calidad de vida y la salud. La cirugía del STC es eficaz habitualmente, pero no está claro si esto ocurre en casos de STC grave/muy graves. El objetivo fue analizar la asociación de diversas medidas electromiográficas (ENG/EMG) con el resultado clínico de la cirugía de STC grave. **Material y métodos**: Estudio observacional descriptivo de pacientes con criterios EMG de STC grave (ausencia de potenciales sensitivos y/o latencia motora distal >7 ms y/o amplitude <2 uV y/o ausencia de potenciales de unidad motora (PUM)). La asociación entre el resultado clínico postquirúrgico (variación en el puntaje de la escala visual analógica y en los criterios clínicos del cuestionario DASH (Disabilities of the Arm, Shoulder and Hand)) y las métricas del ENG/EMG se estudió empleando modelos de regresión logística binaria controlados por edad y sexo. El nivel de significancia fue <0,05 y los análisis se realizaron con el software estadístico R (R Core Team 2023). **Resultados**: Se incluyeron 111 sujetos con STC grave (mujeres = 82|hombres = 29) de una mediana de 68 años. Tras la cirugía 98 (88,3%) mejoraron y 13 (11,7%) no mejoraron clínicamente. No se observó asociación entre el resultado clínico postquirúrgico y los valores latencia motora distal, amplitud ni características de PUM. Sin embargo; se observó una importante asociación entre el resultado postquirúrgico y la ausencia de PUM (OR = 33; *p*-valor < 0,001; CI 95%: 3,55–775,54). **Discusión/Conclusiones**: En nuestra muestra, la mayoría de los pacientes tras la cirugía de STC grave mostraron mejoría clínica. Asimismo, la ausencia de potenciales de unidad motora se asoció de forma importante con la falta de mejoría postquirúrgica.

**Atrofia Muscular Espinal no Asociada a SMN1. A Propósito de dos Casos**

Rosa Sánchez Gutiérrez^1^, Mayrel Aguiar González^1^, M. Ángeles Nieto Martin^1^, Mónica Cano Del Pozo^1^, Pablo García Gutiérrez^1^, Paula Arejola de los Mártires^1^

^1^Hospital Universitario Río Hortega, Valladolid, España

**Introducción y objetivos**: Las atrofias musculares espinales no-5q son un conjunto de entidades hereditarias, clínica y genéticamente heterogéneas secundarias a compromiso de las células del asta anterior de la médula. No están asociadas a deleción del gen de sobrevida de la motoneurona (SMN1) responsable de la forma más conocida de atrofia muscular espinal. La principal característica clínica de estos pacientes es la debilidad muscular, proximal o distal, de forma simétrica o asimétrica de predominio en extremidades superiores o inferiores o generalizada y en ocasiones también puede afectar la musculatura respiratoria. La ENG y EMG son de gran utilidad para apoyar el diagnóstico clínico y los hallazgos característicos son la denervación activa y reinervación crónica en la musculatura explorada y la afectación axonal de los nervios motores. **Material y métodos**: P-1. Varón, 5 meses referido por debilidad distal y retraso de crecimiento de 1 mes de evolución. P-2. Mujer, 6 meses derivada por hipotonia de 1 mes de en ambos pacientes se realizó ENG y EMG de extremidades superiores e inferiores. **Resultados**: Los hallazgos fueron: P-1. Conducción motora de peroneal, tibial, mediano y cubital bilateral muestra amplitudes muy bajas con sensitivas normales, denervación activa en deltoides, APB, cuádriceps y ambos tibiales así como potenciales neurogénicos. P-2. afectación axonal motora severa sin respuestas motoras en ambos peroneales y tibiales, con potenciales de baja amplitud en mediano y cubital, objetivando denervación en APB y en ambos tibiales, no evidenciando denervación en cuádriceps ni deltoides, los potenciales de unidad motora son neurogénicos. Ambos pacientes son portadores de heterocigosis en el gen *IGHMBP2* en relación con SMARD1. **Discusión/Conclusiones**: Se dispone de métodos diagnósticos como los estudios neurofisiológicos que han favorecido que muchos trastornos de motoneuronas hayan sido identificados posibilitando un diagnóstico y pronóstico precoz permitiéndonos anticiparnos a las complicaciones.

**Hallazgos EMG Relacionados con Inhibidores de Puntos de Control Inmunitario (Checkpoint Inhibitors)**

M. Elena Lainez Samper^1^, Margarida Gratacòs-Viñola^1^, José Luis Seoane^1^, Ernesto Trallero-Arguás^1^, María Roca-Herrera^1^, Raúl Juntas^1^, Nuria Raguer^1^

^1^Hospital Universitario Vall d’Hebron, Barcelona, España

**Introducción y objetivos**: Los inhibidores de puntos de control inmunitario (ICIs, siglas en inglés) se utilizan para tratar múltiples neoplasias, iniciando la inmunidad antitumoral y bloqueando proteínas citotóxicas señalizadas por inhibidores inmunitarios. Los eventos adversos inmunológicos neurológicos (EAN) son raros, aunque potencialmente graves. Pueden afectar al sistema nervioso periférico (SNP) o central, siendo los trastornos neuromusculares los más comunes. El objetivo del estudio es describir los hallazgos neurofisiológicos en los trastornos neuromusculares relacionados con ICIs (monoterapia o combinación) en 38 pacientes. **Material y métodos**: Estudio retrospectivo de pacientes remitidos para evaluación electrodiagnóstica. Realizamos estudio electrofisiológico con electroneurografia y electromiografía de aguja en los 38 pacientes, complementadas cuando fue necesario con EMG cuantitativo (Turns/Amplitude), Estimulación Nerviosa Repetitiva, EMG de fibra única y Potenciales Evocados Somatosensoriales. **Resultados**: Veintisiete pacientes (71%) mostraron un predominio de signos miógenos, 13 (48%) con actividad espontánea (miositis), 6 (22%) sin actividad espontánea, 6 (22%) con superposición miopatía/disfunción de la unión neuromuscular (UNM) y 2 con EMG normal. Once pacientes (29%) mostraron un predominio de los signos neurógenos, 7 (63%) una polirradiculoneuropatía sensitivo-motora aguda y los otros 4: neuropatía mixta sensitivo-motora, neuropatía sensitiva axonal, mononeuritis múltiple y radiculopatía motora aguda lumbosacra. **Discusión/Conclusiones**: Los EAN más frecuentes en el tratamiento con ICIs son la miositis y neuropatía aguda, así como los trastornos de la UNM, a veces coexistentes. Los exámenes EMG son útiles para una mejor caracterización de los trastornos del SNP y en el diagnóstico diferencial. El reconocimiento temprano y la intervención efectiva pueden reducir la morbilidad y la discapacidad permanente.

**Papel del Estudio Electromiográfico en la Distrofia Facioescapulohumeral: Descripción de Una Serie de Casos Familiar**

Nicole Fontana García^1^, Olga Fedirchyk Tymchuk^1^, Lidia Cabañes Martínez^1^, Marta Villadóniga Zambrano^1^, Paula Ortega García^1^, María Carolina Chinchilla Haylock^1^, Ignacio Regidor^1^

^1^Hospital Universitario Ramón y Cajal, Madrid, España

**Introducción y objetivos**: La distrofia facioescapulohumeral (DFEH) es un tipo de distrofia muscular de base genética y herencia autosómica dominante, que cursa con debilidad progresiva de la musculatura facial y de cinturas, con presentación mayoritariamente asimétrica. **Material y métodos**: Presentamos una serie de seis casos con clínica común de debilidad muscular de inicio focal, que fueron valorados en consultas externas de nuestro hospital entre 2007 y 2024. Por coincidencia en varios apartados de nuestra base de datos, fuimos asociando todos los pacientes a un mismo árbol genealógico a partir de un caso remitido por sospecha de una neuropatía bilateral del nervio accesorio espinal. Cuatro de ellos fueron valorados de manera independiente en nuestra consulta de electromiografía (EMG). El propósito de este trabajo es describir los hallazgos electromiográficos y clínicos de cada uno de ellos. **Resultados**: Todos los casos consultaron por debilidad focal de cintura escapular y/o facial. Se realizó estudio neurofisiológico en 4/6 de los pacientes. El electroneurograma fue normal en todos los casos estudiados. El EMG cuantitativo mostró signos de miopatía y/o de inestabilidad de membrana en los músculos clínicamente sintomáticos, siendo el trapecio derecho el músculo más afectado. El estudio genético confirmó el diagnóstico de DFEH de tipo 1 en 5/6 pacientes. El tiempo medio transcurrido desde el inicio de los síntomas hasta el diagnóstico fue de 16 años. **Discusión/Conclusiones**: En la DFEH el EMG juega un papel muy importante en el diagnóstico. Sin embargo, en la era de la genética, ha pasado a ser una prueba complementaria más. Queremos destacar su valor como herramienta útil en orientar el cuadro clínico y acelerar el proceso diagnóstico. El registro sistemático de los datos de los pacientes en una consulta de EMG puede ayudar a identificar casos familiares aún sin una sospecha inicial de relación de parentesco conocida.

**Doble Potencial en los Síndromes Miasténicos Congénitos: Utilidad en la Aproximación al Diagnóstico**

Gianclaudio Dal Boni Gómez^1^, Paula Carvajal García^1^, Germán Morís de la Tassa^1^, Beatriz Lozano Aragoneses^1^, Maruvic Boruska Ramírez Arvelo^1^, Alejandro Soriano García^1^, Consuelo Valles Antuña^1^

^1^Hospital Universitario Central de Asturias, Oviedo, España

**Introducción y objetivos**: En los estudios de neuroconducciones (ENG), la presencia de un doble potencial o doble pico (“double hump” en inglés) ante un estímulo nervioso único, es característica en los trastornos de la unión neuromuscular (UNM), pudiendo la identificación de éste hallazgo ser de utilidad para orientarnos a un diagnóstico definitivo. **Material y métodos**: Varón de 42 años Estudiado desde la infancia por debilidad del lactante con sospecha de miopatía congénita con estudios previos de imágenes, neurofisiológicos, anatomopatológicos y genética negativos. Es remitido a la unidad neuromuscular de nuestro hospital por persistencia de debilidad generalizada (dificultad para caminar largas distancias e imposibilidad para saltar o correr), sin clínica sensitiva, alteración ocular o bulbar, ni fatigabilidad. Se decide realizar nuevos estudios, incluyéndose una ENG de nervios medianos, cubitales, tibiales posteriores, surales, peroneales motores y sensitivos, junto a una electromiografía (EMG) de la musculatura en la que presentaba clínica durante la visita (cuádriceps y extensor común de los dedos derechos). **Resultados**: El estudio de ENG y EMG tienen como único dato la presencia de un doble potencial en los nervios mediano y cubital. Sospechándose una posible afectación de la UNM, se amplía la prueba añadiendo EMG de fibra aislada (EMGFA o Jitter) y estimulación repetitiva (ER). El resultado de la ER fue normal a nivel distal (Aductor digiti minimi - cubital), pero patológica a nivel proximal (Trapecio - espinal) con un decremento de hasta el 27% en el 4 y 9 potencial. A su vez, la EMGFA presenta una media de 75.4 µs con un 14% de bloqueos asociados. Para finalizar, se completa con un estudio genético de la UNM encontrándose una mutación en el gen *COLQ* (cromosoma 3p25.1) y concluyendo un síndrome miasténico congénito tipo 5. **Discusión/Conclusiones**: La identificación del doble potencial en los nervios motores puede alertarnos de un trastorno de la UNM, dando valor a las pruebas neurofisiológicas para un diagnóstico correcto y temprano.

**Reflejo Bulbocavernoso y Nervio Pudendo. Estudio Observacional en 40 Mujeres**

Andre Miró Andreu^1^, Roberto López Bernabé^1^, Marina Villamor Villarino^1^, Eugenio Barona Giménez^1^, Estefania García Luna^1^, Diana Peñalver Espinosa^1^

^1^Hospital General Universitario Reina Sofía, Murcia, España

**Introducción y objetivos**: La neuralgia del nervio pudendo fue descrita en 1987 por Amarenco. Sin embargo, actualmente sigue siendo una gran desconocida y una patología infradiagnosticada pese a la severa afevtación clínica que se expresa en los niveles S2, S3 y S4. El reflejo bulbocavernoso es una técnica neurofisiológica que nos permite estudiar ambos nervios pudendos mediante la respuesta motora registrada en el musculo bulbocavernoso tras el estímulo eléctrico del clítoris. Describir la relación entre las características clínicas de las pacientes remitidas para estudio del nervio pudendo, y las electroneurográficas del reflejo bulbocavernoso realizadas en todos los casos por el mismo facultativo. **Material y métodos**: Se ha realizado un estudio observacional en 40 mujeres remitidas para el estudio del nervio pudendo al Servicio de Neurofisiología Clínica del Hospital General Universitario Reina Sofía y Morales Meseguer en los últimos 18 meses. **Resultados**: Se observo que el antecedente más frecuente es el parto, seguido de la menopausia. La incontinencia urinaria y el dolor vaginal los síntomas más relatados. Registramos un retraso unilateral de la conducción del reflejo bulvo-cavernoso en 6 de ellas, cuya lateralidad clínica coincide con una latencia distal media de 63,08 ms y una diferencia media interlado de 23 ms; frente a un 4,21 y 2,22 ms respectivamente en los resultados normales. **Discusión/Conclusiones**: La conducción electroneurográfica del reflejo bulbocavernoso es la prueba gold-estándar para el estudio del nervio pudendo, permitiéndonos realizar un diagnóstico diferencial, principalmente con otra patología muy frecuente y de mejor pronóstico como es el dolor miofascial del suelo pélvico. Debido a su compleja realización, la provocación de dolor y necesidad de cooperación de la paciente, así como su difícil interpretación la han convertido en una técnica poco utilizada en comparación con la incidencia de estas patologías en grupos de riesgo.

**Estudio Electromiográfico del Esfínter Periuretral en el Síndrome de Fowler: A Propósito de un Caso**

Olga Fedirchyk Tymchuk^1^, Nicole Fontana García^1^, Lidia Cabañes Martínez^1^, Marta Villadóniga Zambrano^1^, Alberto Artiles Medina^1^, Ignacio Regidor^1^

^1^Hospital Universitario Ramón y Cajal, Madrid, España

**Introducción y objetivos**: El síndrome de Fowler es un trastorno primario de la relajación del esfínter uretral externo (EUE) con la micción que es una causa poco frecuente de retención urinaria en mujeres jóvenes. Fue descrito por primera vez como una serie de casos de pacientes con clínica de retención urinaria, alteraciones específicas en los estudios urodinámicos (EUD) y el hallazgo de descargas repetitivas complejas y/o “decelerating bursts” en el estudio electromiográfico (EMG) de EUE. **Material y métodos**: Describimos un caso clínico de una paciente de 30 años con síntomas de retención urinaria y dificultad para la micción, con EUD no concluyente y normalidad de todas las demás pruebas complementarias, a la que se le solicita un EMG del EUE ante la sospecha de síndrome de Fowler. **Resultados**: Se realiza exploración EMG con aguja concéntrica durante el estudio urodinámico. Durante la fase de llenado se aprecia actividad muscular de forma contínua, así como descargas repetitivas complejas. Su número, duración y frecuencia se acentúan conforme avanza el llenado, siendo máximos justo antes del inicio de la micción. El inicio de la fase de vaciado coincide con una relajación objetiva del EUE, dejando de registrarse las descargas descritas. Se analizaron los potenciales de unidad motora que presentaron parámetros normales, así como ausencia de actividad espontánea de denervación. **Discusión/Conclusiones**: El estudio EMG del EUE es de utilidad en pacientes con sospecha de Síndrome de Fowler, siendo una técnica complementaria a los EUD, sobre todo cuando resultan no concluyentes. Se trata de un síndrome poco frecuente, y aunque haya autores que incluso dudan de su existencia, hemos objetivado los hallazgos EMG descritos en la literatura hasta la fecha. Sin embargo, se desconoce el origen fisiopatológico de estas descargas, por lo que son necesarios más estudios para su mejor caracterización.

**Estudio Neurofisiológico de la Enfermedad de Kennedy: un Caso Clínico Inusual**

Liliam Pérez Bauzá^1^, Jorge De Francisco Moure^1^

^1^Hospital Universitario Miguel Servet, Zaragoza, España

**Introducción y objetivos**: La atrofia muscular bulbo espinal o enfermedad de Kennedy es un trastorno neurodegenerativo progresivo de la neurona motora, heredado a través del cromosoma X y de inicio en la adultez. Su incidencia y prevalencia a nivel mundial son notablemente bajas. Presentamos un caso clínico de dicha enfermedad, resaltando la importancia del estudio neurofisiológico en su diagnóstico. **Material y métodos**: Paciente asintomático de 51 años de edad, derivado por Neurología, por el antecedente del reciente diagnóstico de su hermano de una mutación genética relacionada con una distrofia muscular de cinturas. Se realiza electroneurograma (ENG) de los nervios mediano y cubital (ramas motora y sensitiva), radial sensitivo, sural, peroneo superficial y tibial posterior; explorados de forma bilateral y peroneo profundo izquierdo. Electromiograma (EMG) de musculatura proximal y distal tanto de extremidades superiores como inferiores. **Resultados**: El ENG reveló una disminución de la amplitud de los potenciales de acción sensitivos tanto en extremidades superiores como inferiores, con velocidades de conducción conservadas y sin alteraciones en los estudios de conducción motora. El EMG mostró potenciales de unidad motora de gran amplitud, duración aumentada y polifasia en todos los territorios explorados, con predominio proximal, sin presencia actividad espontánea patológica y con un patrón deficitario global al máximo esfuerzo. Meses después, el paciente comenzó a mostrar síntomas de debilidad con predominio proximal, temblor postural fino, fasciculaciones faciales y en extremidades superiores e inferiores. La clínica del paciente y las alteraciones detectadas en el estudio ENG/EMG llevaron a la sospecha diagnóstica de enfermedad de Kennedy, que fue confirmada posteriormente por un estudio genético. **Discusión/Conclusiones**: Este caso pone de manifiesto la importancia del estudio neurofisiológico como herramienta para la identificación de enfermedades raras como la enfermedad de Kennedy, incluso en pacientes que inicialmente no presentan síntomas.

**Conductancia Electroquímica de la Piel por Sudoscan® en el Diagnóstico de Polineuropatías**

Alberto Solís Martín-Vegue^1^, Carmen García Penco^1^, Elena Urrestarazu Bolumburu^1^, Beatriz Echeveste González^1^, Óscar Manzanilla Zapata^1^, Manuel Alegre Esteban^1^

^1^Clinica Universidad de Navarra, Pamplona, España

**Introducción y objetivos**: La conductancia electroquímica de la piel (ESC) es una prueba no invasiva. Aporta información sobre la función sudomotora, y se relaciona con la densidad de fibras nerviosas simpáticas de las glándulas sudoríparas e intraepidérmicas. Valora la función de las fibras de pequeño calibre mielínicas finas (Aδ) y amielínicas (C) en las polineuropatías de fibra fina y/o autonómicas, así como en aquellas polineuropatías en las que puede coexistir daño de fibras de pequeño y de grueso calibre a lo largo de su evolución. **objetivos**: Valorar la relación que hay entre la ESC, el test sensorial térmico cuantitativo (QTT) y el estudio de conducción nerviosa convencional (ENG), en pacientes que presentan polineuropatía periférica de diferentes etiologías. **Material y métodos**: Revisamos una serie de 30 pacientes remitidos por sospecha de polineuropatía, a los que hemos realizado ESC y/o QTT junto con ENG convencional. Se trata de 18 polineuropatías (6 diabéticas, 6 de fibra fina idiopáticas, 2 inflamatorias crónicas desmielinizantes (CIDP), 4 polineuropatías de etiología no especificada), además de 1 síndrome de Sjögren, 1 disautonomía, 2 amiloidosis familiares, y 8 pacientes con molestias inespecíficas y estudio etiológico negativo. **Resultados**: Observamos 3 patrones diferenciados de afectación: alteración de pruebas de fibra fina (QTT y/o ESC) en 12 pacientes, alteración combinada de gruesa y fina (QTT y/o ESC y ENG) en 9, y sólo de fibra gruesa (ENG) en 4. La polineuropatía diabética muestra similar proporción de los 3 patrones de afectación. Los valores más bajos de ESC se registran en CIDP, en la disautonomía y en un caso grave de amiloidosis. Hay un 64% de QTT con valores patológicos frente al 44% de ESC. **Discusión/Conclusiones**: El Sudoscan® es una prueba no invasiva, que estudia la función sudomotora y ha demostrado su utilidad en el estudio de polineuropatía de fibra fina y/o autonómica. Complementa al estudio de conducción nerviosa convencional, en el diagnóstico fisiopatológico de la polineuropatía periférica.

**Temblor y Mioclonía Ortostáticos Como Causa de Caídas en el Adulto Mayor: Abordaje Diagnóstico y Serie de Casos**

Elena Escario Méndez^1^, Luis Yupanqui Guerra^1^, Adriana Gómez Domínguez^2^, Agustín Querejeta Coma^3^, Carlos Ordás Bandera^3^

^1^Hospital Universitario Rey Juan Carlos, Madrid, España

^2^Hospital Universitario Infanta Elena, Madrid, España

^3^Hospital Universitario Rey Juan Carlos y Hospital Universitario Infanta Elena, Madrid, España

**Introducción y objetivos**: La mioclonía ortostática (MO) y el temblor ortostático (TO) son dos trastornos hipercinéticos del movimiento que aparecen en población anciana, estando su prevalencia infraestimada. El síntoma principal es la aparición de temblor en miembros inferiores al ponerse de pie, produciendo caídas y pérdida de equilibrio. El TO se divide en clásico y lento en función de su frecuencia. La electromiografía de superficie (sEMG) ayuda a su diagnóstico y clasificación ya que los síntomas no suelen ser referidos adecuadamente. El objetivo de este trabajo es exponer una serie de casos, realizar una revisión bibliográfica y describir el estudio sEMG de TO y MO mediante un protocolo sencillo fácilmente aplicable en cualquier laboratorio de electromiografía. **Material y métodos**: estudio observacional descriptivo de una serie de 24 casos registrados entre 2022 y 2024. El estudio de TO y MO se ha realizado mediante sEMG en las extremidades valorando reposo, bipedestación, frecuencia y su comportamiento en maniobras específicas. **Resultados**: La edad media de la muestra fue de 65 años. El principal síntoma referido fue la inestabilidad en la bipedestación, seguido de caídas frecuentes y limitación de las actividades diarias. El estudio electromiográfico de superficie diferenció adecuadamente el TO clásico, siendo la diferencia entre MO y TO lento menos evidente. La frecuencia media de los TO clásicos fue de 15,7 Hz, de los TO lentos 6,6 Hz y de la MO 4,25 Hz. Las maniobras complementarias fueron positivas en un 80% de los TO clásicos siendo este porcentaje menor en los TO lentos. **Discusión/Conclusiones**: El TO y la MO son causa de inestabilidad y caídas en población anciana. Sin embargo, su prevalencia está infraestimada, principalmente por su baja sospecha clínica. El conocimiento de esta patología y de las técnicas electromiográficas diagnósticas, fácilmente aplicables en consulta, resulta importante en países envejecidos como el nuestro, permitiendo un manejo adecuado del paciente.

**Lesiones Yatrogénicas de Nervio Periférico en Una Consulta de Electromiografía**

Lledó Orenga Pachés^1^, José Vicente Orenga Orenga^1^, Silvia Parra Escorihuela^1^, Alina Denisa Ghinea Nuta^1^, Sandra Liliana Beltrán Castro^1^, Claudia Collado Andrés^1^, Nuria Ruiz Montagud^1^

^1^Hospital General Universitari de Castelló, Castelló, España

**Introducción y objetivos**: Las lesiones yatrogénicas de nervio periférico son secundarias a actos médicos o quirúrgicos. La electromiografía (EMG) resulta útil para su diagnóstico y pronóstico. El objetivo es revisar su presentación en consulta de EMG, relacionarlas con las diferentes especialidades, procedimientos y estructuras nerviosas afectadas. **Material y métodos**: Estudio descriptivo retrospectivo de pacientes atendidos en consulta EMG de nuestro servicio en 2022–2023, estudiando los casos compatibles con lesiones yatrogénicas y excluyendo casos secundarios a tóxicos o fármacos. **Resultados**: Se identificaron 86 exploraciones, 1,3% del total de estudios. Pacientes totales 64, a algunos se les realizó más de una EMG. Por sexos 57,8% mujeres y 42,2% hombres, entre 15 y 83 años (media 56,5). Se lesionaron 69 estructuras nerviosas. El 79,7% se identificaron como intraquirúrgicas en relación directa con el campo quirúrgico, el 10,9% intraquirúrgicas sin relación con el campo quirúrgico y se etiquetaron como posturales y el 9,4% secundarias a procedimientos no quirúrgicos. Las especialidades más implicadas fueron cirugía traumatológica y ortopedia 59,4%, neurocirugía 14,1%, cirugía general 7,8% y otorrinolaringología 4,7%. En cuanto a la localización de la cirugía destaca la cadera 15,7% y el raquis lumbosacro 8,7%. De los procedimientos no quirúrgicos destaca la punción de vasos relacionada con radiología 3,1% y cardiología 1,6%. Las estructuras más lesionadas fueron: nervios mediano 14,5%, ciático común 11,6%, facial 11,6% radial 9,4%, espinal 5,8% y femorocutáneo 4,3%; raíces lumbosacras 8,7%. La mayoría fueron de grado grave. **Discusión/Conclusiones**: Las lesiones yatrogénicas de nervio periférico en consulta de EMG son poco frecuentes, presentan un alto grado lesivo en su mayoría y pueden afectar la autonomía funcional del paciente. Las localizaciones más afectadas son las de más complejidad anatómica y con estructuras nerviosas relacionadas, en su mayor parte implicadas en el campo quirúrgico y en las cirugías traumatológicas y ortopédicas.

**El Problema de los Vasa Nervorum**

Eugenio Barona Giménez^1^, Estefanía García Luna^1^, Diana Peñalver Espinosa^1^, Marina Villamor Villarino^1^, Andrea Miró Andreu^1^, Roberto López Bernabé^1^, Francisco de Asís Biec Aleman^1^

^1^Hospital Universitario Reina Sofía de Murcia, Murcia, España

**Introducción y objetivos**: La vasculopatía livedoide (VL) es una dermatosis crónica que provoca microtrombosis en los vasos de la dermis, resultando en dolorosas ulceraciones en los miembros inferiores y neuropatía periférica. Se diferencia de la “vasculitis clásica” al no alterar las paredes vasculares. **Material y métodos**: Mujer de 65 años con antecedentes de vasculitis livedoide, presenta dolor en el muslo izquierdo irradiado al pie desde hace 6 meses. Su último brote de vasculitis fue hace 3 años. Se realiza estudio electromiográfico y neurográfico (EMG-ENG) convencional. **Resultados**: El estudio EMG-ENG reveló ausencia del potencial de acción sensitivo nervioso (PASN) del nervio cutáneo lateral femoral izquierdo, disminución de la amplitud de los PASN de los nervios peroneales superficiales y surales derechos, lentificación de la velocidad de conducción sensitiva (VCS) del nervio mediano derecho y reducción de la amplitud del potencial de acción muscular compuesto (PAMC) del nervio peroneo profundo izquierdo. Se observaron potenciales de unidad motora de características neurógenas con pérdida de unidades motoras en el músculo peroneo lateral largo izquierdo. Los hallazgos descritos cumplen criterios diagnósticos de mononeuritis múltiple según la AANEM. **Discusión/Conclusiones**: La etiología se atribuye a una lesión axonal por oclusión de los vasa nervorum, resultando en neuropatía periférica isquémica sin fenómeno vasculítico clásico. La VL se considera una vasculopatía trombótica oclusiva, asociada a trastornos de coagulación y fibrinólisis. La hipótesis principal sugiere depósitos de fibrina en las paredes vasculares debido a lesión y/o hipercoagulabilidad. La biopsia nerviosa respalda un mecanismo oclusivo con evidencia de trombosis capilar epineural. Este caso pone de manifiesto, la importancia de realizar estudios EMG-ENG para un diagnóstico preciso, ya que la mononeuritis múltiple puede confundirse fácilmente con una polineuropatía.

**Valoración Neurofisiológica de la Disfunción Diafragmática Unilateral en Paciente con Trasplante Cardíaco**

Laura Verna Fierro^1^, Yaiza Guede Guillén^1^, Aiala Saéz Ansotegui^1^, María Cortés Velarde^1^, Guillermo Gonzalez Casaurran^1^, Luis Gabriel Antonio Burgos Bustamante^1^, Pedro José Melgarejo Otálora^1^

^1^Hospital Universitario Gregorio Marañón, Madrid, España

**Introducción y objetivos**: Entre las causas de lesiones unilaterales del nervio frénico se encuentran las complicaciones postquirúrgicas, especialmente en cirugías cardiotorácicas. Estas lesiones afectan a la capacidad respiratoria del paciente, precisando en ocasiones soporte ventilatorio. Parte del diagnóstico y seguimiento involucra la valoración de la función diafragmática a través del electroneurograma (ENG) y el electromiograma (EMG), pudiendo contribuir en las decisiones terapéuticas. **Material y métodos**: Presentamos el caso de un varón de 19 años, con antecedentes de cardiopatía congénita compleja que ha precisado múltiples intervenciones, que evolucionó a insuficiencia cardiaca avanzada y requirió finalmente trasplante cardíaco. Tras el trasplante, se sospecha lesión diafragmática derecha, presentando una insuficiencia ventilatoria con necesidad de soporte continuado con BIPAP. Realizamos en dos ocasiones estudio neurofisiológico EMG/ENG en hemidiafragma derecho. La primera, al mes de trasplante para confirmación diagnóstica, y la segunda a los tres meses para valoración pronóstica. **Resultados**: Los resultados fueron los siguientes: Al mes: ENG: respuesta presente, pero de baja amplitud y latencia aumentada. EMG guiado con ecografía: presencia de potenciales de unidad motora (PUMs) con baja amplitud y corta duración, así como un reclutamiento reducido. No se observaron signos de denervación activa ni cambios neurogénicos crónicos. A los 3 meses: ENG: respuesta presente, discreta mayor amplitud y latencia aumentada. EMG guiado con ecografía: presencia de PUMs de características neurogénicas, con reclutamiento, aunque disminuido, levemente mejor a previo. **Discusión/Conclusiones**: Ante la sospecha de lesión nerviosa periférica, los hallazgos ENG/EMG son útiles tanto en el diagnóstico como en el seguimiento. En nuestro caso, la confirmación diagnóstica y buena evolución posterior de los resultados, permitió una estrategia conservadora en un paciente que presentaba un riesgo quirúrgico elevado ante la posibilidad de una reintervención.

**Revisión de Casos de Enfermedades de Motoneurona Tipo ELA en Nuestra Unidad de los Últimos Años**

Ian Mingote Madrid^1^, Enrique Montes Latorre^1^, Francisco José Palomar Simón^1^, Alicia Silva Cátedra^1^, Catalina Liñan Maho^1^, Mercedes Pujol Congregado^1^

^1^Hospital Universitario Virgen del Rocío, Sevilla, España

**Introducción y objetivos**: Presentar una revisión de los casos de enfermedades de motoneurona tipo Esclerosis Lateral Amiotrófica (ELA) en nuestro centro que han sido diagnosticados en los últimos años y sus correlatos clínico/neurofisiológicos, tanto al inicio de la enfermedad como durante la evolución de la misma. **Material y métodos**: Se han analizado todos los casos de ELA diagnosticados en este centro desde 2019 hasta 2024, englobando un total de 184 pacientes. El rango de edades comprende desde los 21 hasta los 91 años. Se les realizó estudio neurofisiológico ENG (conducciones sensitivas y motoras de MMSS y MMII) y EMG de al menos tres territorios (lumbosacro, cervical y bulbar) y en algunos casos torácico, recogiéndose actividad espontánea registrada (fibrilaciones, ondas positivas, fasciculaciones). Como criterio diagnóstico clínico, se utilizaron los criterios de El Escorial modificados. **Resultados**: Los estudio neurofisiológicos mostraron en la mayoría de los pacientes normalidad de las conducciones sensitivas, salvo 5 pacientes que presentaban una polineuropatía sensitivo-axonal asociada. Las conducciones motoras fueron normales o mostraban amplitudes disminuidas. La EMG presentó alteraciones en todos salvo 2 pacientes, mostrando actividad espontánea y trazados neurógenos en el resto. El territorio que con más frecuencia mostró alteraciones fue el lumbosacro, seguido del cervical, bulbar y torácico. En un número significativo de casos, la EMG permitió localizar signos de afectación de 2ª motoneurona subclínicos, lo que permitió alcanzar o apoyar el diagnóstico de ELA. **Discusión/Conclusiones**: La ELA es una enfermedad neurodegenerativa que, a día de hoy, aún no dispone de un tratamiento curativo. A pesar de ello, existen opciones terapéuticas que permiten mejorar la calidad de vida de estos pacientes, por lo que es fundamental un diagnóstico certero y precoz de esta patología, así como descartar otras enfermedades o alteraciones que simulen clínica de ELA. Para ello, la electromiografía sigue siendo junto con la exploración clínica y resto de pruebas un pilar fundamental que permite corroborar o descartar una posible afectación de 2ª motoneurona en ELA.

**Afectación de Motoneurona en Paciente con Síndrome Canvas. A Propósito de un Caso**

Cristina Llorente Rubio^1^, Alberto Galván Jurado^1^, Beatriz Romero Neva^1^, Alicia Navarro Vicente^1^, Amelia Pita Sanchez^1^, Pedro Jose Orizaola Balaguer^1^

^1^Hospital Universitario Marqués de Valdecilla, Santander, España

**Introducción y objetivos**: CANVAS (Cerebellar Ataxia, Neuropathy, Vestibular Areflexia Syndrome) es un proceso neurodegenerativo caracterizado por ataxia cerebelosa, hipofunción vestibular y neuropatía sensitive. La afectación de la neurona motora ha sido rara vez descrita en la bibliografía. Presentamos un caso de Canvas con participación de 1º y 2º motoneurona. **Material y métodos**: Mujer de 59 años, que presenta un cuadro progresivo de 5 años de evolución de inestabilidad de la marcha, dolor neuropático e hipoestesia “en calcetín”. En los últimos meses añade disartria, sialorrea y trastornos de la deglución. En la exploración física se observa marcha atáxica, nistagmo horizontal con seguimiento ocular sacádico y dismetría apendicular junto con amiotrofia lingual con fasciculaciones y reflejos miotáticos exaltados. Resonancia magnética cerebral con atrofia cerebelosa (vermiana) y test HIT (head impulse test) positivo. **Resultados**: La electroneurografía (ENG) sensitiva evidencia ausencia de potenciales de acción de nervio sensitivo (PANS) en miembros superiores (MMSS) e inferiores (MMII). Electroneurografía motora normal. La Electromiografía (EMG) muestra potenciales de unidad motora (PUM) de amplitud y duración aumentada y potenciales de fasciculación de distribución difusa. Ausencia de potenciales evocados motores (PEM) a la estimulación magnética transcraneal en MMII. En la biopsia de nervio se observa fibrosis perineural y pérdida de fibras nerviosas, sin infiltrados inflamatorios o amiloide. El estudio genético revela doble expansión del alelo AAGGG en el gen *RFC1*, apoyando el diagnóstico de CANVAS. **Discusión/Conclusiones**: La sospecha diagnóstica de CANVAS se basa en la evidencia de axonopatía sensitiva, atrofia vermiana en neuroimagen y exclusión de otras ataxias hereditarias. La afectación simultánea de neurona motora ha sido escasamente descrita. El estudio neurofisiológico contribuye notablemente al diagnóstico permitiendo detectar patologías asociadas para así lograr un manejo terapéutico adecuado.

**Importante Papel del Sistema Nervioso Simpático (SNS) en la Vejiga Hiperactiva Idiopática (VHI). Protocolo de Estudio**

Gabriela Goizueta-San Martín^1^, Gabriela Goizueta-San Martín^1^, Giraldo Ruiz Rodríguez^2^, María Eugenia Rivera Martinez^2^, Susana Martín Albarrán^2^, Irene Serrano^2^, Mercedes González Hidalgo^2^

^1^Hospital Santa Cristina, Madrid, España

^2^Hospital Clínico San Carlos, Madrid, España

**Introducción y objetivos**: La Sociedad Internacional de Continencia (ICS) define la Vejiga Hiperactiva Idiopática (VHI). como urgencia urinaria, habitualmente acompañada de frecuencia y nicturia, con o sin incontinencia urinaria de urgencia, en ausencia de infección del tracto urinario o de otras patologías. Es una afección prevalente, con un impacto significativo en muchos aspectos de la calidad de vida de los pacientes y se asocia con un aumento de la morbilidad. Confirmar la Sensibilización del Sistema Nervioso Central y el importante papel del SNS mediante la Respuesta Simpático-Cutánea (RSC) en la génesis de esta enfermedad. **Material y métodos**: Consentimiento informado en todos los pacientes y aprobación del Comité Ético del Hospital. Se estudia la RSC en 100 sujetos control. (Grupo 0) y 79 pacientes con VHI, de los cuales se excluyeron 25 por presentar, además, otras patologías que podían alterar la RSC, quedando 54 (Grupo 1). Se hace también estudio de pacientes con VHI y Migraña (11 pacientes), como comorbilidad importante asociada (13,9%) (Grupo 2). En todos los grupos se realiza estudio estadístico, analizando parámetros de latencia (s), amplitud (mV) y persistencia (%) de la RSC; con comparación entre ellos y en ambos lados. Se describe protocolo de estudio. No hay conflicto de intereses. **Resultados**: El Estudio realizado muestra diferencias estadísticamente significativas de la RSC entre el grupo de VHI y Control, en el grupo de Migraña y Control, así como en el de VHI y Migraña. No existen variaciones significativas en cuanto a edad y sexo. **Discusión/Conclusiones**: El estudio realizado confirma una hiperactividad del SNS en los pacientes con VHI, en relación con los controles sanos. Por el contrario, los pacientes con VHI y Migraña muestran una hipofunción del mismo (ambas significativas). La RSC es una prueba objetiva y fiable. Puede utilizarse como un biomarcador para el diagnóstico, para evaluar la eficacia de los tratamientos utilizados en la VHI y quizás para emplear otros nuevos.

**Movimientos Anormales en Músicos Profesionales: Hallazgos Electromiográficos**

Laura Verna Fierro^1^, Sonia Ys Rodríguez^1^, Luis Gabriel Burgos Bustamante^1^, Aiala Saéz Ansotegui^1^, Yaiza Guede Guillén^1^, María Cortés Velarde^1^, Natalia Bravo Quelle^1^

^1^Hospital Universitario Gregorio Marañón, Madrid, España

**Introducción y objetivos**: El trastorno del movimiento más frecuente en los músicos es la distonía focal tarea-especifica (FDTS), caracterizada por contracciones musculares sostenidas o intermitentes y posturas anormales al tocar el instrumento. Entre otros trastornos menos frecuentes, se ha descrito el temblor tarea-específica (TST), inducido o no por determinadas posturas. Presentamos dos casos de guitarristas, con hallazgos EMG compatibles con TST y FDTS. **Material y métodos**: Caso 1: Varón de 35 años, con antecedentes de tromboembolismo pulmonar bilateral en tratamiento con anticoagulantes orales, presenta temblor en mano derecha al coger la púa de meses de evolución. Caso 2: Varón de 30 años remitido por sospecha de distonía focal ocupacional en mano derecha de 2 años de evolución y posible neuropatía cubital. Se realizó electromiograma politópico (EMGp) con registro mediante electrodos de aguja subcutáneos en musculatura flexo-extensora de miembro superior derecho, durante tareas o posturas concretas y/o al tocar el instrumento. Se completo estudio con electroneurograma sensitivo y motor (ENG-SM) de nervio cubital derecho en el segundo caso. **Resultados**: El EMGp mostró: Caso 1: Temblor con la postura específica de dedos índice y pulgar al coger la púa o simulándolo, que se registra de forma sincrónica en flexo-extensores de los dedos a unos 5–6 Hz. Caso 2: Con secuencia específica de los dedos durante arpegio o rasgueo de cuerdas, contracciones musculares mal definidas en músculos flexo-extensores de los dedos, con cocontracción ocasional entre antagonistas y, clínicamente, tendencia a la flexión más mantenida del cuarto dedo y extensión del tercero. El ENG-SM de nervío cubital derecho no mostró alteraciones. **Discusión/Conclusiones**: La FDTS y el TST interfieren negativamente e incluso impiden el desempeño ocupacional en los músicos profesionales. El EMGp permite el diagnóstico diferencial con otros trastornos musculo-esqueléticos y ayuda a individualizar la intervención terapéutica mediante toxina botulínica y fisioterapia.

**El Estudio Neurofisiológico en el Botulismo Iatrogénico**

Shijia Li Chen^1^, Jorge de Francisco Moure^1^, Carmen Almárcegui Lafita^1^, Ángela Sánchez-Luis Jiménez^1^, Laura Ramos Barrau^1^

^1^Hospital Universitario Miguel Servet, Zaragoza, España

**Introducción y objetivos**: El botulismo es una enfermedad que produce disfunción de la unión neuromuscular causada por la toxina botulínica, producida por la bacteria *Clostridium botulinum*. Aunque suele asociarse al consumo de alimentos procesados incorrectamente, el botulismo iatrogénico se ha convertido en una preocupación creciente por el aumento de su para fines estéticos y terapéuticos. Los casos descritos están mayoritariamente asociados a la toxina botulínica tipo A. **Material y métodos**: Presentamos el caso de una mujer de 39 años que recibió 200 UI de onabotulinumtoxinA en gastrocnemios con fines estéticos. A los 4 días debutó con diplopía, mareo y debilidad de musculatura cervical. A los 10 días, acudió por 3ª vez a Urgencias presentando además debilidad generalizada, ptosis palpebral, visión borrosa, somnolencia, cefalea, disfagia, estreñimiento, retención urinaria, sequedad de mucosas, inestabilidad de la marcha y leve disartria. Dada la progresión neurológica, se decidió ingreso en UCI y administración de antitoxina botulínica. Se realizaron estudios de electroneurografía, electromiografía, estimulación repetitiva y jitter. Se hizo una revisión de la literature. **Resultados**: Las pruebas complementarias (ECG, análisis de sangre y orina, Rx tórax, RM cerebral y pruebas inmunológicas) fueron normales y la determinación de toxina en suero fue no concluyente. El día 2 de ingreso se realizó estudio neurofisiológico, siendo normal. Tras mejoría clínica, el día 3 de ingreso fue trasladada a planta. El día 8 se repitió el estudio neurofisiológico sin cambios y fue dada de alta tras 21 días de ingreso. **Discusión/Conclusiones**: El botulismo iatrogénico es infrecuente y su diagnóstico se basa principalmente en anamnesis y exploración, pudiendo apoyarse en pruebas de laboratorio y neurofisiológicas. Aunque el estudio neurofisiológico tiene una alta sensibilidad, casos leves o tratados con antitoxina pueden mostrar resultados normales. Las pruebas negativas no lo descartan y el tratamiento no debe retrasarse en pacientes sintomáticos.

**Discrepancias Entre el Estudio Neurofisiológico y Ecográfico en el Diagnóstico del Subtipo de Síndrome De Guillain Barré**

Cristina Llorente Rubio^1^, Beatriz Romero Neva^1^, Alberto Galván Jurado^1^, Alicia Navarro Vicente^1^, Alba Juárez Turégano^1^, Pedro José Orizaola Balaguer^1^

^1^Hospital Universitario Marqués de Valdecilla, Santander, España

**Introducción y objetivos**: Los estudios neurofisiológicos (NFS) son una herramienta fundamental en el diagnóstico de trastornos del sistema nervioso periférico. Recientemente se han introducido nuevos métodos diagnósticos, especialmente estudios ecográficos, como complemento o sustitutivo de la exploración neurofisiológica. Actualmente, se utiliza con frecuencia en la evaluación del Síndrome de Guillain-Barré (SGB). No obstante, la diferenciación de la naturaleza fisiopatológica (axonal o desmielinizante) puede generar discrepancias entre ambos. **Material y métodos**: Presentamos 4 casos de SGB en los que se realizó estudio NFS seguido de ecografía. El estudio NFS incluyó parámetros de conducción motora (latencia distal, amplitud, velocidad, duración y morfología del CMAP, onda F y reflejo H) y conducción sensitiva (velocidad, amplitud del SNAP) en nervios de las cuatro extremidades. Se realizaron estudios evolutivos tomando como referencia el que permitía una clasificación de la variante. Se consideró como subtipo desmielinizante o axonal según criterios de Hadden *et al*. (1998). Asimismo, se categorizaron las variantes según criterios ecográficos evaluados por una radióloga experta. **Resultados**: En los casos 1 y 2 el diagnóstico NFS fue de polirradiculoneuropatía aguda de predominio motor desmielinizante (AIDP); en el caso 3 de polirradiculoneuropatía aguda motora axonal (AMAN); el caso 4 se consideró equívoco. En el estudio ecográfico, los casos 1–2 se clasificaron como variante axonal; el caso 3 se etiquetó de variante desmielinizante; el caso 4 se catalogó de variante desmielinizante. Los anticuerpos antigangliósido fueron positivos en el caso 3 y negativos en los otros casos. **Discusión/Conclusiones**: La ecografía ha adquirido una gran relevancia en el diagnóstico de los trastornos del sistema nervioso periférico. Sin embargo, hay casos en los que existen discrepancias con los hallazgos de las pruebas NFS. Son necesarios más estudios que correlacionen ambos resultados y así valorar la utilidad diagnóstica de la ecografía en el SGB.

**Miastenia Gravis con Clínica Bulbar y Estimulación Repetitiva. Caso Clínico**

Jon Ochoa Iriarte^1^, Imanol Lambarrri San Martin^1^

^1^Hospital Universitario de Cruces, Barakaldo, España

**Introducción y objetivos**: La miastenia gravis es una enfermedad autoinmune que afecta la unión neuromuscular, provocando debilidad muscular y fatigabilidad rápida. Este trabajo presenta un caso clínico que ilustra la importancia de la estimulación repetitiva en el diagnóstico de esta condición. **Material y métodos**: Paciente femenina de 21 años con antecedentes de tabaquismo, obesidad y miastenia gravis IIB tratada con inmunoglobulinas. Síntomas: Fatigabilidad bulbar, voz nasal gangosa, claudicación mandibular, regurgitación nasal de líquidos. Diagnóstico: Se llevo a cabo un estudio de estimulación repetivia (ER) a 3 HZ del nervio facial derecho con registro en músculo nasalis.** Resultados**: La ER reveló un importante derecremento de la amplitud y el área, compatible con un déficit de trasmision neuromuscular. Se observaron anticuerpos anti-Receptor de Acetilcolina (anti-RaC) positivos. Tomografía computarizada (TC) torácica: donde se objetiva un posible timoma (corroborado con anatomía patológica posquirurgica). Intervención: La paciente fue sometida a a una timectomía vía videotoracoscopía asistida por video. La evolución postoperatoria fue favorable con mejoría significativa de los síntomas. **Discusión/Conclusiones**: La estimulación repetitiva es una herramienta diagnóstica valiosa en la miastenia gravis con clínica bulbar. La identificación temprana y precisa es crucial para un manejo efectivo y mejores resultados a largo plazo. La investigación continua y el desarrollo de nuevas terapias son necesarios para mejorar los resultados en pacientes refractarios a los tratamientos convencionales.

**Distrofia Miotónica Tipo 1 y Tipo 2: Hallazgos Neurofisiológicos más Allá de la Electromiografía**

Maruvic Boruska Ramírez Arvelo^1^, Paula Carvajal García^1^, Bárbara Martín Escuer^1^, Eva Rey Pérez^1^, Gianclaudio Dal Boni Gómez^1^, Alejandro José Soriano García^1^, Consuelo Valles Antuña^1^

^1^Hospital Universitario Central de Asturias, Oviedo, España

**Introducción y objetivos**: La distrofia miotónica es una enfermedad multisistémica autosómica dominante en la que predomina la clínica de afectación muscular. La forma más común es la distrofia miotónica tipo 1 (DM1), por defecto en el gen de la proteína quinasa miotonina en el cromosoma 19q13. La distrofia miotónica tipo 2 (DM2), se debe al defecto del gen *CNBP* en el cromosoma 3q. **Material y métodos**: Se presentan los estudios neurofisiológicos realizados: ENG y EMG, a 3 pacientes remitidos a Neurofisiología por sospecha clínica de miopatía. Uno de ellos, aportaba también valoración oftalmológica con diagnóstico de posible distrofia macular por lo que se realizo tambien ERG y PEV. **Resultados**: La neurografía fue normal en los 3. Para el caso 1 se hallaron signos de inestabilidad de membrana con presencia de frecuentes descargas miotónicas, más evidente en musculatura distal de extremidades y facial con potenciales de unidad motora (PUM) de características miopáticas. Al estudiar la vía visual se detectó afectación de la retina externa, más evidente a nivel macular bilateralmente. En el caso 2 se encontraron descargas miotónicas y unos PUMs de características miopáticas algo más evidente en musculatura proximal pero presente tanto a nivel de musculatura distal como proximal en las 4 extremidades. Finalmente, en el caso 3, se obtuvo un patrón miopático simétrico de distribución proximal sin poder aislar ni en reposo ni tras maniobras de activación las descargas miotónicas. Los casos 2 y 3 no tuvieron otras alteraciones más alla de la EMG. Los pacientes fueron positivos en el estudio genético de DM1 en el primer caso y positivo para DM2 en los otros dos. **Discusión/Conclusiones**: Los hallazgos neurofisiológicos en la distrofia miotónica van más allá de la afectación neuromuscular. En estos pacientes es especialmente importante realizar un abordaje neurofisiológico integral con el fin de caracterizar los diferentes fenotipos, optimizar la aproximación diagnóstica y orientar el estudio genético.

**Todos los Síndromes de Tunel del Carpo son Iguales … O no?**

Adrián Baz López^1^, José Luis Relova Quinteiro^1^

^1^Complejo Hospitalario Universitario de Santiago de Compostela, Santiago de Compostela, España

**Introducción y objetivos**: Una de las causa más frecuentes de remisión de un paciente a la consulta de Neurofisiología Clínica es para valorar la existencia de un síndrome del túnel del carpo (STC). Sin embargo, con frecuencia otras etiologías son las responsables de la clínica del paciente. **Material y métodos**: Presentamos el caso de un paciente de 48 años remitido desde atención primaria para descartar un STC. El paciente presenta dolor local sobre la eminencia tenar y parestesias en la cara externa del segundo dedo de la mano derecha. Se realiza un estudio electroneurográfico (ENG) en ambas manos y un estudio eletromiográfico (EMG) en el brazo en cuestión. En el estudio electromiográfico se observa un patrón intermedio con aumento de la duración de los PUM en el APB con normalidad del estudio EMG sobre el Flexor Carpi Radialis y Primer Dorsal Interóseo. **Resultados**: En estudio ENG se observa un aumento de latencia motora del nervio Mediano con un descenso moderado amplitud. Se realiza un estudio de la conducción sensitiva sobre el primer, segundo, tercer y quinto dedos de ambas manos. Además, se realiza un estudio comparativo de la conducción del nervio Mediano sobre el cuarto dedo y sobre el segundo lumbrical e Interóseo que muestra una diferencia de latencias menor de 0,5 ms. **Discusión/Conclusiones**: El STC es una de las patologías más frecuentes remitidas a la consulta de Neurofisiología Clínica, siendo la neuropatía por compresión más frecuente de los miembros superiores. Sin embargo, otros procesos pueden simularlo como la afectación exclusiva de la rama recurrente motora del nervio mediano. La compresión de la rama motora recurrente del nervio mediano, de manera aislada o en conjunción con un STC, es una entidad clínica distinta. La mayoría de los casos descritos de compresión de esta rama se refieren a procesos compresivos locales. Esta infrecuente patología debe ser tenida en cuenta en la realización de los estudios neurofisiológicos.

**Índrome de Dejerine-Sottas de Inicio Temprano, a Propósito de un Caso**

Víctor Hugo Rubio Suárez^1^, María Concepción Maeztu Sardiña^1^, Reetika Mukesh Baharani Baharani^1^, Pilar Rosario Martínez Martínez^1^, Julián Vázquez Lorenzo^1^, Sofía Ortigosa Gómez^1^

^1^Hospital Universitario Virgen de la Arrixac, Murcia, España

**Introducción y objetivos**: La enfermedad de Charcot-Marie-Tooth (CMT) es la forma más común de neuropatía sensitiva y motora hereditaria, el CMT tipo 1 (CMT1) se clasifica como desmielinizante y está causado por distintas mutaciones, uno de los genes alterados es el *MPZ* (*1q22*) y se manifiesta como neuropatía periférica con enlentecimiento progresivo de la conducción nerviosa e hipertrofia de las células de Schwann. El fenotipo es variable dependiendo de la mutación asociada, siendo la de inicio temprano y de padres asintomáticos una forma de presentación poco frecuente, con retraso de la marcha y velocidad de conducción nerviosa motora (VCNM) <10 m/s, conocido habitualmente como síndrome de Dejerine-Sottas. **Material y métodos**: Preescolar de 22 meses concebida mediante fecundación in vitro con gametos propios, y despistaje para enfermedades hereditarias, con desarrollo motor normal hasta los 14 meses; comenzando con bipedestación y deambulación inestable causando múltiples caídas, precisando apoyo constante con los miembros superiores. Además, hipotonía muscular sin atrofias y actitud en genu-recurvatum, se realiza estudio electromiografía convencional (EMG-ENG). **Resultados**: El estudio EMG-ENG muestra ausencia de todos los potenciales sensitivos excepto de nervio mediano derecho con velocidad de conducción sensitiva a 10,2 m/s y ausencia de todos los potenciales de acción muscular compuesto excepto el nervio cubital derecho con velocidad de conducción motora a 6 m/s, siendo sugestivo de polineuropatia desmielinizante sensitivo-motora de grado muy severo. Posteriormente el estudio genético pone de manifiesto una mutación *de novo* tipo delecion de exón 2 del gen *MPZ*, asociado a síndrome de Dejerine-Sottas. **Discusión/Conclusiones**: Ante un retroceso de adquisiciones motoras en un niño, sin antecedentes familiares, se deberá pensar en una polineuropatia desmielinizante genética de inicio precoz. El estudio EMG-ENG orientó al diagnóstico muy poco frecuente, que se confirmó con el estudio genético.

**Trastorno de la Transmisión Neuromuscular Como Efecto Adverso de Tratamiento CAR-T, a Propósito de un Caso**

Víctor Hugo Rubio Suárez^1^, María Concepción Maeztu Sardiña^1^, Reetika Mukesh Baharani Baharani^1^, Pilar Rosario Martínez Martínez^1^, Julián Vázquez Lorenzo^1^, Sofía Ortigosa Gómez^1^

^1^Hospital Universitario Virgen de la Arrixaca, Murcia, España

**Introducción y objetivos**: La inmunoterapia con células T modificadas con receptor quimérico antígeno especifico (CAR-T) emerge como tratamiento para enfermedades hematológicas principalmente neoplasias de células B resistentes a terapias convencionales. El incremento en la supervivencia conlleva efectos adversos inmunomediados, como el síndrome de liberación de citoquinas (SLC) y el síndrome de toxicidad neurológica asociado a la terapia con células inmunoefectoras (ICANS). La encefalopatía y la afasia los síntomas más frecuentes. Como tratamiento se pueden usar medidas de soporte, esteroides y anticonvulsivantes. **Material y métodos**: Paciente diagnosticado de mieloma múltiple, participante en ensayo clínico aleatorizado CARTITUDE-5. Uno de los brazos recibe tratamiento con CAR-T, administrándose el 26/12/23. En enero 2024 presentó SLC. El 16/02/2024 ingresa con ptosis palpebral moderada y limitación en la aducción de ojo izquierdo (OI) que le causa diplopía binocular con la dextroversión, mejorando espontáneamente del OI y empieza con ptosis palpebral de ojo derecho, limitación de la aducción y disfagia. Se realiza estudio electromiografia convencional y de transmisión neuromuscular, Jitter por estimulación axonal de nervio facial y registro con electrodo concéntrico de aguja en músculo orbocularis oculi y estimulación repetitiva. **Resultados**: Se objetiva un valor de jitter medio anormal (MCD 37 µs; normal <27 µs) e incremento del número de potenciales de fibra muscular con jitter individual aumentado (36%; normal <10%), sugestivo de trastorno postsináptico de la transmisión neuromuscular. Además, se objetivó una polineuropatía sensitiva axonal, con afectación de extremidades inferiores y superiores. Se inició tratamiento con corticoides, con optima respuesta y desaparición de los síntomas. **Discusión/Conclusiones**: Se describe el primer caso de miastenia gravis, como efecto adverso en un paciente con mieloma múltiple, participando en un ensayo clínico aleatorizado con CAR-T, lo que sugiere neurotoxicidad no ICANS del fármaco.

**Caracterización de Casos Clínicos de la Ataxia Tipo Canvas**

Jennifer Paola Cantarero Durón^1^, Maria Asunción Martín Santidrián^1^, Fernando Vázquez Sánchez^1^, Alina Havrylenko Vynogradnyk^1^, Lais Alexandra Reinoso Aguirre^1^, Diego Francisco Peña Olaya^1^, Joseba Soto Ibáñez^1^

^1^Hospital Universitario de Burgos, Burgos, España

**Introducción y objetivos**: CANVAS, por sus siglas en inglés, (Cerebellar Ataxia, Neuropathy, Vestibular Areflexia Syndrome) es una entidad inusual, emergente en los últimos 20 años, de curso progresivo y edad media 50–60 años, causada por una aparente pérdida de función del gen *RFC1*, caracterizada por ataxia cerebelosa, neuronopatía sensitiva, arreflexia vestibular y tos crónica. El objetivo es describir los hallazgos clínicos y neurofisiológicos de dos casos de nuestra consulta para alertar de la frecuencia de esta enfermedad y favorecer un diagnóstico precoz. **Material y métodos**: El primer caso clínico, varón de 51 años con antecedente de tos crónica y exfumador. Refería parestesias ocasionales en manos desde hace 2 años, asociando alteración sensitiva en piernas. Tos crónica de 10 años de evolución. EF: Alteración de la sensibilidad vibratoria distal con mínima incoordinación talón – rodilla izquierda. Hipoestesia en calcetín a media pierna algésica. Tandem inestable, no nistagmo. Reflejos miotáticos normales. El segundo caso, mujer de 68 años con antecedente de HTA y tos crónica. Inicia con hipoestesia en pies, que ha ido ascendiendo hasta rodillas asociado a sensación de inestabilidad de la marcha, de 3 años de evolución. EF: sin alteraciones. Reflejos miotáticos conservados. **Resultados**: TC toracoabdominal: normal. ENG sensitivas y motoras, reflejos H, ondas F, blink reflex. Estudio genético: presencia de 2 alelos expandidos AAGGG en el intron del gen* RFC1. ***Discusión/Conclusiones**: CANVAS, se caracteriza por pérdida de neuronas en el ganglio de la raíz dorsal. Suele manifestarse como una neuronopatía sensitiva, aún con reflejos miotáticos conservados. En nuestro caso, la presentación clínica y los rasgos neurofisiológicos nos orientaron a una patología diferente a las neuropatías sensitivas típicas, apoyado posteriormente por estudio genético, pero principalmente sospechar la enfermedad ante una neuropatía sensorial de causa inexplicable.

**Primum non Nocere**

Eugenio Barona Giménez^1^, Estefanía García Luna^1^, Diana Peñalver Espinosa^1^, Marina Villamor Villarino^1^, Andrea Miró Andreu^1^, Roberto López Bernabé^1^, Julia Moreno Candel^1^

^1^Hospital Reina Sofía de Murcia, Murcia, España

**Introducción y objetivos**: Se presenta el caso de una mujer de 67 años, intervenida por síndrome del túnel carpiano (STC) en la mano izquierda, quien fue remitida debido a una mejoría insuficiente. **Material y métodos**: Se realiza anamnesis a la paciente, relata que antes del diagnóstico de STC realizó un movimiento de rotación forzada del brazo, experimentando una contracción muy dolorosa y quedando con el brazo en una posición retorcida durante varios minutos. Posteriormente, comenzó a notar adormecimiento en el primer, segundo y tercer dedos de la mano izquierda, acompañado de pérdida de fuerza. **Resultados**: Tras realizar estudio neurográfico y electromiográfico, donde se observó un retraso de la latencia distal del N. interóseo anterior izquierdo (rama de N. mediano), con registro en pronador cuadrado y abundante actividad espontanea con potenciales de unidad motora de caracteristicas neurógenas en el mismo musculo, se diagnosticó de una lesión del nervio interóseo anterior izquierdo. **Discusión/Conclusiones**: Este caso destaca las consecuencias de un diagnóstico incorrecto. Actualmente, la elevada carga de trabajo en algunos centros puede llevar a pasar por alto la realización de una anamnesis y exploración física adecuadas, limitándose a realizar el estudio orientado por otro especialista. Como consecuencia del diagnóstico erróneo, nuestra paciente tuvo que someterse a una intervención quirúrgica innecesaria. Es crucial que, a pesar de la carga asistencial, recordemos que el nombre de nuestra especialidad también incluye la palabra “clínica”.

**Uso Recreativo de Óxido Nitroso: Una Posible Nueva Amenaza de Polineuropatía en la Población Española**

Lucas McHugh Antonio Lloyd^1^, Ruiz Navarrete Pablo^1^, Ortega León Teresa^1^, Galdón Castillo Alberto^1^

^1^Hospital Universitario Virgen de las Nieves, Granada, España

**Introducción y objetivos**: El óxido nitroso (NO) es un anestésico inhalado que presenta diversas indicaciones médicas. Aunque lleva utilizándose de manera recreativa durante más de dos siglos, el reciente fácil acceso a este producto ha provocado un aumento de su uso recreativo en diversos países. En esta comunicación pretendemos documentar a la comunidad científica acerca de los hallazgos neurofisiológicos derivados del uso recreativo de NO además de hacer alusión a los factores que pueden aumentar el riesgo de padecer patologías asociadas a su uso. **Material y métodos**: Hemos realizado una revisión bibliográfica acerca del uso recreativo de NO, que incluye su prevalencia, los cuadros clínicos más frecuentes y su fisiopatología. Por último, se presentarán una serie de casos de polineuropatías debido al uso recreativo de dicha sustancia. **Resultados**: Aunque en España el NO ocupa el último puesto en el ranking de popularidad de drogas de abuso, con una prevalencia estimada del 0,2%, en Reino Unido éste se coloca en segundo puesto, únicamente por detrás del cannabis. Las polineuropatías causadas por abuso de NO con fines lúdicos se manifiestan como polineuropatías sensitivo-motoras, con predominio distal, de naturaleza desmielinizante, axonal o mixta. El mecanismo patológico es dosis dependiente y parece estar relacionado con la inactivación de la vitamina B12 por acción del NO, lo que interfiere en las reacciones catalizadas por esta vitamina, cruciales en la síntesis de fosfolípidos que forman las vainas de mielina. **Discusión/Conclusiones**: El creciente uso recreativo de NO que se está viendo en otros países en los últimos años, podría marcar una tendencia que podríamos observar en las consultas españolas de EMG en forma de casos de polineuropatía, especialmente en la población joven. Debido al estigma que sufren estos pacientes, sería conveniente incluir el uso recreativo de NO dentro del diagnóstico diferencial de las polineuropatías, especialmente en pacientes jóvenes en los que se hayan descartado otras etiologías más comunes.

**Monitorización Intraoperatoria en Parotidectomías. Experiencia de Cinco Años en un Centro de Tercer Nivel**

Juan Sebastián Aller Álvarez^1^, Trinidad Blanco Hernández^1^, Andrés García Verdú^2^, Ana Piedad Arias Balsalobre^1^, Sara Cors Serra^1^, Enrique Zapater Latorre^1^, José Ramón Alba García^1^

^1^Hospital General de Valencia, Valencia, España

^2^Hospital General Universitario Santa Lucía de Cartagena, Cartagena, España

**Introducción y objetivos**: El papel de la monitorización intraoperatoria (MIO) en las parotidectomías sigue siendo controvertido en los tumores benignos debido a que puede haber una disfunción del nervio facial sin que se registren cambios durante la cirugía. En nuestro centro se realiza desde 2019 y presentamos nuestros resultados. **Material y métodos**: Revisión retrospectiva de 75 pacientes intervenidos con MIO (2 en 2019, 16 en 2020, 17 en 2021, 15 en 2022, 16 en 2023 y 9 hasta marzo de 2024). El protocolo en nuestro centro incluye electromiografía en tiempo real (fEMG) y estimulación de nervio facial con sonda monopolar y registro en musculatura facial (sEMG). En muchos se realizaron potenciales evocados motores corticobulbares (CoMEPs) y desde el año pasado se introdujo el Blink-Reflex. **Resultados**: El rango de edad fue de 23–86 años (76% ≥50 años). 41 mujeres. 41 lado izquierdo. Las patologías fueron: 47% adenoma pleomorfo, 24% tumor de Whartin, 15% neoplasias malignas y 15% otras patologías. En 6 pacientes (8%) con neoplasias malignas se tuvo que sacrificar el tronco o una rama del nervio. En 25 (33%) no hubo déficits ni cambios significativos en la MIO. En 20 (26%) con neoplasias benignas hubo disfunción leve de la rama marginal con paresia mentoniana y con buen pronóstico funcional (20% con disminución en sEMG). En 19 (25%) hubo paresia de otras ramas del nervio facial y con buena recuperación (61% con disminución en sEMG). 5 pacientes (6%) tuvieron parálisis facial severa (100% con disminución en sEMG). Los CoMEPs no fueron útiles en la mayoría de los pacientes y todos los pacientes que conservaron el Blink-Reflex mantuvieron la movilidad del orbicular del ojo. **Discusión/Conclusiones**: La presencia de debilidad facial es un hallazgo frecuente tras una parotidectomía. En nuestra serie, la MIO no fue muy eficaz detectando disfunciones de la rama marginal del nervio facial y que suelen tener buen pronóstico funcional, pero si fue útil en la localización de las ramas del nervio y en la detección de lesiones más proximales y con peor pronóstico.

**Monitorización Intraoperatoria Cirugía Vascular (TEVAR). A Propósito de un Caso**

Inés Vicente Garza^1^, Beatriz Montero^1^, Marta Arias Ávarez^1^, Anna Bonet^1^, Sonia Navarrete Navarro^1^

^1^Hospital Clínico Universitario Lozano Blesa, 50009 Zaragoza, España

**Introducción y objetivos**: Este caso clínico tiene como objetivo destacar la importancia de la MIO en cirugías de procesos vasculares. Su finalidad es prevenir la isquemia cerebral, medular y periférica, y evitar así los daños irreversibles del sistema nervioso. **Material y métodos**: Presentamos un caso de un paciente de 74 años con aneurisma en arco aórtico y aorta torácica descendente que ingresa en HCU Lozano Blesa para una cirugía programada de endoprótesis de arco aórtico (TEVAR). Se realizó la cirugía en dos tiempos, siendo este caso correspondiente al segundo tiempo. Durante la intervención se realizó MIO aplicando diversas técnicas: potenciales evocados motores transcraneales (t-PEM) con estimulación slowMEP 5 trenes, y recogida en músculos deltoides, ECD, APB, vasto lateral, TA y AH bilaterales; y somatosensoriales (t-PESS) de ambos nervios medianos y tibiales posteriores; EEG superficial según sistema 10/20 (Fz, Cz, F3, C3, T3, P3, O1, F4, C4, T4, P4 y O2), electroencefalograma (EEG) espectral (CDSA), y EMG de barrido libre. **Resultados**: - Aumento de latencia de la P37 al estimular nervio tibial posterior derecho derivado de la isquemia generada por el inductor en arteria femoral derecha, que se mantiene hasta el final de la cirugía. - Durante el clampaje de arteria carótida derecha se registra en EEG, tras 3 minutos, aplanamiento de la actividad cerebral en hemisferio derecho y asimetría en EEG espectral, avisando al cirujano y quitando el clampaje de forma inmediata, recuperando la actividad eléctrica cerebral. Se realizan t-PEM observando ausencia de potenciales motores en todo hemicuerpo izquierdo, que recuperan completamente tras una hora. **Discusión/Conclusiones**: La MIO en cirugía vascular es una técnica de gran valor ya que puede detectar los cambios en diferentes estructuras nerviosas en tiempo real, pudiendo prevenir la irreversibilidad de las complicaciones intraoperatorias derivadas de la isquemia.

**Rigidez de Columna y Cambios Neurofisiológicos en Escoliosis: Estudio Retrospectivo y Multidisciplinar en el HUGCDN**

Inmaculada Rodriguez Ulecia^1^, Amanda Labrador Rodríguez^2^, Octavio Jiménez Vega^2^, Ricardo Navarro Navarro^2^, Laura Pastor Martín^2^, Tito Fernandez Varela^2^, Arturo Montesdeoca Ara^2^

^1^Hospital Universitario de Gran Canaria, Las Palmas de Gran Canaria, España

^2^Hospital Universitario de Gran Canaria Doctor Negrín, Las Palmas de Gran Canaria, España

**Introducción y objetivos**: Durante la cirugía de escoliosis puede haber lesiones neurológicas por noxas mecánicas y medulares. La realización de monitorización intraoperatoria (MIO) permite prevenir un déficit de manera precoz para poder realizar maniobras de corrección y evitar en lo posible una lesión neurológica postquirúrgica. Además del propio acto quirúrgico, existen circunstancias del propio paciente como el grado de curvatura a corregir, el bending o rigidez de la columna, así como otras inherentes al acto quirúrgico entre las que destacan el tiempo, el sangrado y el protocolo anestésico. **Material y métodos**: Dada la necesidad de una correcta comunicación multidisciplinar, se realiza un estudio retrospectivo observacional entre los años 2019 y 2023 en el HUDGCDN, en el que se analiza los pacientes intervenidos de cirugía correctiva de escoliosis con MIO. **Resultados**: Como resultados de nuestro estudio, se objetiva que el aumento de señales de alarma en monitorización mediante PESS y PEM se asocia a mayor rigidez de columna. En nuestra muestra el 1,5% presentó un déficit permanente y el 12,6% un déficit transitorio. Las señales de alarma aparecieron en un 22,9% del total (correspondiéndose 97,8% con columnas rígidas), manteniéndose sin déficit postquirúrgico un 10,3% al cambiar la estrategia debido a nuestro aviso, siendo los PEM los que presentaron una mayor sensibilidad. **Discusión/Conclusiones**: En conclusión, el estudio proporciona información valiosa sobre los factores de riesgo y la sensibilidad de la monitorización intraoperatoria multimodal en la cirugía de escoliosis. Estos hallazgos contribuyen a mejorar la comprensión de la enfermedad y a optimizar el manejo quirúrgico de los pacientes con escoliosis tanto en el acto quirúrgico como en la planificación, predicción de posibles alteraciones durante el mismo, análisis de los eventos de la MIO y resultados de la cirugía. Todo ello conduce a una práctica clínica más efectiva y segura.

**Electrocorticografía Intraoperatoria en Cirugía de la Epilepsia. Eficacia y Relación con Hallazgos Anatomopatológicos**

Alba González Arjonilla^1^, Matilde Velasco Mérida^1^, María José Postigo Pozo^1^, Guillermo Ibáñez Botella^1^, Victoria Fernández Sánchez^1^

^1^Hospital Regional Universitario de Málaga, Málaga, España

**Introducción y objetivos**: Se ha demostrado que la cirugía posee una gran eficacia en comparación con el manejo farmacológico en pacientes con epilepsia farmacorresistente. Sin embargo, entre el 30–50% de los intervenidos presentan recurrencia de las crisis. Normalmente, esto se debe a una resección incompleta de la zona epileptogénica. La electrocorticografía intraoperatoria (iECoG) consiste en la colocación de electrodos directamente en la corteza cerebral antes y/o después de la resección del foco para la detección de descargas interictales epileptiformes (DIE) más allá de la lesión. **Material y métodos**: Se ha realizado un estudio descriptivo retrospectivo de los pacientes en los que se ha llevado a cabo iECoG entre 2019 y 2024. Se han revisado los hallazgos del registro durante la intervención, los resultados del estudio anatomopatológico (AP) y la evolución de los pacientes. **Resultados**: Se presentan 8 sujetos (2 mujeres, 7 hombres) de entre 13 y 62 años diagnosticados de epilepsia focal sintomática farmacorresistente e intervenidos mediante craneotomía y exéresis de la lesión, controlada por iECoG. Se registraron DIE en electrodos adyacentes a la lesión (que desparecieron tras la resección), en todos ellos excepto en uno (el único que no ha presentado mejoría de las crisis). Entre los hallazgos AP destacan las malformaciones vasculares y las displasias corticales. **Discusión/Conclusiones**: Gracias a la cirugía resectiva y la utilización de la iECoG, todos los pacientes (excepto uno, en el que no se detectaron DIE en ningún momento) han alcanzado la libertad de crisis. Sin embargo, por el momento, no ha sido posible la retirada total del tratamiento farmacológico en ninguno de ellos, siendo necesaria la biterapia en la mayoría. En conclusión, la iECoG constituye una herramienta de gran valor en la cirugía resectiva, permite definir los márgenes del tejido epileptogénico perilesional y, en nuestra experiencia, mejorar el pronóstico de los pacientes consiguiendo la libertad de crisis.

**Hemiparesia Derecha Severa Postquirúrgica con MNIO**

Diana Peñalver Espinosa^1^, Eugenio Barona Giménez^1^, Estefanía García Luna^1^, Marina Villamor Villarino^1^, Andrea Miró Andreu^1^, Roberto López Bernabé^1^, José Félix Jaulín Plana^2^

^1^Hospital General Universitario Reina Sofía, Murcia, España

^2^Hospital Clínico Universitario Virgen de la Arrixaca, Murcia, España

**Introducción y objetivos**: El síndrome del área motora suplementaria (AMS) se caracteriza por acinesia transitoria y global de predominio contralateral con alteración del lenguaje postquirúrgico. El objetivo de este caso es conocer la existencia de este síndrome transitorio. **Material y métodos**: Varón de 37 años que consulta por episodios paroxísticos y autolimitados de movimientos tónico-clónicos secundarios a una lesión ocupante de espacio en AMS izquierda compatible con tumor glial de bajo grado. Se realizó la exéresis completa del tumor con monitorización neurofisiológica intraoperatoria (MNIO) consistente en mapeo funcional con estimulación monopolar, estimulación directa, potenciales evocados motores (PEM) corticobulbares transcraneales y PEM corticoespinales con estímulo transcraneal, todos ellos estables durante la intervención. **Resultados**: Tras una resección completa del tumor con una MNIO sin incidencias, el paciente presentó una hemiparesia derecha que mejoró tras 7 días, siendo diagnosticado de síndrome del AMS. **Discusión/Conclusiones**: La MNIO es una técnica intraoperatoria básica para la prevención de secuelas neurológicas post-quirúrgicas, pero debemos conocer aquellas entidades transitorias que asocian clínica neurológica en el post-opeatorio inmediato tras una MNIO sin incidencias.

**Reproducibilidad y Seguridad del Electrodo Saxophone en la Monitorización Continua del Nervio Facial en Parotidectomías**

Estela Lladó-Carbó^1^, Raidili Cristina Mateo Montero^1^, Vanessa Zerpa Zerpa^2^, Massiel Cepeda Uceta^2^, Manuel Paez Romero^2^, Iván Domènech Juan^2^

^1^NEUROTOC, Barcelona, España

^2^AMIQ, Barcelona, España

**Introducción y objetivos**: Una de las principales complicaciones neurológicas durante la cirugía de resección de la glándula parótida es la parálisis del nervio facial con incidencias que varían del 28,8 al 72,2%. **Objetivo**: Con el objetivo de minimizar la incidencia de esta complicación presentamos el uso de la MIO continua del nervio facial mediante el uso del electrodo Saxophone, estudiando su reproducibilidad y seguridad cuando es aplicado intraoperatoriamente, como técnica complementaria al mapeo periférico del nervio facial. **Material y métodos**: De marzo del 2023 a marzo del 2024 se realiza la MIO continua del nervio facial en 10 pacientes sometidos a parotidectomía primaria. Todos los pacientes presentaban una función del nervio facial normal House-Brackmann 1. El protocolo de MIO incluyó electroencefalograma (EEG) basal, EMG de barrido libre para las 5 ramas del nervio facial (frontalis, oculi, nasalis, oris, mentalis), mapeo periférico del nervio facial con estimulador monopolar y la monitorización continua del nervio facial con electrodo de estimulación Saxophone. **Resultados**: La MIO continua mediante registro de CMAP para las 5 ramas del nervio facial fue reproducible en el 100% de los casos (10/10). En uno de los casos se observó una caída en la amplitud del CMAP para rama mentalis, presentando un déficit transitorio postoperatorio en correlación con los hallazgos neurofisiológicos intraoperatorios. No se observaron signos de neuroapraxia durante la colocación del electrodo Saxophone así como durante la estimulación con intensidades de hasta 1,3 mA, en ninguna de las 50 ramas monitorizadas en total. **Discusión/Conclusiones**: Se demuestra que la MIO continua del nervio facial mediante el uso del electrodo en Saxophone es un método seguro durante las parotidectomías en nuestra serie de casos. Las respuestas evocadas son reproducibles y las hallazgos neurofisiológicos intraoperatorios se correlacionan con la clínica postoperatoria.

**Alteraciones Campimétricas y Potenciales Evocados Visuales Intraoperatorios: Serie de Casos**

Raidili Cristina Mateo Montero^1^, Estela Lladó-Carbó^1^, JOAN Conill Ramón^2^, Romà Solà Jürschik^2^, Jon Olabe Goxencia^3^, Javier Olabe Jáuregui^3^

^1^NEURTOC y HM Cataluyan, Barcelona, España

^2^NEUROTOC, Barcelona, España

^3^Quirón Hospitales, Palma de Mallorca, Palma, España

**Introducción y objetivos**: Los potenciales evocados visuales (PEV) como técnica de MIO de la función visual, se utilizaron por primera vez durante la cirugía intraorbital en 1973 pero han sido criticados por ser poco fiables y reproducibles, no siendo adoptados de forma estándar en la práctica común.Se estima que la sensibilidad y la especificidad de la amplitud de la PEV para detectar cambios es de un 75% y 79% respectivamente.Nuestro objetivo es describir el comportamiento neurofisiológico de los PEV intraoperatoriamente y valorar si los pacientes con déficit campimétricos presentan inversión de fase en el montaje O1-O2. **Material y métodos**: Realizamos un trabajo retrospectivo y descriptivo de los PEV intraoperatorios en una serie de 23 pacientes durante cirugía endoscópica o cirugía abierta bajo anestesia general TIVA. La MIO se realizó con gafas de flash LED de Inomed de forma mono y binocular, fijadas sobre los párpados cerrados con esparadrapo, La estimulación con flash LED de luz blanca suministrada por 3 LEDs con una potencia de 5500 lx cada uno (potencia total 16.500 lx) siendo excitado el PEV con una intensidad de 20 mA, se adquirieron a intervalos regulares. La duración del estímulo fue de 1 ms y la frecuencia de estimulación de 1,1 Hz. El montaje de registro: (OZ-A1, O1-O2, OZ-FZ y FZ-A1). **Resultados**: Los potenciales fueron reproducibles en el 100% de los casos durante toda la cirugía, 8 de los 23 pacientes presentaron hemianopsia previa a la cirugía, de los cuales 7 presentaron inversión de fase. Se registraron 7 pacientes con latencia de la p100 aumentada y 4 asimétricos. En 8 de los casos se registraron cambios de los VEP y en 5 casos se registró mejoría intraquirúrgica coincidiendo con mejoría clínica de los pacientes. **Discusión/Conclusiones**: Los PEV nos permiten monitorizar la función de la vía visual durante la cirugía, siempre que se realice el montaje adecuado y que los registros sean reproducibles, además nos puede aportar información referente al déficit campimétrico pre e intraoperatorio y darnos un valor pronóstico.

**Alarmas MNIO Durante el Abordaje Quirúrgico de Tumores Intramedulares y su Impacto en el Pronóstico Postoperatorio**

Olga Fedirchyk Tymchuk^1^, Lidia Cabañes Martínez^1^, Estrella Barrero Ruiz^1^, Elena Torras de Caralt^1^, Guillermo Martín Palomeque^1^, Luis Ley Urzaiz^1^, Ignacio Regidor^1^

^1^Hospital Universitario Ramón y Cajal, Madrid, España

**Introducción y objetivos**: Los tumores intramedulares son neoplasias poco frecuentes del sistema nervioso, sin embargo su tratamiento quirúrgico supone un reto para el neurocirujano y también para el neurofisiólogo. Los potenciales evocados motores transcraneales (TcMEP) y la onda D son técnicas neurofisiológicas intraopetarorias que se correlacionan con el pronóstico funcional neurológico después de la cirugía. **Material y métodos**: Se describen dos casos clínicos de pacientes con diagnóstico de tumor intramedular cervical en los que se identificaron cambios significativos interpretados como alarmas intraoperatorias en la MIO antes de la apertura dural y después de su cierre, respectivamente. **Resultados**: En el primer caso se trata de una paciente con diagnóstico de un ependimoma C2-T1. Durante la laminotomía se objetivó una disminución significativa de amplitud de TcMEP en el lado derecho y más tarde en el lado izquierdo, ambos en relación con la manipulación de las láminas, que fue extensa. No se obtuvo onda D. La paciente se despertó con paraparesia de predominio izquierdo caudal a la lesión. En el segundo caso, se trata de una paciente con un tumor glial de alto grado cervical C4-C5. La alarma intraoperatoria que condicionó el fin de la resección fue una disminución significativa unilateral de TcMEP caudales a la lesión con preservación de la amplitud de la onda D. Durante la recolocación de las láminas se objetivó una pérdida brusca bilateral de TcMEP caudales a la lesión, con recuperación parcial unilateral derecha al final de la monitorización. La paciente se despertó con déficit sensitivo y paraparesia de predominio izquierdo caudales a la lesión. **Discusión/Conclusiones**: Las alarmas intraoperatorias en la cirugía de tumores intramedurales pueden ocurrir en cualquier momento de la cirugía, pudiendo dificultar la interpretación de los hallazgos durante la fase de resección y, de esta manera, condicionar la determinación del correcto pronóstico funcional postopertatorio.

**Utilidad de la Monitorización Intraoperatoria en la Estimulación Cerebral Profunda del Núcleo Subtalámico en la Enfermedad de Parkinson**

Sebastián Balay D’Agosto^1^, Beatriz Lozano Aragoneses^1^, Paula Carvajal García^1^, Maruvic Boruska Ramírez Arvelo^1^, Gianclaudio Dal Boni Gomez^1^, A.J. Soriano García^1^, Consuelo Valles Antuña^1^

^1^Hospital Universitario central de Asturias, Oviedo, España

**Introducción y objetivos**: La estimulación eléctrica repetitiva de alta frecuencia a nivel del núcleo subtalámico (NST) mediante estimulación cerebral profunda (DBS) es una terapia bien establecida para el tratamiento sintomático de la enfermedad de Parkinson (PD). Para alcanzar el núcleo diana utilizamos estereotaxia, obteniendo unas coordinadas iniciales (neuroimágen), que luego son complementadas mediante el registro multiunitario de la actividad bioléctrica cerebral durante la monitorización intraoperatoria (IOM). La utilidad de la IOM está siendo debatida por algunos autores en la literatura actual. **Objetivo**: determinar la utilidad de la IOM en la DBS del NST bilateral en la PD. **Material y métodos**: Estudio retrospectivo, revisando los registros neurofisiológicos de la implantación de 40 electrodos profundos en NST de 20 pacientes con PD, en 2018 a 2020, en nuestro centro; así como informes clínicos de neurocirugía y neurología. Utilizamos test estadístico pareado no paramétrico (Wilcoxon), y t-student. **Resultados**: Las coordenadas iniciales se vieron modificadas en el 55% de los pacientes, guiado por la IOM. Las medias de las coordenadas iniciales para hemisferio derecho fueron x = 11,16, y = –2,77, z = –6,94; hemisferio izquierdo x = –11,57, y = –3,32, z = –6,86. Las medias de la coordinadas finales para hemisferio derecho fueron x = 11,30, y = –3,17, z = –7,12; hemisferio izquierdo x = –11,52, y = –3,33, z = –6,99. Se observaron diferencias (no significativas) entre coordenadas iniciales y finales, a derecha x = 0,14, y = –0,4, z = –0,18; e izquierda x = 0,05, y = –0,01, z = –0,13. Mediante la estimulación intraoperatoria se obtuvo una mejoría intraoperatoria en el 100% de los pacientes a nivel de rigidez, temblor y/o bradicinesia. Parámetros más utilizados: intensidad = 1 mA, frecuencia = 130 Hz y duración = 60 microseg. Efectos adversos: contracción tónica transitoria de musculatura perioral (5%). Se observó un descenso significativo (*p* < 0,01) de 18,3 puntos en UPDRS III a los 6 meses (47,7% respecto a previo). Complicaciones (7,5%): relacionadas con el procedimiento quirúrgico (sondaje vesical y dehiscencia de herida). **Discusión/Conclusiones**: La IOM de la DBS ayuda en el mapeo del NST e influye en la localización de los electrodos, esto provoca un aumento de la eficacia terapéutica de la DBS, sin causar un aumento significativo de complicaciones.

**Nictalopía por Hipovitaminosis A Secundaria a Síndrome de Intestino Corto: Cuando el ERG es la Clave Diagnóstica**

Moisés León Ruiz^1^, Andrea Gómez Moroney^1^, Marta Naranjo Castresana^1^, Carlos Castañeda Cabrero^1^

^1^Sección de Neurofisiología Clínica, Servicio de Neurología, Hospital Universitario La Paz, Madrid, España

**Introducción y objetivos**: La hipovitaminosis A (HA) es una causa identificable y tratable de nictalopía. Su diagnóstico se basa en la anamnesis, la exploración neurooftalmológica y los niveles séricos de retinol <0.3 µg/mL y/o retinol/proteína trasportadora de retinol <0.81. Presentamos un caso excepcional de nictalopía secundaria a HA, por un síndrome de intestino corto (SIC), siendo la clave diagnóstica inicial el electrorretinograma (ERG). **Material y métodos**: Mujer de 67 años, con cáncer gastrointestinal, resecciones gástrica (1994) y de intestino delgado (1999 y 2010), habiendo recibido quimiorradioterapia adyuvante, con esteatorrea, tomando vitaminas A+E+B hasta 2021, suspendidas motu proprio, iniciando 2 meses después nictalopía. Sin alteraciones en BMC, presión intraocular, fondo de ojo y RM craneal; con pérdida de células ganglionares en OI en OCT, solicitándose PEV y ERG de campo completo. **Resultados**: PEV: incremento de latencias en ambos ojos. ERG: afectación extensa de la retina en condiciones escotópicas (bastones y células bipolares). Analítica sanguínea: hipovitamonis A (principal), D y E, por SIC post-quirúrgico y –radioterapia. Tras reposición multivitamínica (parenteral y oral), se objetivó resolución de la nictalopía y el ERG (7 meses después). **Discusión/Conclusiones**: La HA es rara en el mundo desarrollado, pudiendo ocurrir por absorción intestinal reducida tras un SIC. El 11-cis-retinal (cromóforo fotosensible de los fotorreceptores) deriva de la vitamina A, por lo que la HA afecta a la sensibilidad de los fotorreceptores, siendo los bastones más vulnerables, con amplitudes reducidas del ERG en condiciones escotópicas, generalmente normales en fotópicas, observándose un ERG negativo (b/a <1; por alteración en la posfototransducción fotorreceptor-célula bipolar; con un incremento de latencias en los PEV). Ante un paciente con nictalopía adquirida, retraso en los PEV, ERG negativo, SIC y mejoría clínica, analítica y ERG tras reponer vitamina A, hay que sospechar una HA, siendo el ERG fundamental para un diagnóstico precoz.

**Valoración del Tratamiento con TDCS en Pacientes con Síndrome de Dolor del Miembro Fantasma. Serie de Casos**

Laís Alexandra Reinoso Aguirre^1^, Yolanda Pérez Borregoa Pérez Borregoa^2^, Antonio Oliviero^2^, Beatriz García López^1^, Jennifer Paola Cantarero Durón^1^, Fernando Vázquez Sánchez^1^, Joseba Soto Ibáñez^1^

^1^Hospital Universitario de Burgos, Burgos, España

^2^Hospital Nacional de Parapléjicos, Toledo, España

**Introducción y objetivos**: El dolor del miembro fantasma (DMF) puede aparecer tras una amputación por trauma o enfermedad vascular periférica con una incidencia entre 42,2 y 78,8%. La estimulación transcraneal con corriente directa (tDCS) es una técnica que permite aplicar una corriente eléctrica de baja amplitud sobre el cuero cabelludo, indolora y con muy pocos efectos adversos. **Objetivos**: describir 2 casos clínicos y el protocolo utilizado en pacientes con DMF que recibieron tratamiento con tDCS en el Hospital Nacional de Parapléjicos de Toledo (HNP). **Material y métodos**: Se describen 2 pacientes de 73 y 48 años, que experimentaron la amputación de una extremidad, con 16 y 6 años de evolución del dolor, a los que se les inició tDCS, entre enero y marzo 2021, realizando un protocolo de estimulación con dos fases; inducción (10 días) y mantenimiento cada 15 días, con un seguimiento de la valoración del dolor a través de una escala de análisis visual (VAS), que se puntúa de 0–100. Cada sesión tuvo una duración de 20 min y se empleó corriente directa sobre el cuero cabelludo a 2 mA, estimulando en la corteza M1, de forma unilateral y contralateral al dolor (extremidad faltante), con el cátodo sobre corteza frontal ipsilateral. **Resultados**: Se analizó el tiempo total de tratamiento para ambos casos, 3 años–5 meses y 7 meses (abandono de tratamiento). Paciente1; obtuvimos una VAS media inicial de 85, una VAS media en el último mes de 40, reducción significativa del dolor (>20 puntos). Paciente2; registramos una VAS media inicial de 79 y una VAS media del último mes de 65. Analizando cada sesión de forma individual, observamos una disminución entre la VAS antes y después del tratamiento en el 86.5% de las sesiones (paciente1) y en el 91.7% (paciente2). **Discusión/Conclusiones**: La aplicación de tDCS puede llegar a tener un papel relevante en el tratamiento del DMF. Si bien, se plantea la necesidad de realizar más estudios con mayor muestra, destacamos la prolongada continuidad del tratamiento en uno de los pacientes, lo cual nos permite valorar los efectos a largo plazo.

**Estudio del Nervio Femorocutáneo Anterior Como Herramienta Complementaria en el Diagnóstico de la Meralgia Parestésica**

Africa Bueno García^1^, María Martín Carretero^1^, Christian Javier Hernández Aranda^1^, Jose Miguel León Alonso-Cortés^1^, Gonzalo Ferrer Ugidos^1^, Sara Muniesa Lacasa^1^, Margely Abete Rivas^1^

^1^Hospital Clínico Universitario de Valladolid, Valladolid, España

**Introducción y objetivos**: Comparar respuestas de los potenciales evocados somatosensoriales (PESS) entre nervio cutáneo femoral lateral y nervio cutáneo anterior (rama del nervio femoral), evaluando las diferencias en pacientes con hipoestesia en muslo, determinando la utilidad de estos estudios en la diferenciación entre neuropatía femorocutánea y radiculopatía. **Material y métodos**: Se realizaron PESS estimulando en dermatomas correspondientes a nervios cutáneo femoral lateral y nervio cutáneo anterior. Las respuestas fueron registradas en Fz y Cz. El estudio incluyó a 10 pacientes sanos (control), y 12 pacientes con clínica compatible con meralgia parestésica. Las latencias absolutas de los potenciales P31, N49 y P53 fueron medidas y comparadas. Completándose con electromiografía (EMG) territorios L2, L3, L4, L5 y electroneurografía (ENG) de nervio cutáneo femoral lateral, peroneal, tibial posterior, sural y peroneal superficial. **Resultados**: De 12 pacientes con hipoestesia, 4 presentaron resultados normales en los PESS y en el resto del estudio neurofisiológico. En 5, se demostró una afectación exclusiva del nervio cutáneo femoral lateral y el nervio cutáneo anterior no mostró alteraciones. En pacientes con radiculopatía motora lumbar de los niveles estudiados, no se observó retraso en las respuestas de los PESS. La normalidad de señales corticales al estimular la cara medial del muslo y un resultado de PESS anormal del nervio cutáneo femoral lateral orientaron una lesión del nervio periférico. **Discusión/Conclusiones**: La afectación del nervio cutáneo femoral lateral puede presentarse de forma aislada sin comprometer el nervio femorocutáneo anterior en pacientes con meralgia parestésica. En casos de radiculopatía motora lumbar, las respuestas de los nervios femorocutáneos no se ven afectadas. La normalidad de las respuestas del nervio femorocutaneo anterior, junto con PESS anormales del nervio femorocutáneo lateral sugiere una lesión del nervio cutáneo femoral lateral, apoyando la utilidad de los PESS para diferenciar las neuropatía y radiculopatías.

**EMTCr Como Tratamiento en la Enfermedad de Parkinson. Nuestra Experiencia en el Hospital de Manises**

Araceli Isabel Soucase Garcia^1^, Andrea Victoria Arciniegas Villanueva^1^, Emilio González García^1^, Jeimmy Mabel Pinzón Martínez^1^, María José Ortiz Muñoz^1^, Carla Andrea Artacho Pérez^1^, Ángela María Suero Cubilete^1^

^1^Hospital de Manises, Valencia, España

**Introducción y objetivos**: La Enfermedad de Parkinson (EP) es una enfermedad neurodegenerativa con síntomas motores y sistémicos que han recibido gran atención últimamente. Causan una disminución considerable en la calidad de vida. El tratamiento incluye terapia farmacológica, psicológica y rehabilitadora, sin que la respuesta sea buena en todos los pacientes. La Estimulación Magnética Transcraneal repetitiva (EMTr), es un método alternativo que permite neuromodular las redes neuronales implicadas en la EP. Nuestro objetivo es valorar la eficacia del tratamiento de EMTr en pacientes con EP y con ello la mejoría en su calidad de vida. **Material y métodos**: Estudio observacional prospectivo de un solo brazo, de pacientes con EP establecida, que han visto afectada su calidad de vida. Bajo estrictos criterios de inclusión y exclusión, se aplicó protocolo de EMTr HF (10 Hz) sobre el córtex primario motor bilateral, con valoraciones clínicas periódicas y con la Escala Unificada de la Enfermedad de Parkinson (UPDRS) para medir el funcionamiento cognitivo, la conducta y el ánimo; las actividades de la vida cotidiana y la función motora. **Resultados**: Incluimos 12 pacientes, y cumplimentaron la UPDRS pre y post tratamiento. En la subescala I (estado mental, comportamiento y humor), mejoraron o se mantuvieron estables el 75%. En la subescala II (actividades cotidianas), no hubo cambios significativos. En la subescala III (exploración motora), mejoraron el 75%. Por último, en la subescala IV (complicaciones del tratamiento), presentaron menos efectos secundarios el 58,3%. **Discusión/Conclusiones**: La EMTr-HF se usa para mejorar el deterioro motor en la EP. En nuestros pacientes ha sido útil en la disminución de los mecanismos inhibitorios que generan la bradicinesia, la rigidez y la incapacidad funcional del temblor. La mejoría más evidente es en la sintomatología motora y el fenómeno on-off. En resumen, pacientes complejos con EP resistentes a tratamientos habituales, se podrían beneficiar con EMTr, mejorando su sintomatología y su calidad de vida.

**Notalgia Parestésica a Propósito de un Caso**

Raquel López-Carvajal Hijosa^1^, M. Rodríguez Jiménez^1^, M. Picornell Darder^1^

^1^Hospital Universitario de Móstoles, Madrid, España

**Introducción y objetivos**: Introducción: La notalgia parestésica es una neuropatía sensitiva secundaria a una alteración en las terminaciones cutáneas de la rama posterior de los nervios espinales T2-T6. Cursa con mácula hiperpigmentada de bordes irregulares y dolor localizado. Objetivos: Correlacionar los síntomas reportados y los hallazgos de la exploración física con las alteraciones encontradas en los potenciales somatosensoriales. **Material y métodos**: Mujer de 58 años con dolor tipo ardor y quemazón en región escapular izquierda de cuatro meses de evolución, junto con mácula hiperpigmentada asociada a prurito. Se realizan potenciales somatosensoriales por dermatomas de T2 a T8 comparando el lado afecto con el lado sano. La técnica de estimulación se realiza con electrodos de superficie en T2, T4, T6 y T8 en relación a los mapas dermatómicos de Foester y Keegan and Garret, con pulsos de 0,2 sg, frecuecia de 3/3,1 Hzs e intensidad 2,5 X umbral sensitivo. La técnica de registro se realiza con electrodos de superficie en Cz´-Fz, con un filtro 5–2000 Hzs y barrido 100 ms (10 ms/D), 300 promediaciones y comparación interlados. **Resultados**: Tras la realización de los potenciales somatosensoriales por dermatomas de T2 a T8 bilaterales se obtienen respuestas simétricas en T2, T4 y T8 (latencias y amplitudes similares en ambos lados) excepto en el dermatoma T6, en el cual no se obtiene respuesta en el lado izquierdo, pero sí se obtiene potencial en el lado derecho. **Discusión/Conclusiones**: Importancia del diagnóstico precoz de la notalgia parestésica, ya que se trata de una entidad poco frecuente e infradiagnosticada que se beneficiaría de un tratamiento precoz, desde tópicos como la capsaicina o sistémicos como la gabapentina.

**Electrorretinograma de Campo Completo Portátil en Diabéticos Tipo 1 de Larga Evolución sin Retinopatía Diabética**

Marta Arias Álvarez^1^, Inés Vicente Garza^1^, María Sopeña Pinilla^2^, Cristina Tomás Grasa^1^, Elvira Orduna Hospital^3^, Diego Rodríguez Mena^1^, Isabel Pinilla^1^

^1^Hospital Clínico Universitario Lozano Blesa, Zaragoza, España

^2^Hospital Universitario Miguel Servet, Zaragoza, España

^3^Universidad de Zaragoza, Zaragoza, España

**Introducción y objetivos**: La retinopatía diabética (RD), una complicación ocular de la Diabetes Mellitus (DM), es la principal causa de ceguera irreversible en edad laboral, siendo especialmente severa en diabéticos tipo 1 (DM1), lo que representa un problema de salud mundial. Se ha reconocido que la RD es una enfermedad neurovascular, con neurodegeneración inicial en las células de la retina interna y gliales, lo que genera cambios estructurales y funcionales. El objetivo fue detectar precozmente alteraciones funcionales en DM1 de larga evolución sin RD, utilizando electrorretinograma (ERG) de campo completo portátil, para un manejo efectivo y preservación de la visión. **Material y métodos**: Estudio prospectivo que incluyó 23 DM1 con más de 20 años de evolución sin RD y un grupo control de edades similares. Se realizó historia clínica, exploración oftalmológica y estudio neurofisiológico utilizando el sistema portátil RETeval, sin midriasis, aplicando el protocolo de evaluación de la RD. **Resultados**: La edad media fue de 48,00 ± 9,77 años en DM1 y 51,69 ± 4,75 años en el grupo control. La duración media de la DM1 fue de 28,69 ± 6,6 años. En DM1 se observó una disminución en la amplitud en la respuesta de bastones, un aumento en el IT de la onda a y una reducción en la amplitud de la onda b en la respuesta mixta a 280 Td.s, un incremento en el IT y una disminución en la amplitud de OP2 y OP3, así como un aumento en el IT y una diminución en la amplitud de la respuesta a 16 Td.s a 28.3 Hz. Los resultados fueron similares a los obtenidos con el ERG convencional. Todos estos hallazgos fueron estadísticamente significativos (*p* < 0,05). **Discusión/Conclusiones**: Los pacientes con DM1 de larga evolución y sin RD muestran principalmente alteraciones en el ERG bajo condiciones escotópicas, especialmente en los potenciales oscilatorios, al usar el sistema de ERG portátil. Esto destaca la relevancia de este sistema para la detección temprana y el seguimiento de la RD.

**Utilidad de los Potenciales Evocados Somatosensoriales Para el Apoyo Diagnóstico. A Propósito de un Caso**

Alba González^1^

^1^Hospital Fundación Jiménez Díaz, Madrid, España

**Introducción y objetivos**: Los potenciales evocados somatosensoriales son una técnica neurofisiológica utilizada como herramienta para evaluar la integridad y el funcionamiento de la vía sensorial, desde los nervios periféricos hasta la corteza cerebral. Tras obtener respuestas, estas son analizadas para saber si hay alguna alteración en en estas vías; su utilidad también puede utilizarse para determinar severidad y localizar la lesión. Para poder dar una conclusión de apoyo al diagnostico si sospechamos de lesiones, es importante conocer de antemano el funcionamiento fisiológico de estas vías y de la interpretación de las señales. **Material y métodos**: Comprender la importancia del conocimiento de la vía somatosorial para su evaluación clínica, además de identificar las respuestas obtenidas tras la realización de la prueba. Exponer los ejemplos más comunes que nos podemos encontrar en la consulta de neurofisiología y discutir un caso clínico al que se realizan potenciales evocados somatosensoriales por alteraciones sensitivas y motoras de años de evolución. Realizamos potenciales evocados somatosensoriales con estimulaciones de ambos nervios medianos y tibiales posteriores y registro FZ-CZ/FZ-C3’/FZ-C4’; además se realizan conducciones de nervios nervios peroneal profundo y superficial, tibial posterior, sural y nervio mediano y cubital de lado derecho. **Resultados**: Se observan unas respuestas alteradas, por lo que se solicita prueba de imagen (RNM) para complementar el diagnóstico. **Discusión/Conclusiones**: El estudio de PESS y la prueba de imagen mostraron que la clínica de la paciente se debía a un compromiso medular, en este caso un meningioma a nivel dorsal; podemos concluir que que los PESS proporcionan información objetiva sobre posibles alteraciones en la conducción nervisa y pueden ser indicativos de compresión medular. Es una herramienta complementaria contribuyendo a un diagnóstico más preciso y una mejor planificación del tratamiento.

**Hallazgos Electrorretinográficos del Síndrome de Usher: A Propósito de dos Casos**

Reetika Mukesh Baharani Baharani^1^, Patricia Vázquez Alarcón^1^, Sara Giménez Roca^1^, Víctor Hugo Rubio Suárez^1^, Pilar Rosario Martínez Martínez^1^, Julián Vázquez Lorenzo^1^, María de la Paz Moreno Arjona^2^

^1^Hospital Clínico Universitario Virgen de la Arrixaca de Murcia, Murcia, España

^2^Hospital Universitario Reina Sofía de Murcia, Murcia, España

**Introducción y objetivos**: El síndrome de Usher (USH) es una enfermedad genética autosómica recesiva con una prevalencia de 1–9/100.000 habitantes. Se caracteriza por la presencia de pérdida auditiva neurosensorial, retinosis pigmentaria (RP) y disfunción vestibular variable. Este síndrome constituye la primera causa de sordo-ceguera hereditaria. **Material y métodos**: Se exponen los hallazgos electrorretinográficos de dos pacientes con diagnóstico genético de USH estudiados en el Servicio de Neurofisiología Clínica del Hospital Clínico Universitario Virgen de la Arrixaca de Murcia. **Resultados**: Presentamos dos casos de varones de 8 y 23 años afectos por USH, ambos portan implantes cocleares bilaterales desde el primer año de vida y fueron diagnosticados genéticamente objetivándose variantes patogénicas de los genes *PCDH15* y *MYOA7*, respectivamente. Clínicamente presentan una disminución de la agudeza visual con mayor afectación de la visión nocturna, asociando endotropia acomodativa e hipermetropía en el primer paciente; y, miopía y cataratas bilaterales en el segundo. El estudio electrofisiológico de la visión en el varón de 8 años incluyó potenciales evocados visuales (PEV) y electrorretinograma (ERG) con electrodos de superficie infraorbitarios, mientras que en el adulto se realizaron PEV y ERG con electrodos corneales tipo DTL. En ambos casos se observaron hallazgos compatibles con retinopatía difusa severa bilateral, con mayor afectación de los bastones frente a los conos. **Discusión/Conclusiones**: Se estima que el 16% de las RP son sindroḿicas siendo las maś frecuentes las asociadas al USH. El fenotipo clínico-electrorretinográfico de nuestros pacientes coincide con el descrito en la literatura. El ERG puede ser de ayuda en el diagnóstico del USH y serańecesario realizarlo siempre en el contexto de una sordera profunda congeńita. Cabe destacar la importancia de realizar un diagnóstico temprano de esta patología, para poder así ofrecer consejo genético de forma precoz a la familia.

**Estudio de la vía Visual Mediante Uso Combinado de PEV, ERGp y PhNR**

María Fernández-Fígares Montes^1^, Esther Miralles Martín^2^, Guillermo Milano Sebastián^1^, María José Postigo Pozo^1^, José Antonio Sáez Moreno^2^, Victoria E. Fernández Sánchez^1^

^1^Hospital Regional Universitario de Málaga, Málaga, España

^2^Hospital Universitario San Cecilio, Granada, España

**Introducción y objetivos**: La exploración neurofisiológica de la vía visual permite objetivar la función de células retinianas y la transmisión por nervio óptico y vías visuales hasta áreas corticales occipitales. Clásicamente se han usado los Potenciales Evocados Visuales (PEV) pero la interpretación aislada puede inducir a error. Actualmente se propone estudiar la función retiniana y CCGG mediante Electroretinograma patrón (ERGp) y Respuesta fotópica negativa (PhNR). **Material y métodos**: Se ha revisado la bibliografía en sospecha de neuropatía óptica en articulos publicados en los últimos 20 años en los que se haya realizado PEV y al menos ERGp o PhNR. Los hallazgos se han correlacionado con la etiología subyacente y el tiempo de evolución. **Resultados**: Las retinopatías se asocian a alteraciones en los PEVp, la realización del ERGp confirmará la existencia de afectación retiniana diferenciándolo de una neuropatía óptica. En maculopatias existe una caída de amplitud de P50 y N95 del ERGp, la ratio N95/P50 no está reducida. La reducción selectiva de N95 se ha asociado a afectación primaria de CCGG (glaucoma o neuropatía óptica hereditaria). En el glaucoma, el ERGp es la prueba más precoz, la amplitud de la PhNR estará disminuída en relación con la severidad del glaucoma, siendo especialmente útil cuando la OCT no haya sido valorable. En caso de bloqueo de la conducción, de forma aguda se afecta la latencia de P100 con ERGp y PhNR normales, pero tras 2 o 3 semanas, la afectación de N95 o PhNR implica afectación axonal secundaria y precede a los cambios estructurales en OCT. Esto puede ocurrir también neuropatías compresivas o traumáticas. En casos de compresión quiasmática o hipertensión craneal la alteración de PhNR podria ser recuperable y factor pronóstico de la recuperación visual. **Discusión/Conclusiones**: Los PEV no deben realizarse de forma aislada y deben realizarse junto a ERGp y/o PhNR. Dada la falta de disponibilidad de Ganzfeld en la mayoría de laboratorios los nuevos disposivos de mano para estudio de PhNR podrían ser útiles.

**¿Es útil el Electrorretinograma? Estudio Descriptivo de Una Serie de Casos de 2 Años**

Ángela Sánchez-Luis^1^, Enzo von Quednow Mannucci^2^, David Gil Ruiz^3^, Raúl Armas Zurita^3^, Laura Menéndez Rúa^3^, Shijia Li Chen^1^, Fadi Hallal Peche^3^

^1^Servicio de Neurofisiología Clínica, Hospital Universitario Miguel Servet, Zaragoza, España

^2^Servicio de Neurofisiología Clínica, Complejo Hospitalario Universitario de Albacete, Albacete, España

^3^Servicio de Neurofisiología Clínica, Hospital Central de la Defensa Gómez Ulla, Madrid, España

**Introducción y objetivos**: El electrorretinograma de campo completo o Ganzfeld (ERG-G) consiste en el registro de la respuesta eléctrica de las diferentes capas de la retina inducida por estímulos luminosos difusos. Esta respuesta consta de onda a (negativa), que se origina en los fotorreceptores, onda b (positiva) con origen en las células gliales de Müller y las células bipolares, y ondas e, o potenciales oscilatorios, formadas en las capas medias de la retina (células amacrinas). **Material y métodos**: Se ha realizado un estudio descriptivo y retrospectivo de una serie de casos consecutiva de 2 años de duración (05/2022 a 05/2024) de todos los ERG-G realizados en el Hospital Central de la Defensa (HCDGU). La edad media fue de 51,2 ± 22,3 años (rango: 12–89). El ERG-G se realizó según el protocolo de la ISCEV. Se realizó una revisión de la literatura. **Resultados**: Un total de 120 ojos fueron estudiados mediante ERG-G, de los cuales 31 (25,8%) fueron patológicos. De ellos, el 6,5% mostraron datos de afectación exclusiva de bastones, el 12,9% afectación exclusiva de conos, el 29,0% afectación exclusiva de las capas internas de la retina, y el 29,0% afectación de todos ellos. El porcentaje de ERG-G patológicos de nuestra serie (18; 30%) destaca frente a otras series publicadas (2,5–6,6%). Nuestra serie registró 5 ojos con ERG electronegativo o decapitado (tomando como cociente b/a <1,0 o ausencia de ondas a y b). El cociente b/a se mantuvo con tendencia lineal estable con relación a la edad, mientras que las amplitudes de las ondas a y b mostraron una tendencia lineal descendente. El 100% de nuestros pacientes fueron derivados desde Oftalmología y la sospecha diagnóstica más frecuente fue mala visión inespecífica (63,3%). El EOG se realizó tras el ERG-G en 18 ocasiones, siendo en 3 patológico. **Discusión/Conclusiones**: El ERG-G es fundamental para el diagnóstico de retinopatías. Su realización conjunta con el EOG, PEV y OCT mejora su diagnóstico. Nuestra serie muestra un 30% de patológicos, siendo superior a la mayoría de las series publicadas.

**Electrorretinograma y Tomografía de Coherencia Óptica ¿Coinciden?**

Fadi Hallal-Peche^1^, Enzo Von Quednow^2^, Ángela Sánchez-Luis Jiménez^3^, David Gil-Ruiz^1^, Mohamed Sliman-Mohamed^4^, Cielo González-Mendoza^1^, Cristina Moreno-Jiménez^1^

^1^Servicio de Neurofisiología Clínica, Hospital Central de la Defensa Gómez Ulla, Madrid, España

^2^Servicio de Neurofisiología Clínica, Complejo Hospitalario Universitario de Albacete, Albacete, España

^3^Servicio de Neurofisiología Clínica, Hospital Universitario Miguel Servet, Zaragoza, España

^4^Servicio de Oftalmología, Hospital Central de la Defensa Gómez Ulla, Madrid, España

**Introducción y objetivos**: La electrorretinografía de campo completo o Ganzfeld (ERG-G) y la tomografía de coherencia óptica (OCT) son dos importantes herramientas diagnósticas utilizadas para evaluar la función y estructura retiniana, respectivamente. Sin embargo, es común encontrar diferencias en los resultados de ambas. **Material y métodos**: Se ha realizado un estudio descriptivo y retrospectivo de una serie de casos consecutiva de 2 años de duración (05/2022 a 05/2024) de todos los ERG-G realizados en el Hospital Central de la Defensa (HCDGU) y que se les realizó OCT. La edad media fue de 51,2 ± 22,3 años (rango: 12–89). El ERG-G se realizó según el protocolo de la ISCEV. Se realizó una revisión de la literatura. **Resultados**: Del total de 120 ojos a los que se les realizó ERG-G y OCT wide, ambas pruebas coincidieron en un resultado normal en el 46,7%, un resultado patológico en el 13,3% y difirieron en el 40,0%. De los 84 ojos a los que se les realizó ERG-G y OCT macular, ambas pruebas coincidieron en un resultado normal en el 54,8%, en un resultado patológico en el 11,9% y difirieron en el 33,3%. La sospecha diagnóstica más frecuente fue mala visión inespecífica (63,3%), seguido de miodesopsias y retinits pigmentaria (6,7% cada una). La ERG-G es útil para detectar disfunciones en la actividad eléctrica de la retina, como en enfermedades hereditarias de la retina, distrofias de conos y bastones, y otras patologías funcionales. La OCT es útil para detectar cambios estructurales en la retina, como en la degeneración macular relacionada con la edad, el edema macular diabético, agujeros maculares, y otras condiciones estructurales. **Discusión/Conclusiones**: El ERG-G y la OCT son dos pruebas fundamentales en el diagnóstico de retinopatías. Los resultados de ambas pruebas no siempre coinciden debido a sus enfoques diferentes. Una retinopatía difusa leve podría pasar desapercibida en la OCT y una retinopatía focal leve podría no ser significativa en la ERG-G. Ambas pruebas son complementarias y juntas aumentan la sensibilidad diagnóstica en retinopatías.

**PEAT Para la Detección Temprana de Hipoacusias en Recién Nacidos en la Comunidad Valenciana, Nuestra Experiencia**

Andrea Victoria Arciniegas Villanueva^1^, Araceli Soucase Garcia^1^, Emilio González García^1^, Maria José Ortiz Muñoz^1^, Jeimmy Pinzón Martínez^1^

^1^Hospital de Manises, Valencia, España

**Introducción y objetivos**: La hipoacusia neonatal es una condición caracterizada por la pérdida parcial o total de la capacidad auditiva presente al nacer o que se desarrolla tempranamente. La identificación temprana es crucial, ya que se afecta el desarrollo del lenguaje, la comunicación y cognición. En España, la incidencia se estima en 1 a 2 casos por cada 1,000 recién nacidos. Las estadísticas recientes indican que, gracias a los programas de cribado y a las intervenciones tempranas, el pronóstico para los niños con hipoacusia ha mejorado notablemente. Presentamos nuestra experiencia en el Hospital de Manises mediante el cribado por PEAT. **Material y métodos**: Presentamos de forma retrospectiva y consecutiva los pacientes de Junio del 2019 a Mayo del 2024 a los que se les ha realizados los PEAT en las tres primeras semanas de vida dentro del cribado de recién nacido de alto riesgo para hipoacusia neonatal. Se revisaron las historias clínicas y las pruebas de cada uno de los pacientes para valorar el número de pruebas alteradas y número de pacientes derivados a otros centros para tratamiento precoz. **Resultados**: Se valoraron 96 niños de los cuales un 79% tuvieron PEAT normales y el 20% patológicos; de los patológicos, un 63% fueron diagnosticados de hipoacusia de trasmisión y un 36% de hipoacusia neurosensorial. En total 19 niños se han beneficiado de derivación precoz para tratamiento de los cuales 7 han sido derivados para implante coclear. Sensibilidad de la prueba 100%, Especificidad 98%, VPP 95% VPN 100%. **Discusión/Conclusiones**: La puesta en marcha de un protocolo en común en la Comunidad Valenciana, ha facilitado la derivación y el tratamiento temprano de las hipoacusias del recién nacido. En nuestro hospital, la facilidad para valorar los niños antes de las tres primeras semanas mediante PEAT, demuestran que la prueba tiene alta sensibilidad y especificidad para el diagnóstico preciso de hipoacusias. Por tanto, el programa ha demostrado ser eficaz, mejorando significativamente el pronóstico de los niños afectados.

**Encrucijada médico-legal en la consulta de sueño, análisis ante casos de riesgo para la salud colectiva**.

Octavio Jiménez Vega^1^, Daniel Ojeda Boudeling^2^, Giada Buzzacchera^3^, Inmaculada Rodríguez Ulecia^1^, Génesis Daniela Arteaga Requena^1^, Amanda Labrador Rodríguez^1^, Ayoze Nauzet González Hernández^1^

^1^Hospital Universitario de Gran Canaria Doctor Negrín, Las Palmas de Gran Canaria, España

^2^Autónomo

^3^Consejería de Sanidad, Servicio Canario de Salud, Madrid, España

**Introducción y objetivos**: Los Trastornos del Sueño representan una potencial causa de siniestralidad derivada de la excesiva somnolencia secundaria a las distintas patologías del sueño, siendo la AOS la patología más prevalente. Los médicos de la Unidad de sueño son testigos de situaciones críticas que trascienden al individuo, afectando a la colectividad, especialmente en profesiones de alto riesgo (pilotos, conductores o manipuladores de maquinaria pesada). Este escenario plantea un debate complejo en el ámbito médico-legal y ético, donde se debe equilibrar el respeto al secreto profesional con la responsabilidad de proteger la seguridad pública. Aunque existen disposiciones legales que establecen excepciones al deber de confidencialidad, el médico debe considerar el impacto de revelar información sobre la salud del paciente. Excepciones al secreto profesional existen para ciertos profesionales, pero no específicamente para médicos, generando un área de incertidumbre para el clínico. Este proceso no solo implica la evaluación clínica, sino también la ponderación de los principios éticos y legales que rigen la práctica médica. **Material y métodos**: Se expondrán dos casos de pacientes con AOS e IAH patológicos, un conductor de transporte público y un piloto, que experimentan Excesiva Somnolencia Diurna secundaria y tienen baja adherencia a los tratamientos, indicaciones y advertencias. **Resultados**: Se ofrecerá el análisis ponderado por las leyes y el código deontológico de un Médico de la Unidad de Sueño, un Inspector Médico de la Consejería de Sanidad y un Abogado especializado en Derecho Sanitario. Cada uno expondrá su perspectiva sobre el caso, destacando sus aplicaciones potenciales en la práctica clínica. **Discusión/Conclusiones**: En esta encrucijada, el médico debe siempre informar al paciente sobre la necesidad de tratamiento, precauciones y consecuencias de su enfermedad, especialmente casos que representen un riesgo para la comunidad. No existen procedimientos unívocos, el conocimiento de las leyes que rigen la profesión médica son imprescindibles.

**Screening de Trastornos del Sueño en la Unidad de Demencias: un Posible rol del Neurofisiólogo en Primeras Consultas**

Irene Carratalá Nebot^1^, Octavio Jiménez Vega^1^, Alfonso Antonio Naranjo Montero^2^, Fernando Haroldo Cabrera Naranjo^1^, Inmaculada Rodríguez Ulecia^1^, David Cañizo García^1^, Ayoze Nauzet González Hernández^1^

^1^Hospital Universitario de Gran Canaria Doctor Negrín, Las Palmas de Gran Canaria, España

^2^Universidad de Las Palmas de Gran Canaria, Las Palmas de Gran Canaria, España

**Introducción y objetivos**: Los trastornos del sueño pueden exacerbar, anticipar y, en ocasiones, simular síntomas de demencia. La mala calidad del sueño se asocia, a su vez, con un mayor riesgo de deterioro cognitivo y progresión de enfermedades neurodegenerativas. Mejorar la precisión del diagnóstico y la calidad del tratamiento tiene un impacto positivo en la salud global del paciente y sus caregivers, por lo que abordar estos problemas de sueño de manera temprana es crucial en el manejo integral de las demencias. Aspecto que normalmente se infravalora en la gestión del neurólogo (complejidad, carga asistencial, tiempos de consulta). Una Unidad Especializada de Sueño (UES), puede ejercer un rol importante en el screening y su manejo en fases iniciales, en un protocolo que promueve la medicina de oportunidad, el trabajo en equipo y el crecimiento de la Neurofisiología en la actividad asistencial. **Material y métodos**: Se realizó un estudio prospectivo y transversal incluyendo 105 pacientes que acudieron a primeras consultas en la Unidad de Demencias del HUGNDN entre noviembre de 2023 y febrero de 2024. Administrando el Índice de Pittsburgh, las escalas de Somnolencia de Epworth y Síndrome de Piernas Inquietas (SPI), junto a otras características clínicas. Los casos con potencial manejo para la Unidad de Sueño (y sin indicación de seguimiento neurológico), fueron derivados a la UES. **Resultados**: La prevalencia de Trastornos del Sueño fue del 32,4% (TCSR, Parasomnias NREM, ESD…), un 39% presentaba datos de Hipersomnia, mientras que el 17,1% presentan síntomas compatibles con el SPI. Además, un 86,7% de los pacientes presentaban polimedicación. **Discusión/Conclusiones**: Se encontró una mayor prevalencia de Trastornos de Sueño en aquellos pacientes que sólo presentaban quejas cognitivas leves, ello permitió realizar su derivación a la UES. Los pacientes con polimedicación entraron en un protocolo de desprescripción en el que participaron Neurofisiólogos. La detección de los Trastornos del Sueño en esta breve experiencia tuvo un impacto positivo en la asistencia

**Relación Inversa Entre la Latencia del Primer Evento Respiratorio y la Gravedad de la Apnea Obstructiva del Sueño**

Juan Manuel Escobar-Montalvo^1^, Andrea Gómez Moroney^2^, María José Aguilar-Amat Prior^3^, Marta Naranjo Castresana^3^, Milagros Merino Andreu^3^

^1^Servicio de Neurología y Neurofisiología Clínica, Hospital Universitario del Henares, Madrid, España

^2^Servicio de Neurología - Hospital Universitario La Paz, Madrid, España

^3^Unidad de Trastornos Neurológicos de Sueño, Servicio de Neurología - Hospital Universitario La Paz, Madrid, España

**Introducción y objetivos**: La apnea obstructiva del sueño (AOS) se relaciona con somnolencia diurna, fragmentación del sueño y deterioro de la calidad de vida y la salud. Se sospecha que los eventos respiratorios precoces durante el sueño podrían asociarse con mayor severidad de AOS y peor estabilidad del sueño. El objetivo fue analizar la asociación entre la latencia del primer evento respiratorio durante el sueño (LPERS) con la severidad del AOS según el índice de apneas-hipopneas (IAH), el índice de arousals (IA) y el número de despertares (ND), a partir de una muestra polisomnográfica de pacientes con sospecha de AOS. **Material y métodos**: Estudio observacional retrospectivo. La LPRES se definió como el tiempo desde el inicio del sueño hasta la aparición de la primera apnea o hipopnea. La asociación de la LPERS con el IAH, IA, ND, se estudió con la correlación de Spearman y modelos de regresión mediana multivariante ajustados por edad, sexo, índice de masa corporal y consumo de benzodiacepinas y/o antidepresivos. La relación entre la gravedad del AOS según el IAH se estudió con un análisis de supervivencia (curvas de Kaplan-Meier, log-rank test y un análisis post-hoc pareado). La significanción estadística fue <0,05 y los análisis se realizaron con *software* estadístico R (RCoreTeam 2023). **Resultados**: Se incluyeron 106 sujetos (mujeres = 55|varones = 51) de una media de 60 años. Se observó una correlación inversa entre la LPRES y el IAH (Spearman’s Rho = –0,64; *p* < 0,001). Asimismo, la reducción de LPRES se asoció con un incremento del IAH (Coef = –0,15, *p* <0,001), IA (Coef = –0,17, *p* < 0,001) y ND (Coef = –0,73, *p* < 0,001). El análisis de supervivencia mostró que la mediana de supervivencia de la LFRES estuvo inversamente asociada con la gravedad del AOS. **Discusión/Conclusiones**: En nuestra muestra observamos una relación inversa entre la LPRES y la gravedad del AOS, el IAH, el IA y el ND. Estos resultados deben confirmarse en estudios prospectivos de mayor número muestral.

**Hemocromatosis Hereditaria y Movimientos Periódicos de las Piernas. A Propósito de un Caso**

Pablo González Uriel^1^, Laura Lillo Triguero^2^, Andrés Feliciano Vilas Iglesias^3^, Verónica Ángel^4^

^1^HM La Esperanza, Santiago de Compostela, España

^2^Hospital Ruber Internacional, Madrid, España

^3^HM La Rosaleda, Santiago de Compostela, España

^4^HM Santiago de Compostela, Santiago de Compostela, España

**Introducción y objetivos**: La hemocromatosis (HC) es un trastorno hereditario del metabolismo del hierro (Fe), en el que se produce depósito de este metal en el hígado, corazón o cerebro. Los movimientos periódicos de las piernas (MPP) son movimientos estereotipados durante el sueño, que de forma mayoritaria aparecen en los miembros inferiores y durante el sueño NO-REM. En un 80% de los casos están asociados a síndrome de piernas inquietas (SPI), el cual se asocia con un trastorno del metabolismo del Fe a nivel sistémico y cerebral. También existen pacientes con MPP sin clínica de SPI. Presentamos el caso de una mujer con HC hereditaria, sin clínica de SPI, y con MPP identificados en la PSG. **Material y métodos**: Mujer de 53 años en tratamiento con escitalopram y sibelium por AP de depresión y vértigo, que consulta por somnolencia diurna. El acompañante refiere que da patadas de noche en la cama. AP: HC hereditaria tratada con sangrías cada dos meses, fumadora. Escala de Somnolencia de Epworth: 12. Se solicitaron analítica, RM cerebral y PSG. **Resultados**: Analítica: Fe 170 µg/dL, ferritina 298,80 ng/mL, índice de saturación de transferrina 55,56%. RM cerebral de 1,5 T: sin anomalías significativas. PSG nocturno: IAH de 8.1; IAH en REM de 25,9. Hipopneas y apneas obstructivas de corta duración y con escasa repercusión en la SaO_2_. Ronquido frecuente. Los MPP consisten en la flexoextensión de tobillo o rodilla de forma persistente a lo largo de toda la noche, con índice de 68,2/h. **Discusión/Conclusiones**: Tanto el SPI como los MPP suelen ser debidos a trastornos del metabolismo del Fe, siendo ambos favorecidos por la ferropenia sistémica. Existen pocos casos descritos donde exista sobrecarga sistémica de Fe secundaria a HC y MPP con un índice tan grave. Aunque la RM no mostró alteraciones en el contenido cerebral de Fe, se han comunicado casos de HC con SPI tanto con ferropenia como con sobrecarga cerebral de Fe, lo que sugiere que el papel del Fe en el metabolismo cerebral en estas patologías es complejo y no del todo conocido.

**Prevalencia de los Trastornos Respiratorios del Sueño en Niños con Malformación de Chiari Tipos 1 y 1.5**

Alex Ferré Masó^1^, Maria Antonia Poca Pastor^1^, Maria Jose Jurado Luque^1^, Berta Llanas Vidal^1^, Odile Romero Santotomas^1^, Diego Lopez Bermeo^1^, Juan Sahuquillo Barris^1^

^1^Hospital Universitario Vall d´Hebron, Barcelona, España

**Introducción y objetivos**: La malformación de Chiari tipo 1 (CM-1) se han asociado a trastornos respiratorios relacionados con el sueño (TRS), pero se desconoce su prevalencia real en poblaciones pediátricas. **Material y métodos**: El objetivo de este estudio fue evaluar la prevalencia de TRS en una amplia serie de pacientes pediátricos con CM-1 y CM-1,5 de 18 años o menos seleccionados para reconstrucción de la fosa posterior. La presente cohorte se seleccionó a partir de un registro prospectivo de adultos y niños con CM (PROSAC) desde el 1 de diciembre de 2007 hasta el 31 de enero de 2024, que fueron remitidos a la Unidad de Neurocirugía Pediátrica del Departamento de Neurocirugía del Hospital Universitario Vall d’Hebron (HUVH). Los criterios de inclusión consistieron en niños de hasta 18 años de edad con CM-1 y CM-1.5 que no tuvieran ninguna cirugía previa relacionada con Chiari. **Resultados**: Se seleccionaron 97 pacientes pediátricos de la base de datos, con una mediana de edad de 10 (0–18) años y un 49,5% de sexo masculino. La mediana del descenso amigdalar fue de 13,0 mm (3,0–32,0) y 78 (81%) pacientes eran sintomáticos. Las anomalías anatómicas más frecuentes fueron siringomielia [43 (44,8%)], odontoides retrocurvadas [20 (20,6%)] e hidrocefalia [13 (13,5%)]. Para diferentes puntos de corte del índice de apnea-hipopnea (IAH) (IAH ≥1, ≥3, ≥5, ≥10), la prevalencia de TRS fue del 60,8%, 15,5%, 27,8% y 8,2%, respectivamente, con predominio del componente central. La hipercapnia sólo estaba presente en un paciente. En 10 pacientes (13,5%) se observó un trastorno del movimiento periódico de las piernas. La presencia de una odontoides retrocurvada y de síntomas se asoció a un mayor riesgo de TRS (*p* = 0,048 y *p* = 0,044, respectivamente) para el punto de corte 5 del IAH. **Discusión/Conclusiones**: Los trastornos respiratorios del sueño en pacientes pediátricos con CM-1 o CM-1.5 tienen una alta prevalencia con un componente central predominante. Puede existir un mayor riesgo en pacientes sintomáticos y con odontoides retrocurva.

**Aumento de la Sensibilidad Diagnóstica de la Pulsioximetría (POX) Mediante el Análisis Visual del Patrón de Desaturación**

Hyun Suk Oh-Kim^1^, Raúl Armas-Zurita^1^, Juan Riquelme Méndez^1^, Fadi Hallal-Peche^1^, Cristina Moreno-Jiménez^1^, Cielo González-Mendoza^1^, Mariano Vergara-Aguilera^1^

^1^Hospital Central de la Defensa “Gómez Ulla”, Madrid, España

**Introducción y objetivos**: La apnea obstructiva del sueño (AOS) es un trastorno de elevada prevalencia. Se establece la necesidad de buscar pruebas de screening que agilicen el diagnóstico ante las elevadas listas de espera y costes que presenta la polisomnografía (PSG). El objetivo es valorar la POX como herramienta diagnóstica de la AOS frente a la polisomnografía nocturna. Comprobar si existe asociación entre los parámetros pulsioximétricos (índice de desaturación oximétrica (ODI 4%, ODI 3%), tiempo de saturación parcial de oxígeno por debajo del 90% (TD90%), análisis visual del patrón de desaturación) y el diagnóstico de AOS y sus grados. **Material y métodos**: Se planteó un estudio observacional, descriptivo, transversal y retrospectivo en el que se tomaron datos de las pulsioximetrías y polisomnografías de 57 pacientes con sospecha clínica de AOS. En los casos cuyo ODI dio lugar a un falso negativo, se estableció lectura ciega por neurofisiólogos que realizaron un análisis visual del patrón de desaturación. **Resultados**: El análisis conjunto del ODI 4% y del patrón de desaturación presenta una sensibilidad del 93% y un valor predictivo positivo (VPP) del 97%, mientras que el del ODI 4% presenta una sensibilidad del 83% y un VPP de 97%. La lectura del ODI 3% presenta una sensibilidad del 95% y un VPP del 98%. Existe asociación (*p* < 0.05) entre los pacientes con TD 90% < 1% y los grados de AOS moderado y severo. **Discusión/Conclusiones**: La valoración por separado del ODI 3% ofreció mayor sensibilidad y VPP que el análisis conjunto del ODI 4% con análisis visual del patrón de desaturación. En caso de lectura del ODI 4%, se recomienda análisis visual del patrón de desaturación, aumentado de este modo un 10% la sensibilidad del test respecto a la lectura aislada del ODI 4%. El TD90% ≥1% se asoció con grados moderado y severo de AOS en la PSG. Por último, destacamos que el análisis visual del patrón de desaturación puede ayudar en la sospecha de otros tipos de enfermedades pulmonares.

**Polisomnografía en Malformación Arnold Chiari tipo-1 que Debuta con Apneas Centrales del Sueño, a Propósito de un Caso**

Boris Alfonso Orozco Jimenez^1^, Ana Isabel Gómez Menéndez^1^, Olga Pérez Gil^1^, María Carmen Lloria Gil^1^, Joseba Soto Ibáñez^1^, Lais Alexandra Reinoso Aguirre^1^, Gricela María Sorto Bueso^1^

^1^Hospital Universitario de Burgos, Burgos, España

**Introducción y objetivos**: La malformación de Chiari tipo I (CM-I) es una patología rara, caracterizada por un desplazamiento anormal de las amígdalas cerebelosas por debajo del nivel del agujero magno. Los pacientes afectados pueden estar asintomáticos o presentar síntomas insidiosos que pueden suponer retraso del diagnóstico. Entre la variedad de síntomas que se produce por la compresión del tronco del encéfalo, se encuentra el SAHS con predominio de apneas centrales durante el sueño (ACS), que se explica fisiopatológicamente por la compresión con las amígdalas cerebelosas de los nervios craneales IX y X. Presentamos el caso de un niño de 7 años, consulta inicialmente por roncopatía y pausas de la respiración, se solicita polisomnografía (PSG) en nuestro centro que reporta SAHS muy grave predominio ACS por CM-1. **Material y métodos**: Se realizó PSG en niño de 7 años, sin antecedentes perinatales, buen desarrollo pondero-estatural y sin alteraciones del lenguaje. **Resultados**: PSG con resultado SAHS muy severo predominio ACS con desaturaciones, se realiza RNM cerebral donde concluyen CM-1, ingresan a UCIP e inician VMNI durante el sueño. Se realiza cirugía craniectomía fosa posterior, posteriormente PSG con resultado similar al anterior, se hace reintervención quirúrgica y al mes PSG control con SAHS moderado residual y notable mejoría de las ACS. **Discusión/Conclusiones**: En la PSG con SAHS y ACS, debe hacerse estudio de extensión buscando causas centrales SNC. En nuestro paciente, las ACS tuvieron mejoría tras cirugía precoz de la CM-l, persistiendo SAHS moderado residual. Debe hacerse seguimiento estrecho e informar a los familiares sobre la posibilidad de no resolución completa de las ACS tras la cirugía, quedando un margen de apneas residuales y algunos casos con necesidad de soporte ventilatorio durante el sueño.

**Síndrome de Párpado Laxo y Cribado de Apnea del Sueño. A Propósito de Una Serie de Casos**

Goran Josic^1^, Cristina Moreno Jiménez^1^, Paula Arribas Pardo^1^, Mariano Aguilera Vergara^1^, Raúl Armas Zurita^1^, Fadi Hallal Peche^1^, Cielo González Mendoza^1^

^1^Hospital General de la Defensa Gómez Ulla, Madrid, España

**Introducción y objetivos**: La prevalencia de apnea del sueño se estima en 14% hombres y 5% en mujeres. Según la literatura, dicha prevalencia puede ascender al 57,1% entre los pacientes con párpado laxo. Comprobar si existe asociación entre el síndrome de párpado laxo y el diagnóstico de apnea del sueño en nuestra Unidad de Sueño. Descripción de los hallazgos polisomnográficos y clínicos de estos pacientes. **Material y métodos**: Revisión retrospectiva de las polisomnografías cuyo motivo de solicitud fuse sospecha de apnea del sueño, por presentar párpado laxo. Asimismo, registro de clínica cardinal de apnea del sueño a través de la anamnesis dirigida de sueño. **Resultados**: Se recopiló una serie de cuatro casos (3 varones y 1 mujer, de edades comprendidas entre 60–90 años). Resultado polisomnográfico positivo para apnea del sueño en el 100%, de grado severo en la mitad de ellos, y carácter obstructivo en el 75%. La eficiencia de sueño se encontraba reducida, con un valor medio del 62%, y una latencia de sueño media de 34,5 minutos. Arquitectura de sueño fragmentada, no se objetivó sueño REM en la mitad de los casos, con un índice de número de cambios de fase por hora de sueño de 35. La saturación de oxígeno se mantuvo por debajo del 90% una media del 24,4% del tiempo total de sueño. El 50% refería sueño no reparador. Roncopatía, el 50%. La hipersomnolencia diurna no fue llamativa en ninguno de los casos. Todos presentaron factores de riesgo cardiovascular. **Discusión/Conclusiones**: Los resultados en nuestra serie de casos corroboraron la asociación descrita en la literatura entre párpado laxo y apnea del sueño. Resaltar la trascendencia de no ceñirse únicamente al perfil clásico de apnea del sueño para su despistaje. Destacar el papel del enfoque multidisciplinar de estos pacientes en colaboración con Oftalmología, valorando el cribado de apnea del sueño, con el fin de reducir las potenciales complicaciones derivadas de este trastorno de sueño.

**Evaluación Polisomnográfica en un Caso de Síndrome del Cromosoma 20 en Anillo**

Alba González García^1^, Edward Susanibar Mesías^1^, Diana Estephania Blanco-Gómez^1^, Laura Vilella Bertran^1^, Ion Álvarez Guerrico^1^

^1^Hospital del Mar, Barcelona, España

**Introducción y objetivos**: El síndrome del cromosoma 20 en anillo (SC20A) es una condición genética rara en la que se produce una fusión intracromosómica circular. Clínicamente se manifiesta con un perfil epiléptico característico con crisis discognitivas que pueden evolucionar a crisis nocturnas hipercinéticas o sutiles, así como estatus epiléptico no convulsivo. Desde el comienzo de crisis se puede observar además déficit cognitivo así como presencia de alteraciones senso-perceptivas. La caracterización de la polisomnografía (PSG) podría orientar el diagnóstico y la evolución de los pacientes con SC20A. **Material y métodos**: Presentamos a una paciente de 19 años diagnosticada de SC20A a los 3, que debutó con crisis discognitivas y frecuentes episodios de estatus no convulsivo, así como cambios a nivel conductual. Durante su adolescencia, debido a mal control de síntomas con tratamiento farmacológico, se requirió cirugía resectiva del córtex fronto-orbitario, cíngulo y amígdala izquierda y de implantación de estimulador del nervio vago (VNS). Por sospecha de AOS se realizó vídeo-polisomnografía nocturna. **Resultados**: La monitorización electroencefalograma (EEG) prolongada de 3 días de duración previa a la cirugía mostró una actividad basal con enlentecimiento difuso continuo y actividad epileptiforme interictal multifocal muy recurrente, de predominio fronto-temporal izquierdo. En la PSG se registraron grafoelementos irregulares que dificultaron la identificación de los estadios del sueños. Además, se objetivó actividad paroxística, en transición vigilia-sueño, con menor incidencia en NoREM y ausente en REM, así como complejos de onda-aguda onda-lenta, de inicio fronto-temporal izquierdo, indicativa de actividad epileptiforme interictal y crisis electrográficas. **Discusión/Conclusiones**: La PSG es una herramienta escasamente utilizada en el diagnóstico y el seguimiento de pacientes con SC20A. Sin embargo, la presencia de un patrón de sueño característico podría probar ser útil en el abordaje multidisciplinar de esta enfermedad.

**Desafíos en Polisomnografía: Causas del Registro Deficiente y Perspectivas de Mejora**

Luigi Carlo Unda^1^, María Iglesias Tejedor^1^

^1^Hospital Universitario Rey Juan Carlos, Madrid, España

**Introducción y objetivos**: La polisomnografía es una herramienta de diagnóstico confiable, cuya validez depende del tiempo total de sueño (TTS) del paciente. Nuestro principal objetivo es determinar cuántas polisomnografías (PSG) tienen un TTS menor de 4 horas y 30 minutos, identificar los principales factores que influyen en el TTS y evaluar las diferencias en variables entre pacientes con TTS menor y mayor de 4H30. **Material y métodos**: Se realizó un estudio transversal retrospectivo. Los datos de adultos mayores de 18 años se recogieron de formularios generados en el sistema Casiopea del Hospital Universitario Rey Juan Carlos en el año 2023, estos fueron almacenados en una tabla Excel y analizados en el programa SPSS. **Resultados**: Se obtuvo un total de 318 pacientes, 44 tenían un TTS menor a 4H30 y 274 con un TTS mayor a 4H30, de los cuales se seleccionó aleatoriamente una muestra representativa de 65 pacientes. En el grupo con TTS menor de 4H30 había 30 hombres y 14 mujeres, con una edad media de 53,81 años. En el grupo con TTS mayor a 4H30 había 33 hombres y 32 mujeres, con una edad media de 48,32 años. Se registraron las quejas de los pacientes después de la PSG, incluidos “cables, dolor, entorno, ansiedad/inquietud, otros motivos, todo”. En el grupo TTS menos de 4H30, se observó que el 22,7% de los pacientes experimentaron ansiedad/inquietud (10 pacientes vs 0 pacientes, χ^2^ *p* 0,00) y el 13,6% del mismo grupo experimentó dolor durante el estudio (6 pacientes vs 2 pacientes, χ^2^ *p* 0,038). Este grupo percibió que dormir en el laboratorio del sueño era peor que en casa, en comparación con el 23,1% en el grupo con un TTS superior a 4H30 (χ^2^ *p* 0,008). relacionado con “cables, medio ambiente, otros motivos, todo”. **Discusión/Conclusiones**: La ansiedad/inquietud y el dolor afectan a un TTS más bajo del PSG. Muchos pacientes perciben que duermen peor en el laboratorio del sueño que en casa. Esto sugiere enfoques para mejorar el desempeño de una PSG adecuada, optimizar recursos y tener diagnósticos confiables.

**Trastorno de Conducta del Sueño REM y Status Dissociatus. Hallazgos Vídeo-polisomnográficos En una Serie de 3 Casos**

Estefanía García Luna^1^, Diana Peñalver Espinosa^1^, Eugenio Barona Giménez^1^, Marina Villamor Villarino^1^, Andrea Miró Andreu^1^, María de la Paz Moreno Arjona^1^, Roberto López Bernabé^1^

^1^Hospital General Universitario Reina Sofía, Murcia, España

**Introducción y objetivos**: El TCSR es una parasomnia REM que se caracteriza por la pérdida de atonía muscular durante el sueño REM y la aparición de comportamientos motores complejos relacionados con el contenido de los sueños, frecuentemente de naturaleza violenta. El objetivo es establecer los hallazgos en la vídeo-Polisomnografía (V-PSG) en casos de TCSR que evolucionan hacia un Status Dissociatus. **Material y métodos**: Se presentan tres casos clínicos de varones de 71, 72 y 75 años respectivamente, con episodios diarios de vocalizaciones, gritos, golpes y sueños vividos de contenido violento durante el sueño, especialmente en la segunda mitad de la noche con los ojos cerrados. Estos pacientes fueron sometidos a un estudio de V-PSG. **Resultados**: La V-PSG de los dos primeros casos mostró una alternancia de periodos de sueño NREM y REM con ausencia de los grafoelementos y de la atonía característica de la fase REM. Además, se observaron conductas motoras complejas, permitiendo el diagnóstico de TCSR en ambos pacientes. El tercer caso mostró una ausencia completa de las fases del sueño y grafoelementos, siendo diagnosticado de Status Dissociatus en el contexto de Demencia por Cuerpos de Lewy. **Discusión/Conclusiones**: La realización de un estudio V-PSG es necesario para el diagnóstico de estos trastornos de sueño, porque permite identificar la pérdida de atonía y aparición de conductas motoras durante el sueño REM. Además, permite conocer el grado de desestructuración de sueño, permitiendo establecer el diagnóstico evolutivo de un TCSR hacia un SD, o Agrypnia excitata, en estadíos finales. Especialmente en el contexto de patologías neurodegenerativas, la V-PSG proporciona una visión integral de la evolución de estos trastornos, permitiendo una intervención temprana y personalizada, comprendiendo la fisiopatología y así, optimizar el manejo clínico de dichas patologías.

**Taquipnea Neurogénica Durante el Sueño Como Causa de Excesiva Somnolencia Diurna. A Propósito de un Caso**

Liliam Pérez Bauzá^1^, María Fernanda Romero Puertas^1^, Igor Santrich Baranov^1^, Luisa Margarita Cabrera Pimentel^1^

^1^Hospital Universitario Miguel Servet, Zaragoza, España

**Introducción y objetivos**: La taquipnea neurógenica ligada al sueño es una condición poco común caracterizada por un aumento sostenido en la frecuencia respiratoria durante el sueño que revierte en la vigilia. Nuestro objetivo es presentar un caso recientemente visto en la Unidad del Sueño del Hospital Universitario Miguel Servet de Zaragoza. **Material y métodos**: Se realiza una polisomnografía (PSGN) y test de latencias múltiples (TLM) en una paciente de 38 años en estudio por excesiva somnolencia diurna, roncopatía y apneas; con poligrafía previa en agosto del 2023 normal (IAH: 2,5/h). **Resultados**: En el estudio polisomnográfico se observó como dato más llamativo un aumento sostenido de la frecuencia respiratoria durante todas las fases del sueño, siendo ésta más del doble de la observada en vigilia. El IAH (4) fue normal y la saturación media de oxígeno se mantuvo en torno a 91–92%. En el TLMS (4 siestas) se observó una latencia media al inicio del sueño disminuida (3,4 minutos), sin registro de comienzo de Sleep Onset REM (SOREM). **Discusión/Conclusiones**: Los hallazgos sugieren el diagnóstico de taquipnea neurogénica durante el sueño, patología poco frecuente y con escasas referencias bibliográficas; resaltando la importancia de considerar causas más allá de la apnea obstructiva del sueño en pacientes con excesiva somnolencia diurna.

**“Forma Psicótica” de Narcolepsia Tipo 1: Cuando los Sueños se Hacen Reales**

Luis Gabriel Burgos Bustamante^1^, Natalia Bravo Quelle^1^, Aiala Sáez Ansotegui^1^, Pedro José Melgarejo Otárola^1^, Laura Verna Fierro^1^

^1^Hospital General Universitario Gregorio Marañón, Madrid, España

**Introducción y objetivos**: La narcolepsia tipo 1 (NT1) es un trastorno de sueño raro clasificado dentro de las hipersomnias de origen central. Entre sus síntomas, las alucinaciones visuales en la transición sueño-vigilia pueden estar presentes en distinto grado. La bibliografía ha agrupado a aquellos pacientes que manifiestan experiencias alucinatorias intensas o sueños vívidos relacionados con el REM como una “forma psicótica de NT1”, haciendo énfasis en la “atipicidad” de los síntomas. **Material y métodos**: Se presenta el caso de un varón de 19 años con antecedente de Trastorno del Espectro Autista sin discapacidad intelectual o trastorno conductual asociado, quien desde los 13 años ha experimentado somnolencia diurna excesiva y episodios de cataplejía desencadenados por la risa. No tiene síntomas de insomnio. Desde hace un año experimenta alucinaciones hipnagógicas en las que percibe la presencia de personas que lo sujetan o arrastran por la habitación, provocándole temor y gritos; cinco noches por semana. Tras su consulta se decide realizar: polisomnografía nocturna (PSG), test de latencias múltiples (TLM) y estudio de antígenos leucocitarios humanos de clase II (HLA) haplotipo DQB1*06:02. **Resultados**: La PSG reveló una arquitectura de sueño caracterizada por una latencia de sueño corta con una ligera disminución en la eficiencia del sueño (78,5%) y un reparto porcentual de fases adecuado. En el TLM se observó una latencia de sueño media de 4.6 minutos, con la presencia de 4/5 SOREMs. El estudio de HLA fue positivo. Se estableció el diagnóstico de NT1, forma psicótica. Se indicó como único tratamiento Venlafaxina 37.5 mg, remitiendo las alucinaciones y mejorando significativamente los episodios de cataplejía. **Discusión/Conclusiones**: Los síntomas “psicosis-like” de la NT1 representan un reto diagnóstico, especialmente si los pacientes los describen de manera vívida y afecta su calidad de vida. Es esencial que el clínico reconozca este conjunto de síntomas “atípicos” para realizar un diagnóstico y tratamiento adecuados.

**Sueño de Escolares y Adolescentes en Araba: ¿Existen Diferencias Entre Sexos?**

Alexandra Hurubean Kapás^1^, Ainhoa Alvarez Ruiz de Larrinaga^1^, Carla Pia Martínez^1^, Larraitz Ortega-Álvarez^2^, Paula Aréjola de Los Mártires^1^, Maika Tejeda Pérez^3^, Maria Francisca Gacitua Lemus^1^

^1^Unidad de Sueño, Hospital Universitario Araba, 01009 Vitoria-Gasteiz, España

^2^Ingeniería Biomédica, Universidad de Mondragón, 20500 Arrasate, España

^3^Centro de Salud de Dulantzi, 01240 Dulantzi, España

**Introducción y objetivos**: A medida que crecemos la duración del sueño disminuye debido a procesos biológicos, sociales y académicos. Las niñas duermen más horas y menos fragmentado por influencia del estradiol. En la adolescencia existe retraso de fase, mayor fragmentación y menor sueño profundo, influenciado por el eje hipotálamo-hipofisario. Además, se ha documentado mayor estrés en chicas. El objetivo es analizar las diferencias significativas en las horas totales de sueño (TTS) y en los horarios en función del sexo biológico y grupo de edad de escolares (6–13 años) y adolescentes (14–18 años). **Material y métodos**: Estudio observacional transversal de base comunitaria en adolescentes y escolares de Araba mediante cuestionario online a las familias. Análisis estadístico descriptivo mediante medias y desviación estándar (DE) de horas de sueño y horarios. Usamos T-student para comparar medias. **Resultados**: Obtuvimos 553 respuestas válidas; 48% niñas escolares y 49% chicas adolescentes. El TTS en niñas escolares fue de 9,7 DE 0,9 en laborables(L) y 10,2 DE 0,95 en festivos(F) y en niños 9,6 y 10 respectivamente, siendo la diferencia en F estadísticamente significativo (*p* = 0.0366). En cuanto a las adolescentes el TTS fue 7,9 DE 1,2 en L y 9,8 DE 1,3 en F, mientras que en chicos fue de 8,4 DE 0,8 en L y 9,8 DE 1 en F, con una diferencia significativa en L (*p* = 0,0135). Las escolares se levantan en F a las 9 h 18’ DE 0,6 y los niños a las 9 h 6’ DE 0,6, con diferencia significativa (*p* = 0,0086). No se observan diferencias en L ni adolescentes, así como tampoco en el horario de acostarse. **Discusión/Conclusiones**: En nuestra cohorte, se corrobora que las niñas duermen más que los niños, estadísticamente significativo en F a expensas de levantarse más tarde. En la adolescencia, las chicas duermen menos, siendo significativo en L. En los horarios de sueño no se han encontrado diferencias entre sexos. Dados estos resultados, se deben seguir planteando futuras investigaciones sobre cómo diversos factores (sexo, cambios hormonales, expectativas sociales) influyen en el sueño de los jovenes.

**Alteraciones Polisomnográficas en el Síndrome de Sueño Inquieto en Niños. Más Allá de los Movimientos de Piernas**

Raidili Cristina Mateo Montero^1^, Miguel Angel Hernandez Delatorre^2^, Adriana Gomez Dominguez^3^, Alba Diaz Cid^4^, Francisco Gonzalez De La Rosa^2^, Catalina Villa Jurado^2^, Estela Llado-Carbo^1^

^1^NEURTOC y HM Cataluyan, Barcelona, España

^2^HM Nens, Barcelona, España

^3^Hospital Infanta Helena, Huelva, España

^4^Hospital Virgen De Lirio, Alcoi, España

**Introducción y objetivos**: El trastorno del sueño inquieto (RSD) se caracteriza por movimientos durante el sueño en niños, asociando síntomas diurnos debido a un sueño no reparador. Para que un sueño sea de buena calidad, a parte de la cantidad, es necesario la continuidad y profundidad del mismo. Valorar la calidad del sueño de los niños con RSD mediante un estudio polisomnográfico (PSG). **Material y métodos**: Presentamos un estudio retrospectivo de 80 niños con problemas en el mantenimiento de sueño y diagnóstico de RSD idiopático, que cumplían criterios clínicos y PSG. En este estudio se valoraron los siguientes parámetros: arquitectura del sueño, tiempo total de sueño, WASO, eficiencia e índice de arousal. **Resultados**: Los pacientes estudiados tenían edades entre 7 meses y 16 años. El 92% presentó como mínimo una alteración en los parámetros estudiados, siendo lo más característico un sueño fragmentado, con WASO aumentado (95,38%) y baja eficiencia (81,53%). Respecto a la arquitectura del sueño, estuvo alterada en un 81,53%, siendo lo más frecuente un sueño superficial aumentado. Los pacientes con RSD presentan un sueño más superficial y una mayor fragmentación del sueño. Estos hallazgos se suman al conocimiento actual sobre su fisiopatología, sugiriendo que la inestabilidad del sueño podría ser un mecanismo que favorezca la aparición de los episodios motores característicos. **Discusión/Conclusiones**: En conclusión, hemos observado que el RSD muestra una alteración en la eficiencia y eficacia del sueño. Estos resultados junto con las investigaciones recientes apoyan que estas alteraciones pueden afectar la adecuada calidad del sueño. Esto se relaciona con los procesos de cognición, comportamiento y otras áreas de funcionamiento del niño durante el día, así como una disminución de la calidad de vida familiar.

**Asociación Entre Trastornos Afectivos y Patología del Sueño No Tratada**

Miguel Cobo Moreno^1^, Laura Lorente Remón^1^, Antonio Lucas McHugh^1^, Marian Nicolás Vela^1^, Teresa Ortega León^1^, Alberto Galdón Castillo^1^, Carmen Iznaola Muñoz^1^

^1^Hospital Universitario Virgen de las Nieves, Granada, España

**Introducción y objetivos**: La relación entre el sueño y la salud mental ha sido estudiada a lo largo de los años, reconociendo la patología del sueño tanto como un síntoma de enfermedad psiquiátrica, así como un factor de gran impacto en la salud mental. El objetivo de este estudio fue evaluar el estado de salud mental de una muestra de pacientes no diagnosticados y no tratados de trastornos del sueño en su primera consulta a la Unidad de Trastornos del Sueño. **Material y métodos**: Desde enero de 2023 hasta enero de 2024, se recogieron datos de 281 pacientes adultos, clasificados por motivo de consulta en cinco grupos (pudiendo presentar más de un motivo de consulta): insomnio, somnolencia diurna excesiva, ronquidos, trastornos del movimiento y parasomnia. Se utilizó la Escala de Somnolencia de Epworth (ESE) para medir la somnolencia, el Índice de Severidad del Insomnio (ISI) para valorar calidad del sueño y para la valoración de salud mental de los pacientes se evaluó usando las Subescalas de Ansiedad y Depresión Autoevaluadas de Zung (SAAZ, SDAZ). **Resultados**: De la muestra obtenida, 83 pacientes presentaron insomnio, 94 SDE, 126 ronquidos, 26 trastornos del movimiento y 30 parasomnia. En general, el 72,95% de los pacientes exhibieron puntuaciones patológicas en la SAAZ y el 74,02% en la SDAZ, con puntuaciones promedio que indicaban ansiedad y depresión leves. La prueba de correlación de Spearman reveló una asociación leve entre las puntuaciones del SAAZ y la SDAZ y las obtenidas en el ISI de la muestra estudiada, observándose mayor asociación entre los pacientes con ronquidos, trastornos del movimiento y la asociación más alta en pacientes con parasomnia. **Discusión/Conclusiones**: La ansiedad y la depresión están estrechamente vinculadas a los trastornos del sueño, por lo que se recomienda el cribado protocolizado de patología afectiva en pacientes que buscan asesoramiento médico por trastornos del sueño, con el objetivo de realizar un correcto diagnóstico y manejo clínico.

**Hallazgos Polisomnográficos de 4 Pacientes con Síndrome de Kleine Levin (KLS), Durante y Fuera de los Episodios**

Carmen Blázquez Muñoz^1^, Maria Jose Jurado Luque^1^, Rose Elena Cambrodí Masip^1^, Alex Ferré Maso^1^, Odila Romero Santo-Tomás^1^

^1^Hospital Universitario Vall d´Hebron, Barcelona, España

**Introducción y objetivos**: El síndrome de Kleine Levin es una enfermedad neurológica rara caracterizada por episodios recurrentes y autolimitados de hipersomnia, hiperfagia e hipersexualización, típicamente de inicio en la adolescencia y cuya fisiopatología no es bien conocida hasta el momento. **Material y métodos**: Se trata de un estudio retrospectivo y descriptivo que se realizó comparando las exploraciones polisomnográficas (15–24 horas) de pacientes diagnosticados de KLS en nuestro centro entre 2010 y 2023, durante los episodios sintomáticos y asintomáticos. Para ello se analizaron datos clínicos y neurofisiológicos: tiempo total de sueño (min), eficiencia de sueño (%), porcentajes de sueño NREM (N1, N2, N3) y REM, latencia de sueño, latencia de sueño REM y parámetros electroencefalograma (EEG). **Resultados**: La muestra incluyó 4 pacientes, 3 hombres y 1 mujer, de entre 16 y 59 años. Durante los ES, el tiempo total de sueño osciló entre 627,5 y 927,5 minutos, con un valor medio de 762,5. La eficiencia media del tiempo total de sueño fue de 61,1% (82,12% durante el sueño nocturno y 35,18% durante el sueño diurno). Durante los EA, la eficiencia media del tiempo total de sueño fue del 56%. Comparando ES y EA en 3 pacientes, el valor medio de la eficiencia de sueño total fue del 57,6% y del 56,3% en ES y EA, respectivamente. El porcentaje de sueño NREM y REM varió entre pacientes. Durante los ES, en todos los pacientes la latencia de sueño disminuyó, la latencia REM aumentó en 2/3 y 1/3 no cambió. **Discusión/Conclusiones**: En nuestro estudio, la eficiencia de sueño no empeoró de manera sistemática durante los ES, tal y como se ha reportado en la literatura previa. Además, la arquitectura del sueño nocturno y diurno mostró características diferentes en los episodios sintomáticos y asintomáticos. Estos resultados sugieren que la realización de PSG tanto durante como fuera de los episodios, y en análisis por separado de los periodos de sueño nocturno y diurno, quizás podrían ayudar a una mejor comprensión de este síndrome.

